# Synthesis of Thiacalix[4]arene Skeleton by the Conjugate Addition of Benzoquinone

**DOI:** 10.1021/acs.joc.5c00405

**Published:** 2025-04-30

**Authors:** Kamil Mamleev, Nicolai Nikishkin, Jan Čejka, Václav Eigner, Karolína Salvadori, Hana Dvořáková, Pavel Lhoták

**Affiliations:** † Department of Organic Chemistry, University of Chemistry and Technology Prague (UCTP), Technická 5, 166 28 Prague 6, Czech Republic; ‡ Department of Solid State Chemistry, UCTP, Technická 5, 166 28 Prague 6, Czech Republic; § J. Heyrovský Institute of Physical Chemistry, Academy of Sciences CR, 182 23 Prague 8, Czech Republic; ∥ Department of Analytical Chemistry, UCTP, Technická 5, 166 28 Prague 6, Czech Republic; ⊥ Laboratory of NMR Spectroscopy, UCTP, Technická 5, 166 28 Prague 6, Czech Republic

## Abstract

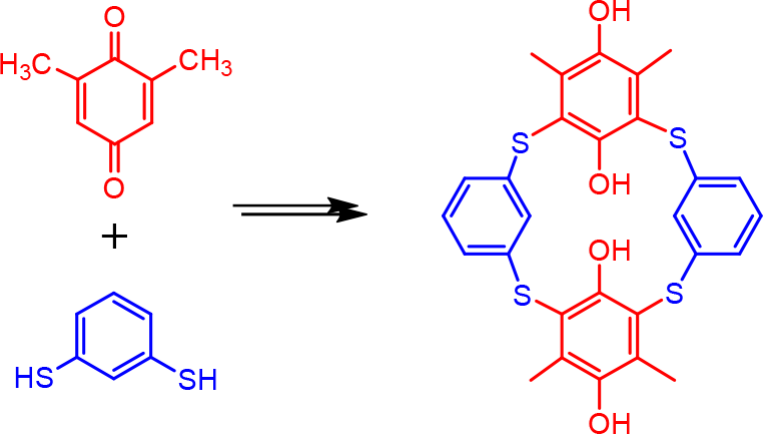

Macrocyclic systems having a thiacalix[4]arene-like structure with four bridging sulfur atoms can be easily constructed using benzoquinone and dithiol-based building blocks. The conjugate addition of benzene-1,3-dithiol with two equivalents of 2,6-dimethylbenzoquinone afforded the corresponding hydroquinone trimer, which, after oxidation to benzoquinone, undergoes a final macrocyclization with another benzene-1,3-dithiol molecule. The whole sequence represents a new strategy for the synthesis of macrocycles based on thiacalix[4]arenes. The conformational behavior of these macrocycles was studied using nuclear magnetic resonance and X-ray analyses, and their basic redox properties were investigated with electrochemical methods.

## Introduction

Current supramolecular chemistry comprises a great abundance of various macrocyclic compounds, such as crown ethers, calixarenes, porphyrins, cyclodextrins, etc.^1^ Due to their preorganization, these compounds are destined to fulfill the role of various receptors, complexing agents, self-assembly systems or molecular scaffolds for the synthesis of more sophisticated systems. Thus, the synthesis of new macrocycles, exhibiting potentially new properties, remains an important task for organic and supramolecular chemists. The replacement of methylene bridges with sulfur atoms resulted into a new group of macrocycles called thiacalixarenes.^2^ Comparing these compounds with the parent calixarenes,^3^ it is clear that the introduction of heteroatoms has led to a whole range of new properties, such as altered complexation ability, different conformational preferences, and a different type of chemistry that is not accessible with classical calixarenes.^4^

**Figure 1:**
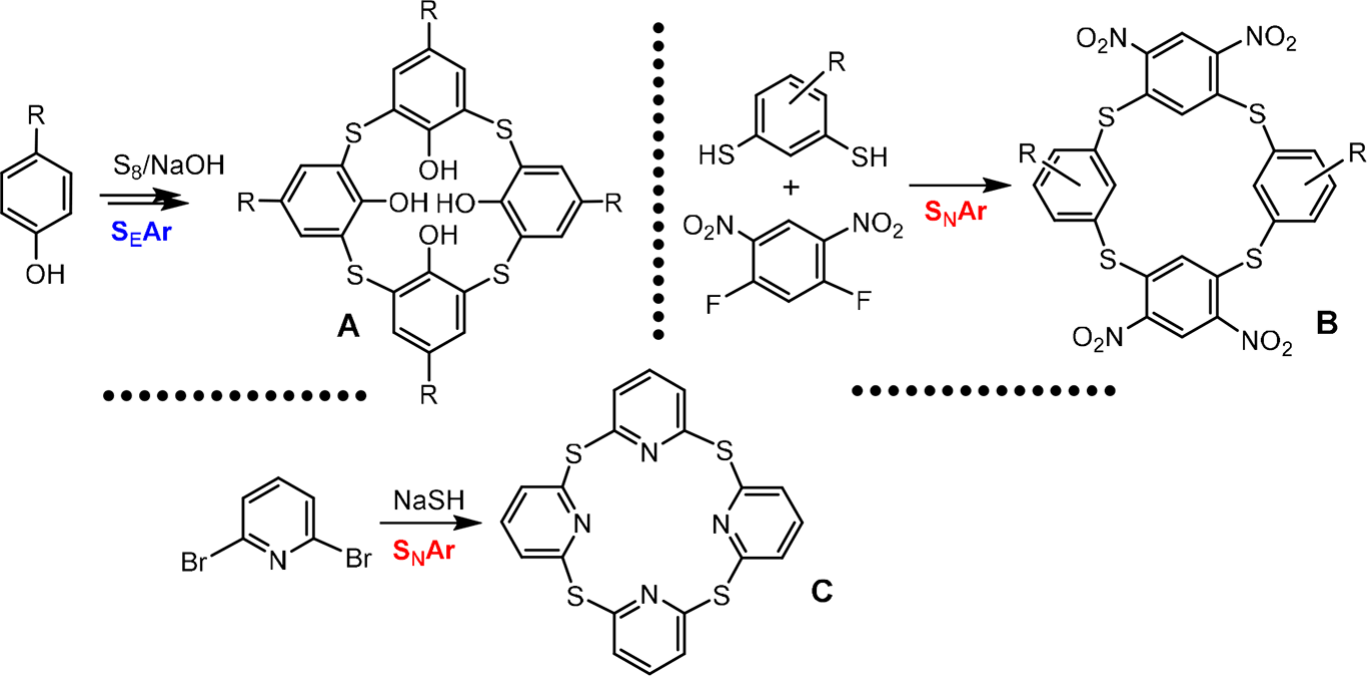
Previously reported synthetic approaches to calix[4]arenes and related macrocyclic systems bearing sulfur atoms as bridging units.

As shown in Figure 1, thiacalix[4]arenes **A** can be produced by direct electrophilic substitution of starting *para*-substituted phenols with elemental sulfur.^5^ Using this reaction, derivatives with various alkyl residues have been prepared, including *tert*-butyl, *tert*-octyl, phenyl etc. Structurally related derivatives **B** belonging to the sulfur analogues of [1_4_]metacyclophanes were prepared by aromatic nucleophilic substitution^6^ of the respective building blocks, substituted thioresorcinol and 1,5-difluoro-2,4-dinitrobenzene. Finally, a similar type of compounds **C** (thiacalix[4]pyridines) has been prepared^7^ in low yield (8%) (together with a trimer and hexamer) by a one-pot reaction of 2,6-dibromopyridine with sodium hydrogensulfide in 1,2-propanediol at 130 °C.

**Figure 2:**
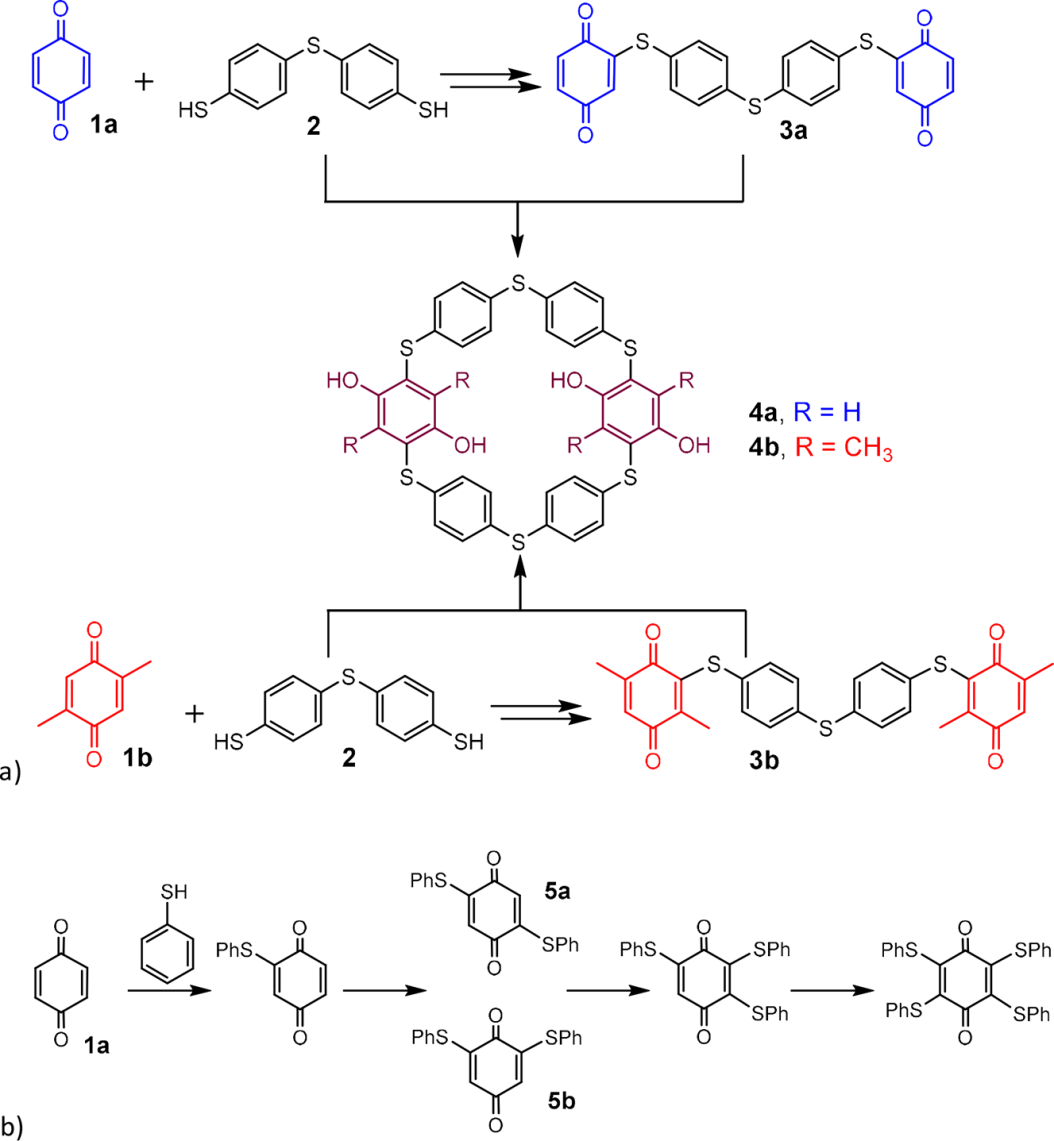
(a) Two approaches to thiapillar[6]arene macrocycles based on the thiol/quinone conjugate addition. (b) Nonselective addition of thiol to quinone moiety.

Very recently a novel approach employing the conjugate addition chemistry based on a thiol/benzoquinone system^8^ appeared in the literature. As shown in Figure 2a, a novel thiapillar[6]arene derivative **4a** was prepared starting from *p*-benzoquinone **1a** and dithiol **2**.^9^ The reaction sequence includes the formation of the key intermediate **3a**, which is finally cyclized by the addition of another molecule **2**. However, this step also represents a weakness of the entire procedure, because the addition of the thiols to quinone **3a** is not sufficiently regioselective, as can be judged from the reaction of *p*-benzoquinone **1a** with thiophenols (Figure 2b) leading to the formation of regioisomers **5a** and **5b**.^10^ The amounts of byproducts in the cyclization reaction can be reduced by using substituted quinones. As shown in Figure 2a, the usage of 2,5-dimethylbenzoquinone **2b** led to the formation of intermediate **3b**, in which only one position on each quinone moiety is available for further addition of thiol **2**. Macrocycle **4b** was thus prepared in very good yield.^11^

**Figure 3:**
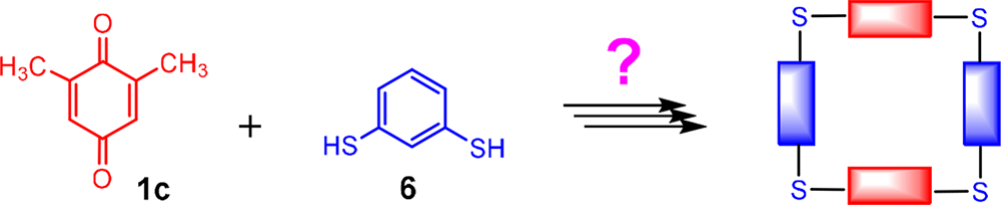
Synthesis of thiacalix[4]arene analogues based on precursors with suitable geometry.

Based on these findings, we came to the conclusion that the use of appropriately adapted building blocks could also lead to the synthesis of thiacalix[4]arene analogues, i.e. to macrocycles with bridging sulfur atoms and four aromatic units. As shown in Figure 3, 2,6-dimethylbenzoquinone **1c** and 1,3-benzenedithiol **6** as the building blocks are very well suited for the formation of a four-membered macrocycle. This paper deals with the synthesis of such compounds and the subsequent derivatization of their basic skeleton. To the best of our knowledge, this is the first example demonstrating the application of conjugate addition for the synthesis of these macrocycles.

## Results and Discussion

**Scheme 1:**
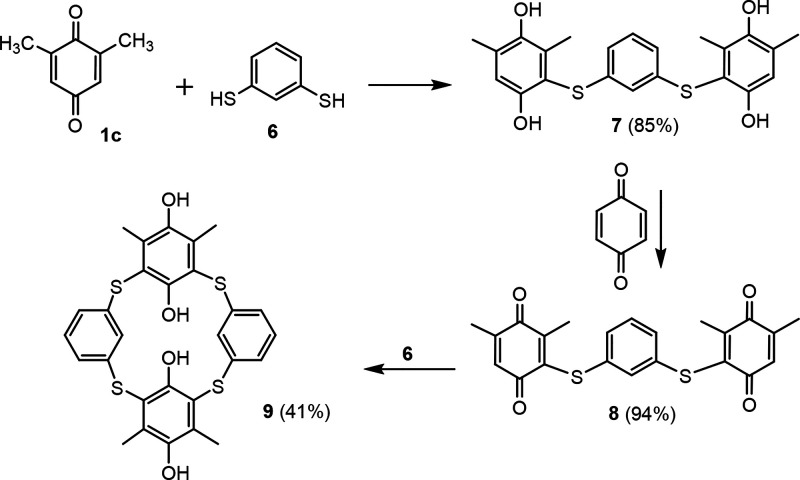
Synthesis of Compounds Studied

The synthesis of macrocyclic compound **9** is depicted in Scheme 1. A solution of 2,6-dimethyl-1,4-benzoquinone **1c** in MeOH was reacted with 1,3-benzenedithiol **6** (in 2.2:1 molar ratio) to provide the corresponding trimer **7** in 85% yield. The isolation of product does not require chromatographic purification (only trituration of the crude evaporated reaction mixture with DCM), so the reaction is also suitable for a larger multigram scale. Compound **7**, as a product of the double 1,4-conjugate addition, is then oxidized with *p*-benzoquinone at rt in acetone to yield the trimeric bis-quinone **8** almost quantitatively (94% isolated yield). The final cyclization of **8** with the second molecule of **6** proved to be very sensitive to the exact reaction conditions. Thus, simultaneous dropwise addition of the solutions of both reactants (the same concentration and volume), to the reaction mixture proved to be effective. These pseudohigh dilution conditions afforded macrocycle **9** in 41% yield, again without chromatographic isolation.

The ^1^H NMR spectrum of **9** (CDCl_3_, 400 MHz, 298 K) revealed two singlets for phenolic OH groups (8.43 and 7.86 ppm) together with one singlet for all methyl groups (2.20 ppm) reflecting the expected high symmetry of the structure. The molecular mass found in the HRMS (ESI^+^) spectrum (*m*/*z* = 575.0448) also well agreed with the value predicted for [**9**+Na]^+^ ion (*m*/*z* = 575.0450, C_28_H_34_O_4_S_4_Na). As revealed by a VT ^1^H NMR study (500 MHz, DMF-*d*_7_), cooling the solution from room temperature (323 K) down to 193 K did not lead to any change in the number of signals, and the splitting pattern remained unchanged—see Figures S47 and S48. This indicates that the molecule either occupies the same conformation through the entire temperature range or possesses high conformational mobility. Due to the impossibility of forming a cyclic array (missing OH groups) of hydrogen bonds at the lower rim of the molecule, as is the case with classical calixarenes, high conformational mobility is very likely.

**Figure 4:**
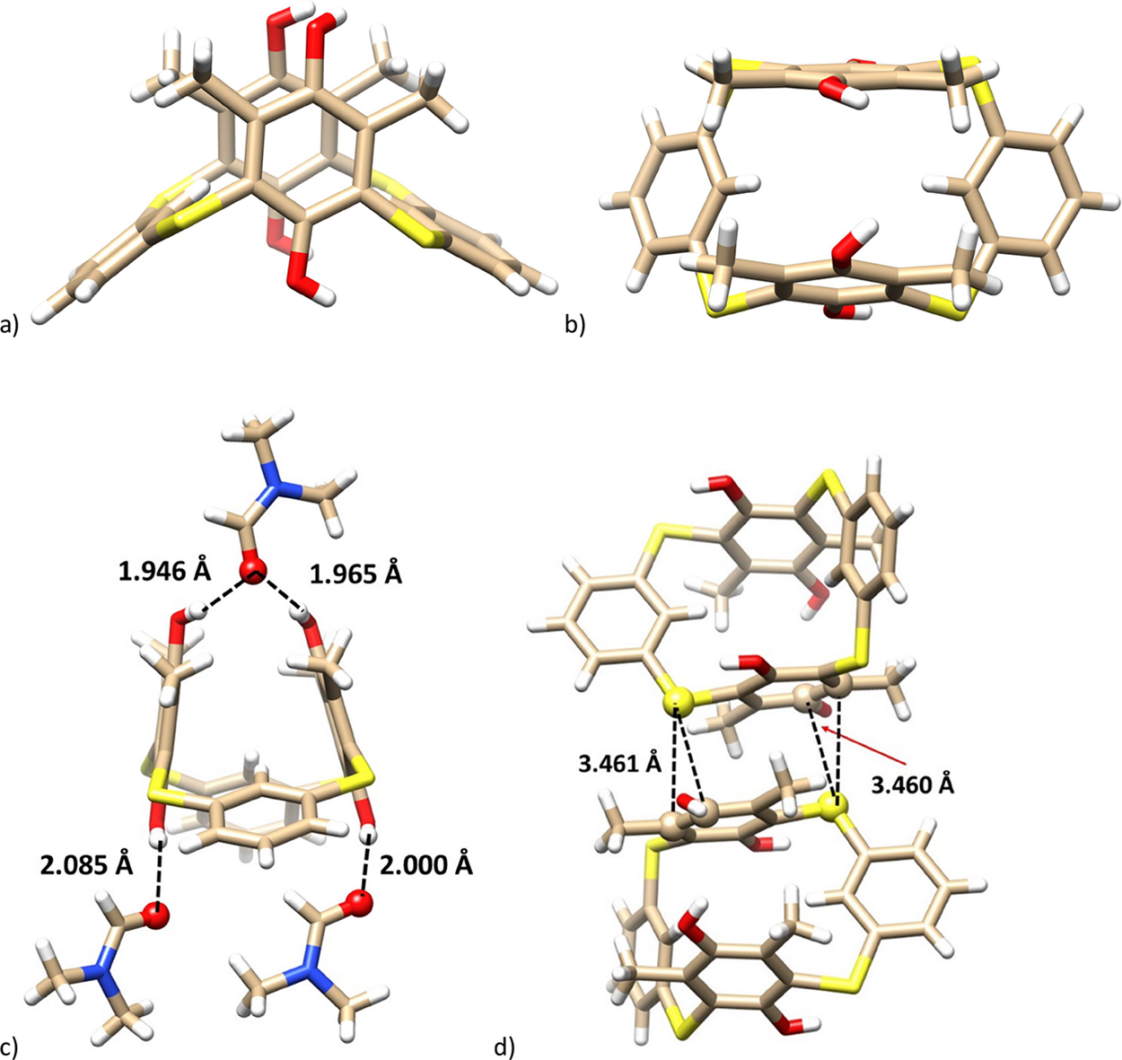
Single crystal X-ray structures of compound **9**: (a) side-view; (b) top-view; (c) the hydrogen bonding of DMF molecules; (d) a dimeric motif of **9** showing chalcogen S···C(arom) interactions (the interacting atoms shown as balls).

The unequivocal evidence of the structure was obtained by single crystal X-ray analysis. Slow crystallization from DMF provided **9** as a solvate with three molecules of DMF (triclinic system, space group *P*1̅). As shown in Figure 4a,b, the molecule adopts the *1,3-alternate* conformation (using common calixarene nomenclature) where both unsubstituted rings are tilted into the cavity, while the hydroquinone moieties are approximately coplanar (mutual interplanar angle = 156.85°). All four phenolic hydroxyls participate in the binding of DMF molecules through hydrogen bonds (Figure 4c). The respective C=O···H–O distances (1.946–2.085 Å) indicate the strength of these interactions.^12^ The crystal packing also contains an interesting dimeric motif based on the chalcogen bond^13^ between the sulfur atom and the adjacent hydroquinone moiety (S···C distances of 3.461 and 3.460 Å) representing the η^2^ chalcogen-aromatic interactions^14^ (Figure 4d).

**Scheme 2:**
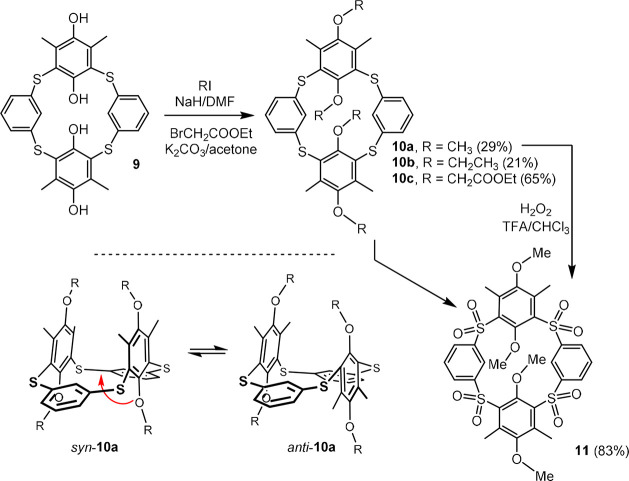
Derivatization of Thiacalixarene **9** and the *syn*-*anti* Equilibrium for Compound **10a**

The resulting macrocycle **9** relatively quickly deteriorates (within a few hours) upon exposure to air as a result of oxidation to benzoquinone derivative. Although we obtained the corresponding molecular peak in HRMS analysis, we were never able to isolate this compound in pure state, probably due to the low stability of the cyclic quinone structure. To prevent oxidation, an alkylation of phenolic −OH groups was carried out using the RI/NaH reaction system which is commonly used in calixarene chemistry (Scheme 2). The reaction was carried out overnight in DMF at 70 °C with vigorous stirring. The corresponding methoxy and ethoxy derivatives **10a** and **10b** were obtained after column chromatography on silica gel in 29 and 21% yields, respectively. The HRMS (ESI^+^) spectrum of **10a** revealed the peaks at *m*/*z* = 631.1074 and *m*/*z* = 647.0813 corresponding to the expected [M + Na]^+^ and [M + K]^+^ ions (*m*/*z* = 631.1076 and 647.0815). Interestingly, the ^1^H NMR spectrum of **10a** (CDCl_3_, 400 MHz) showed two sets of signals which are best visible for methoxy and methyl groups. Thus, two singlets of CH_3_-arom at 2.24 and 2.08 ppm in approximately 3.5:1 ratio together with two sets of singlets for OCH_3_ groups at 3.57/3.53 ppm (major) and 3.43/3.38 ppm (minor) in the same 3.5:1 ratio indicated the presence of two conformers in solution at room temperature. A very interesting difference in chemical shifts can be observed for the intra-annular H atom (aromatic CH bond between the two sulfur bridges). The major conformer has a chemical shift of 5.77 ppm, while the minor peak is located at 6.30 ppm. Both upfield shifts indicate a strong shielding by neighboring aromatic units (hydroquinones).

**Figure 5:**
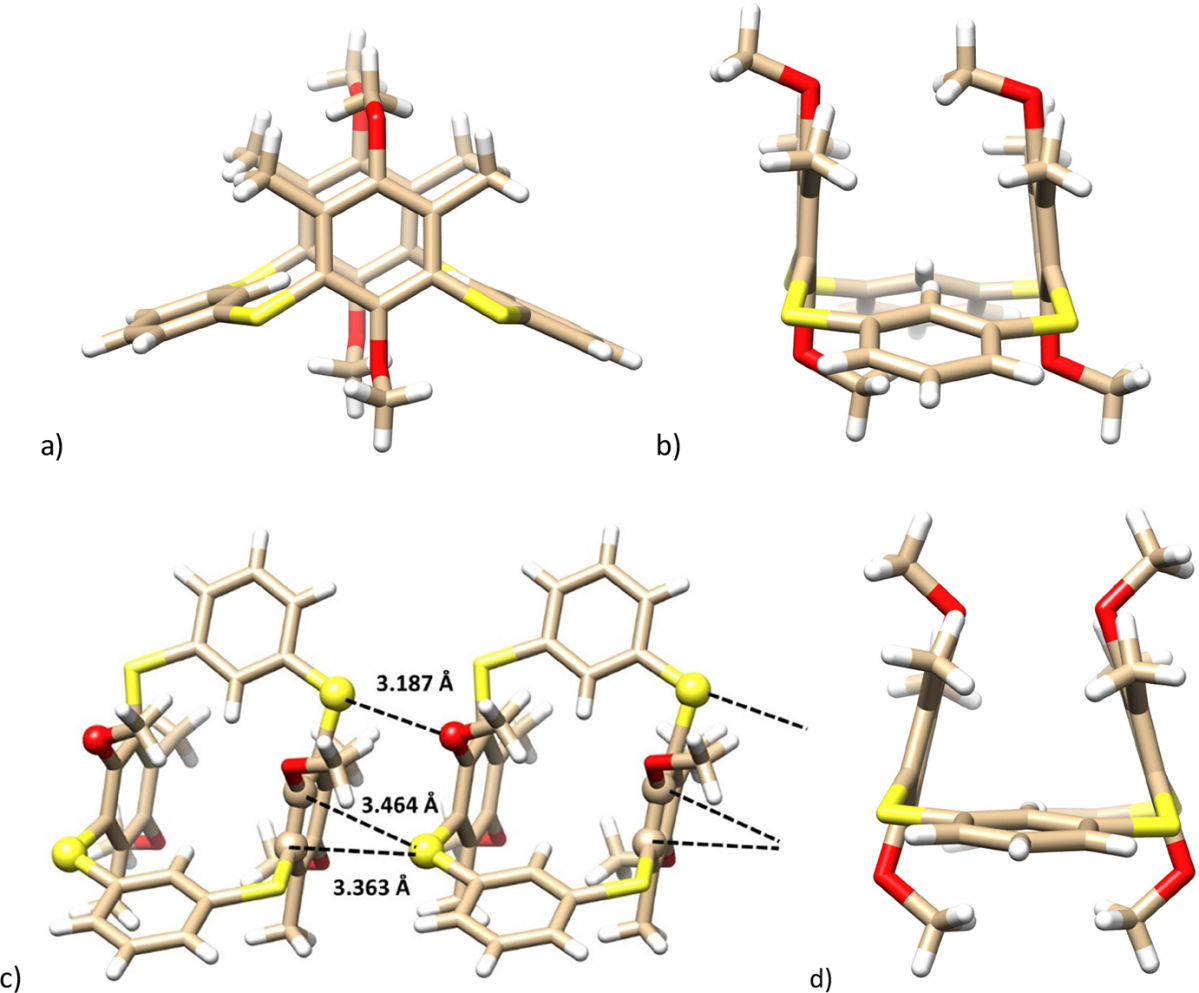
Single crystal X-ray structures of compound **10a**: (a) side-view; (b) side-view (rotated by 90°); (c) binding motif of **10a** showing chalcogen S···O and S···C(arom) interactions (the interacting atoms shown as balls). (d) X-ray structure of polymorphic form **10a2**.

The single-crystal X-ray study of **10a** provided the final unambiguous structural evidence. Compound **10a** crystallized in the monoclinic system as a single conformer, space group C*2*/c. As shown in Figure 5a, two unsubstituted aromatic moieties are extremely flattened with the corresponding interplanar angles Φ of 19.26° and 24.41° (toward the main plane of the molecule defined by the four sulfur atoms). The remaining hydroquinone moieties are nearly perpendicular to the main plane with Φ angles of 83.18° and 89.87°, resulting in an almost parallel mutual arrangement (Figure 5b). Using the established nomenclature for classical calix[4]arenes the adopted conformation could be described as being *1,3-alternate*.

An interesting binding motif was found in the crystal packing of **10a**, where the individual molecules are bound to each other by means of the S···O chalcogen interaction (3.187 Å, the sum of the van der Waals radii^15^ for O and S atoms is 3.32 Å). At the same time, the whole arrangement is completed by the close contacts between the sulfur bridge and two aromatic carbon atoms of the adjacent hydroquinone moiety (η^2^ chalcogen-aromatic interaction) with the corresponding S···C distances of 3.464 and 3.363 Å (Figure 5c).

Interestingly, crystallization from chloroform-ethyl acetate gave single-crystals of polymorphic form **10a2**. This polymorph crystallized in the monoclinic system, space group *R*3̅*c*, and contained solvent molecules with a 1:3 stoichiometry (CHCl_3_: **10a**). The basic structural features and packing pattern are very similar to the above-mentioned X-ray structure with unsubstituted aromatic groups being even more flattened (Φ angles of 19.53° and 9.76°). The biggest difference can be seen in the orientation of the methyl groups on the hydroquinone cores (see Figures 5d, S79–S81).

**Figure 6:**
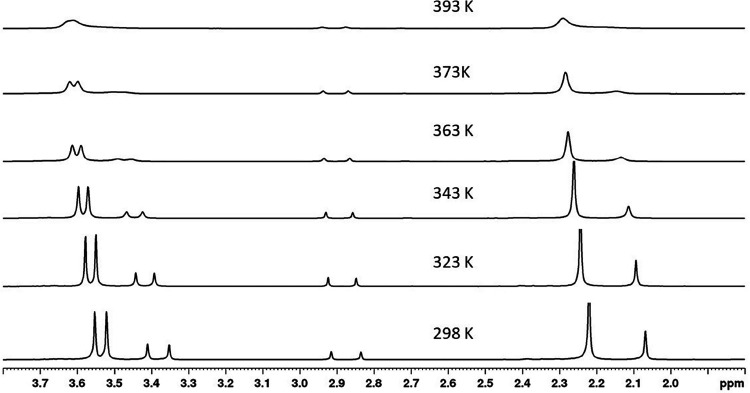
Variable temperature ^1^H NMR study of **10a** (500 MHz, CDCl_2_–CDCl_2_): partial ^1^H NMR spectra showing the area of CH_3_ and OCH_3_ signals (singlets at 2.84 and 2.92 ppm are from residual DMF).

X-ray structural analysis also indicated the structure of the conformers observed in the NMR spectrum at room temperature. Obviously, the unsubstituted aromatic moieties are free to move through the cavity, so the only option lies in the inverted arrangement of the alkylated hydroquinone groups. Thus, if we imagine the unsubstituted subunits as approximately planar, the remaining aromatic moieties adopt either a *syn*- or an *anti*- arrangement with respect to the plane of the molecule (Scheme 2). The variable temperature ^1^H NMR spectra of **10a** in 1,1,2,2-tetrachloroethane-*d*_2_ (500 MHz) showed that with increasing temperature, coalescence occurred (at approximately 380 K), and only one set of signals appeared for the Me and OMe groups, indicating fast exchange conditions (Figure 6).

The corresponding ethoxy derivative **10b** showed only one set of signals in the ^1^H NMR spectrum and its structure was eventually confirmed by X-ray analysis of the single-crystal (MeOH–CH_2_Cl_2_). Compound **10b** crystallized in the triclinic system, space group *P*1̅, as a 2:1 complex (calix:MeOH) with solvent molecule. The basic geometrical parameters of both independent calixarene molecules occurring in the unit cell (see Figures S82–S84) are very similar to those of compound **10a**. Again, very flattened aromatic cores (Φ = 17.20° and 20.39°/17.81° and 12.77° for the two independent molecules) and nearly perpendicular hydroquinone units confirmed that the *syn* conformation (see Figure 5) is the kinetically preferred main product of the alkylation. The same can be said for the corresponding tetraester **10c**, which also shows only one set of signals in the ^1^H NMR spectrum corresponding to the *syn* -conformer (characteristic signal of shielded CH bond at 5.70 ppm).

An ^1^H NMR screening of the supramolecular behavior of compounds **10a** was performed. All attempts to use compound **10a** for complexation (CDCl_3_) of substrates bearing acidic methyl groups, such as nitromethane, MeCN, or toluene, failed. The same applies to the use of a quaternary ammonium salt (*N*-methylpyridinium iodide). It seems that these systems are not suitable for exploiting the possible CH−π^16^ or cation−π interactions^17^ that are quite common in classical calixarenes. A possible explanation is that the preferred conformation of compound **10a** is not the *cone*, which is the most suitable shape for these types of interactions.

In order to demonstrate further derivatization of the basic skeleton, the oxidation of the methoxy derivative **10a** was carried out using the H_2_O_2_/TFA/CHCl_3_ system (Scheme 2). The corresponding tetrasulfone **11** was isolated in 83% yield after heating the reaction mixture for 4 days at 62 °C. The molecular peak of **11** in its HR MS (ESI^+^) spectrum (*m*/*z* = 759.0669) well agreed with the mass expected for the [M + Na]^+^ ion (*m*/*z* = 759.0669). The aromatic part of the ^1^H NMR spectrum (CDCl_3_, 500 MHz) showed broad diffuse peaks at room temperature indicating a dynamic behavior of the molecule. On the other hand, the aliphatic part of the spectrum exhibited one singlet for the CH_3_- groups at 2.83 ppm and three sharp singlets for the OCH_3_ groups at 3.73, 3.78, and 4.09 ppm. While the ratio of the corresponding integrals (CH_3_ vs OCH_3_ signals) was 1:1 (as expected), the number of signals (three singlets) for the methoxy groups was unexpected because it did not correspond to any possible single conformation.

**Figure 7:**
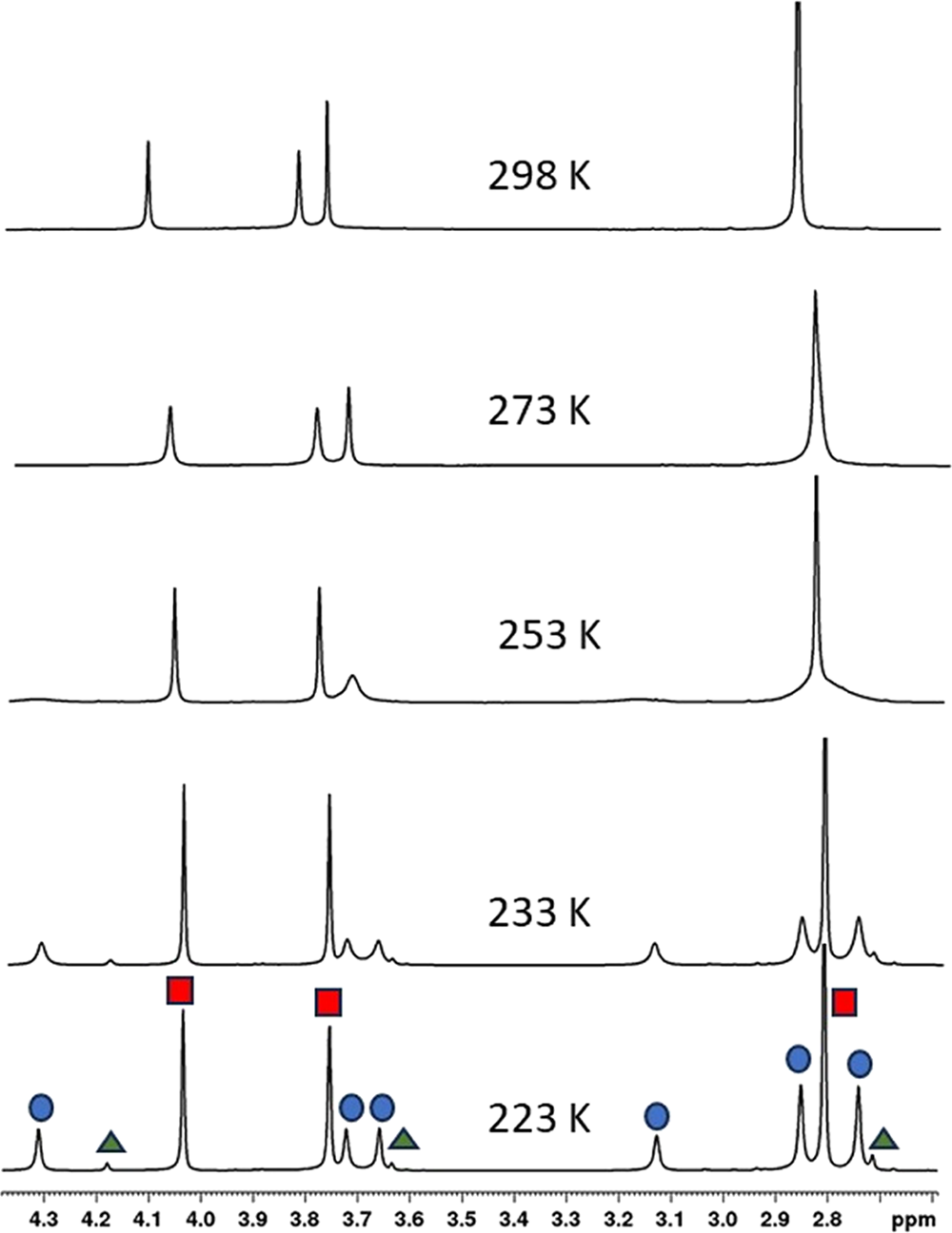
Variable temperature ^1^H NMR study of **11** (500 MHz, CD_2_Cl_2_): partial ^1^H NMR spectra showing the area of CH_3_ and OCH_3_ signals (2.6–4.4 ppm).

The variable temperature ^1^H NMR study of compound **11** in CD_2_Cl_2_ solution (Figure 7) revealed that cooling led to a significant broadening of certain peaks due to slower chemical exchange. The peak at 3.73 ppm gradually completely disappeared and new peaks emerged from the baseline. The coalescence of this phenomenon occurs approximately around 250 K. Below this temperature, a new set of four singlets (3.13, 3.66, 3.72, and 4.31 ppm) appeared, representing four signals of OCH_3_ groups from another conformer of the compound. At the same time, a new pair of singlets for this conformer separated at 2.74 and 2.85 ppm from the original singlet of the CH_3_ group. The spectrum acquired at 213 K also showed (Figure 7) the presence of another set of minor singlets (2.71, 3.63, and 4.18 ppm) corresponding to yet another conformation of compound **11**. As a result, it can be concluded that the ^1^H NMR spectrum of **11** is reflecting the thermodynamic equilibrium of at least three different conformations, which, however, could not be unambiguously assigned.

**Figure 8:**
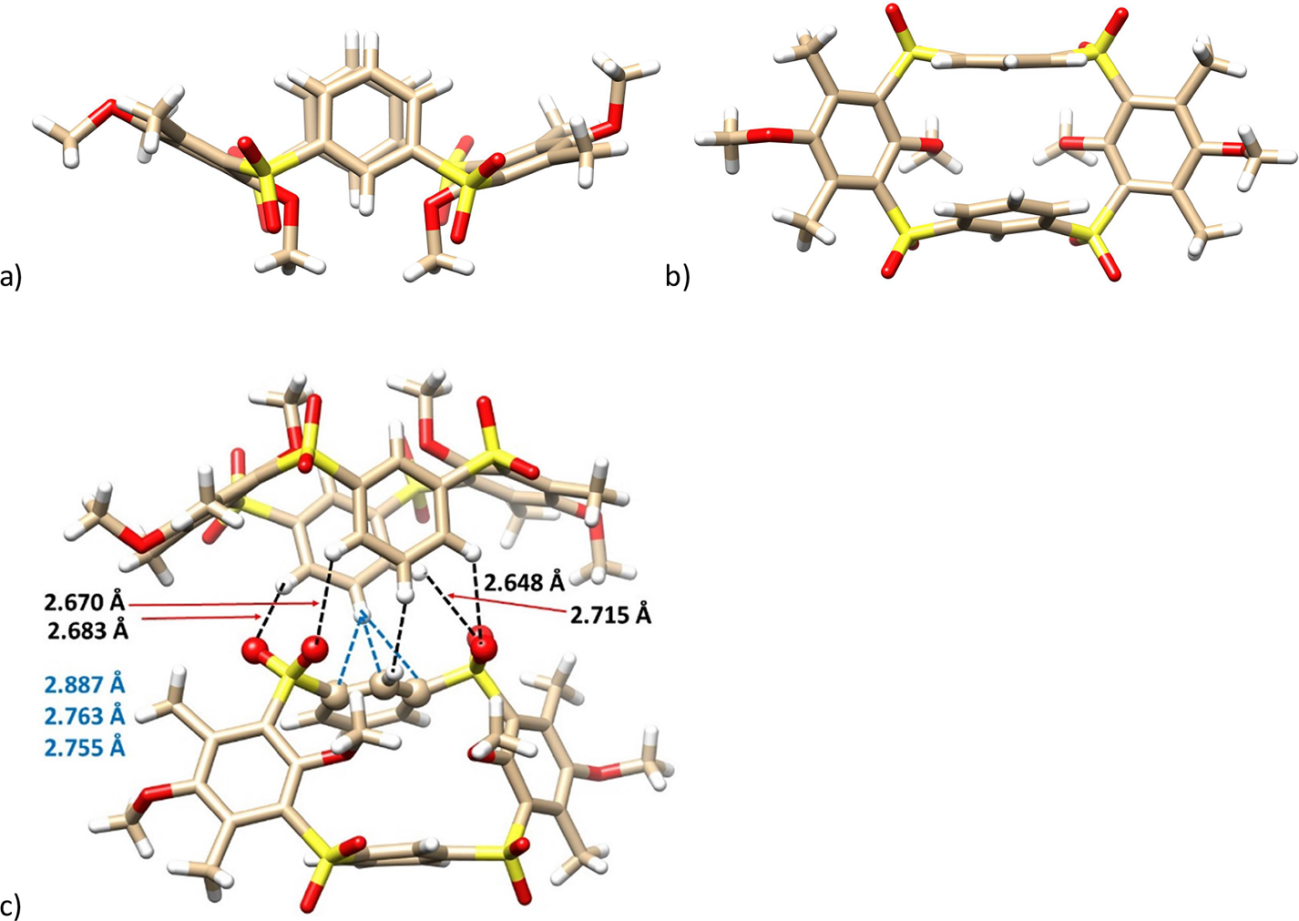
Single crystal X-ray structures of compound **11**: (a) side view; (b) top view; (c) binding motif of **11** showing the array of S=O···H–C(arom) and the T-shape π–π interactions (blue) (the interacting atoms shown as balls).

Final proof of the structure was provided by X-ray structural analysis. Compound **11** crystallized in the monoclinic system, space group P 2_1_/n, as a 1:1 complex with chloroform. Unlike all previous derivatives, the molecule surprisingly adopts the *pinched cone* conformation. Another obvious difference is the arrangement of the unsubstituted aromatic cores, that are almost perpendicular (Φ = 76.06° and 86.88°) to the main plane of the molecule (represented by four sulfur bridges), while the hydroquinone units are tilted out of the cavity at a high angle (Φ = 152.45° and 153.94°)—see Figure 8a,b. The crystal arrangement is based on an interesting bonding motif in which two neighboring molecules are held together by hydrogen bonding between the oxygens of the sulfone groups and *meta* CH bonds (four interactions with the S=O···H–C distances of 2.652 to 2.695 Å) of unsubstituted aromatic units (Figure 8c). This is further complemented by the T-shaped aromatic interactions^18^ between the *para*-CH bond and the neighboring aromatic subunit exhibiting the η^3^ binding mode (C–H··· C_arom_ distances = 2.752, 2.779, and 2.892 Å.

As the studied compounds possess redox active sites, we decided to evaluate their properties using standard electrochemical methods (RDE and CV). At the beginning of the electrochemical investigation, the behavior of suiatable model systems was studied. In the case of quinones, the 2,6-dimethyl quinone **1c** was selected. This quinone undergoes two successive one-electron reduction steps to produce semiquinone (**1c**^•–^) and quinone dianion (**1c**^2–^). By measuring the cyclic voltammograms, two separate cathodic peaks could be observed, in which the first step is completely reversible and the second step is quasi-reversible (Figures S54–S57). The same two-step process was evident also in the case of acyclic intermediate **8** with both reduction potentials slightly shifted toward less negative values (Table 1). According to the CV, compound **8** behaves in the same way as model quinone (Figures S59–S61), although the peak′s reversibility was less pronounced than in **1c**.

**Table 1:** Values of Redox Potentials *E* (V) Evaluated from Appropriate Records of Linear Sweep Voltammetry

type of structure	compound	*E* (V) ox	*E*_1_ (V) red	*E*_2_ (V) red
hydroquinone	2,6-Dimethyl-1,4-benzenediol	0.57		
	**7**	0.67	–2.51	
	**9**	0.80	–2.33	–2.67
quinone	**1c**		–0.55	–1.31
	**8**		–0.38	–1.17

**Figure 9:**
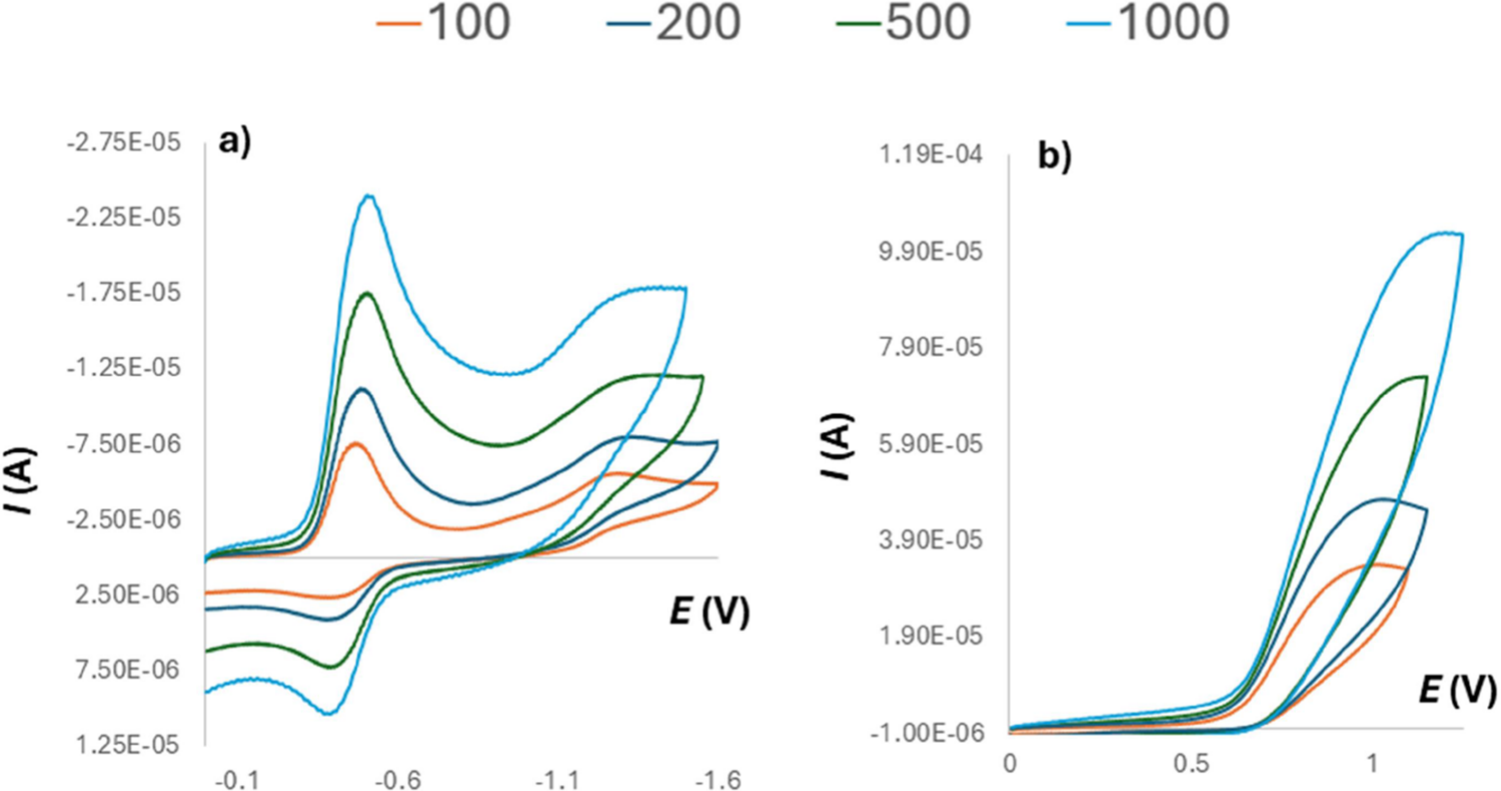
Cyclic voltammograms (W = GC, ref = SCE, Aux = Pt): (a) reduction of compound **8** (1.00 mM) in DMSO (0.1 M *n*-Bu_4_^+^PF_6_^–^) measured with several different scan rates (polarographic plotting convention); (b) oxidation of macrocycle **9** (1.06 mM) in DMSO (0.1 M *n*-Bu_4_^+^PF_6_^–^) measured with several different scan rates (IUPAC plotting convention).

In addition, the redox properties of the hydroquinone group were monitored using 2,6-dimethylhydroquinone as a model compound. Here, only a one-step two-electron oxidation response was observed. Similarly, the electrochemical oxidation of the acyclic intermediate **7** and the macrocycle **9** was studied. Again, we found out that they behave the same as model system (Figures 9 and S65–S76). Surprisingly, it was found that in addition to the typical one-step irreversible oxidations (Table 1) observed with the model compound, reduction of both compounds also occurs. The reduction was evident at highly negative potentials i.e. −2.51 V for **7**. A similar behavior has already been noted in the case of analogous thiapillararenes^11^ and can be explained as being due to the reduction of phenolic protons.

## Conclusions

In conclusion, we have demonstrated the usefulness of conjugate addition for the construction of macrocyclic systems. Using suitable building blocks—2,6-dimethylbenzoquinone and benzene-1,3-dithiol—an analogue of thiacalix[4]arene was successfully prepared and its basic derivatization was performed, including alkylation of free phenolic hydroxyls and oxidation of sulfur bridges. While the structures of compounds in the solid state were confirmed by X-ray structural analysis, VT ^1^H NMR spectroscopy showed interesting dynamic behavior of the molecules in solution. This work has demonstrated that the chosen synthetic procedure (conjugated addition of benzoquinone derivatives) represents a new alternative strategy to the synthesis of similar compounds, the further use of which may lead to a number of new macrocyclic systems.

## Experimental Section

### General Information

All chemicals were purchased from commercial sources and used without further purification. THF and CH_3_CN were dried using a column solvent purification system PureSolv MD7 (Inert). Melting points were measured on a Heiztisch Mikroskop Polytherm A (Wagner & Munz), and they are not corrected. The ^1^H and ^13^C{^1^H} NMR spectra were recorded on an Agilent 400-MR DDR2 and JEOL-ECZL400G (^1^H: 400 MHz, ^13^C: 100 MHz). The ^1^H VT NMR experiments were carried out on a Bruker Avance III 500 MHz. The chemical shifts (δ) are reported in parts per million (ppm) and were referenced to the residual peaks of the solvent or TMS as an internal standard; the coupling constants (*J*) are expressed in Hz. All the NMR data were processed and displayed using MestReNova software.

The FTIR analysis was performed on a Nicolet iS50 spectrometer (Thermo-Nicolet, USA) connected with a GladiATR diamond placed outside the conventional sample compartment, equipped with DTGS KBr detector. Reflectance data were acquired with the following parameters—spectral range: 4000–400 cm^–1^, resolution: 4 cm^–1^, number of spectra accumulations: 64, apodization: Happ-Genzel. The spectra were collected and processed by Omnic 9 (Thermo-Nicolet Instruments Co., USA) including baseline correction and Savitzky-Golay smoothing filter (set to the number of 11 points used in the algorithm). The resulting spectrum is the average of three independent measurements.

ESI HRMS spectra were measured on an LC–MS LTQ-Orbitrap Velos (Thermo) spectrometer. Substance purities and the reaction progress were monitored by thin layer chromatography (TLC) using silica gel 60 F_254_ on aluminum-backed sheets (Merck) and analyzed at 254 and/or 365 nm. Radial chromatography was carried out on Chromatotron (Harrison Research) connected with a lab pump RHSY2 (Fluid Metering). Self-prepared glass discs were covered by silica gel 60 PF_254_ containing CaSO_4_ (Merck). Self-prepared glass plates for preparative TLC (20 × 20 cm) were covered by silica gel 60 PF_254_ containing CaSO_4_ (Merck). All heating (unless otherwise stated) was performed using heating blocks of appropriate size.

The starting benzene-1,3-dithiol **6** was prepared by modification of the published procedure^19^ (see SI).

#### Compound **7**

To a solution of 2,6-dimethyl-1,4-benzoquinone (**1c**) (2.99 g, 22.0 mmol,) in 50 mL of MeOH a solution of 1,3-benzenedithiol (1.15 mL 10.0 mmol) in 4 mL of DCM was slowly added dropwise via syringe under argon. The resultant mixture was stirred 24 h under argon followed by removal of all the volatiles under vacuum. The oily residue was triturated with DCM and the resulting white crystalline product was filtered off and with cold DCM. Compound **7** was obtained in 85% (3.52 g) yield as a white amorphous solid. Mp = 188–191 °C. ^1^H NMR (DMSO-*d*_6_, 400 MHz, 298 K) δ (ppm): 8.91 (s, 2H), 7.79 (s, 2H), 7.04–6.99 (m, 1H), 6.57–6.48 (m, 5H), 2.19–2.14 (m, 12H). ^13^C{^1^H} NMR (DMSO-*d*_6,_ 100 MHz, 298 K) δ (ppm): 152.3, 146.0, 139.1, 130.4, 129.1, 121.4, 121.2, 114.9, 112.4, 30.7, 17.1, 14.7. IR (ATR) ν: 3474, 3375, 1566, 1461 cm^–1^. HRMS (ESI^+^) *m*/*z*: [M + Na]^+^ calcd for C_22_H_22_O_4_S_2_Na 437.0852; found 437.0853.

#### Compound **8**

Compound **7** (0.97 g, 2.34 mmol) was dissolved in 25 mL of acetone. A solution of 1,4-benzoquinone (1.20 g, 11.10 mmol) in 10 mL of acetone was added and the reaction mixture was stirred for 24 h followed by removal of all the volatiles under vacuum. The black residue was sonicated in 30 mL of DCM and the solids were removed by filtration. The product was isolated by column chromatography on silica gel (eluent = gradient cyclohexane:DCM (from 1:0 to 1:2)). Compound **8** was obtained in 94% (0.90 g) yield as a dark red oil. ^1^H NMR (CDCl_3_, 400 MHz, 298 K) δ (ppm): 7.21–7.11 (m, 4H), 6.60–6.58 (m, 2H), 2.19–2.17 (m, 6H), 2.06–2.04 (m, 6H). ^13^C{^1^H} NMR (CDCl_3,_ 100 MHz, 298 K) δ (ppm): 185.9, 182.4, 148.0, 146.2, 142.1, 135.5, 134.1, 131.9, 129.9, 129.2, 16.2, 15.7. IR (ATR) ν: 2921, 1647, 1565 cm^–1^. HRMS (ESI^+^) *m*/*z*: [M + Na]^+^ calcd for C_22_H_18_O_4_S_2_Na 433.0539; found 433.0540.

#### Compound **9**

A solution of compound **8** (4.10 g, 10.0 mmol) in 100 mL of DCM and a solution of **6** (1.15 mL, 10.0 mmol) in 100 mL of DCM were simultaneously added dropwise to 100 mL of DCM in a three-necked flask under argon in course of 3 h. The resultant mixture was stirred for 3 days. The product was filtered off from the mixture and washed with DCM to provide compound **9** (2.25 g, 41%) as a white powder. Mp > 350 °C. ^1^H NMR (DMSO-*d*_6_, 400 MHz, 298 K) δ (ppm): 8.43 (s, 2H), 7.86 (s, 2H), 7.25–7.19 (m, 2H), 7.10–7.05 (m, 4H), 5.66 (t, *J* = 1.8 Hz, 2H) and 2.20 (s, 12H). ^13^C{^1^H} NMR (DMSO-*d*_6,_ 100 MHz, 298 K) δ (ppm): 153.7, 147.1, 138.8, 133.8, 129.0, 121.6, 117.2, 112.6, 15.2. IR (ATR) ν: 3349, 1566, 1455 cm^–1^. HRMS (ESI^+^) *m*/*z*: [M + Na]^+^ calcd for C_28_H_24_O_4_S_4_Na 575.0450; found 575.0448.

#### Compound **10a**

Compound **9** (0.51 g, 0.92 mmol) was dissolved in 80 mL of dry DMF. Sodium hydride (0.32 g, 8.00 mmol) and MeI (0,50 mL, 8.03 mmol) were added and the reaction mixture was stirred and heated at 70 °C for 1 day. The reaction mixture was quenched with water (100 mL) and the product was extracted with chloroform (3 × 60 mL). The organic phase was washed with water, dried over MgSO_4_ and separated by column chromatography on silica gel (eluent = cyclohexane:DCM = 1:3, v/v). Compound **10a** was obtained in 29% yield (0.16 g) as a white solid. Mp = 253–256 °C. ^1^H NMR (CDCl_3_, 400 MHz, 298 K) δ (ppm): 7.22–7.17 (m, 2H), 7.14–7.10 (m, 4H), 5.79 (t, *J* = 1.8 Hz, 2H), 3.58 (s, 6H), 3.55 (s, 6H), 2.26 (s, 12H). ^13^C{^1^H} NMR (CDCl_3,_ 100 MHz, 298 K) δ (ppm):129.2, 123.1, 122.5, 118.6, 62.6, 60.5, 14.8. IR (ATR) ν: 2948, 2922, 1571, 1453 cm^–1^. HRMS (ESI^+^) *m*/*z*: [M + Na]^+^ calcd for C_32_H_32_O_4_S_4_Na 631.1076; found 631.1074.

#### Compound **10b**

Compound **9** (0.21 g, 0.38 mmol) was dissolved in 30 mL of dry DMF. Sodium hydride (0.14 g, 3.50 mmol) and EtI (0,35 mL, 4.35 mmol) were added and the reaction mixture was stirred and heated at 70 °C for 1 day. Water (80 mL) was added to quench the reaction and the product was extracted with chloroform (3 × 50 mL). The organic phase was washed with water, dried over MgSO_4_ and separated by column chromatography on silica gel (eluent = cyclohexane:DCM = 1:3, v/v). Compound **10b** was obtained in 21% (55.2 mg) yield as a white solid. Mp = 219–221 °C. ^1^H NMR (CDCl_3_, 400 MHz, 298 K) δ (ppm): 7.21–7.16 (m, 2H), 7.14–7.09 (m, 4H), 5.80 (t, *J* = 1.8 Hz, 2H), 3.70–3.58 (m, 8H), 2.23 (s, 12H), 1.41 (t, *J* = 7.0 Hz, 6H), 1.14 (t, *J* = 7.0 Hz, 6H). ^13^C{^1^H} NMR (CDCl_3,_ 100 MHz, 298 K) δ (ppm): 160.7, 153.4, 140.5, 140.2, 129.1, 123.1, 122.5, 118.9, 71.0, 68.8, 15.7, 15.6, 14.9. IR (ATR) ν: 2974, 2921, 1567 cm^–1^. HRMS (ESI^+^) *m*/*z*: [M + Na]^+^ calcd for C_36_H_40_O_4_S_4_Na 687.1702; found 687.1705.

#### Compound **10c**

Compound **9** (0.10 g, 0.18 mmol) was dissolved in 15 mL of dry acetone. K_2_CO_3_ (0.22 g, 1.59 mmol) and methyl 2-bromoacetate (0.17 mL, 1.78 mmol) were added and the reaction mixture was stirred and heated at 52 °C for 1 day. Water (40 mL) was added and the product was extracted with chloroform (3 × 50 mL), the organic phase was washed with water, dried over MgSO_4_ and the product was recrystallized from a DCM/MeOH mixture. Compound **10c** was obtained in 65% (98.43 mg) yield as a white solid. Mp = 236–238 °C. ^1^H NMR (CDCl_3_, 400 MHz, 298 K) δ (ppm): 7.22–7.16 (m, 2H), 7.13–7.09 (m, 4H), 5.71 (t, *J* = 1.8 Hz, 2H), 4.37 (s, 4H), 4.25 (s, 4H), 3.81 (s, 6H), 3.60 (s, 6H), 2.30 (s, 12H). ^13^C{^1^H} NMR (CDCl_3,_ 100 MHz, 298 K) δ (ppm): 169.0, 168.6, 159.5, 152.9, 140.6, 139.5, 129.5, 123.8, 123.1, 118.6, 70.5, 69.9, 52.3, 52.1, 15.2. IR (ATR) ν: 2951, 2916, 1757, 1742, 1569 cm^–1^. HRMS (ESI^+^) *m*/*z*: [M + Na]^+^ calcd for C_40_H_40_O_12_S_4_Na 863.1295; found 863.1293.

#### Tetrasulfone **11**

Compound **10a** (0.14 g, 0.23 mmol) was dissolved in 6 mL of chloroform. Trifluoroacetic acid (3 mL) of and 30% aqueous hydrogen peroxide (5 mL) were added and the reaction mixture was stirred and heated at 62 °C for 4 days. The crude product was extracted with chloroform (3 × 40 mL). Organic layer was washed with water, dried over MgSO_4_ and evaporated to provide compound **11** in 83% yield (0.14 g) as a white solid. Mp = 236–238 °C. ^1^H NMR (CDCl_3_, 400 MHz, 298 K) δ (ppm): 8.60–8.53 (m, 1H), 8.02–7.98 (m, 1H), 7.72–7.58 (m, 2H), 7.32–7.26 (m, 2H), 7.16–7.03 (m, 2H), 4.11–3.71 (m, 12H), 2.84 (s, 12H). ^13^C{^1^H} NMR (CDCl_3,_ 100 MHz, 298 K) δ (ppm): 155.4, 155.1, 144.9, 141.5, 141.0, 133.9, 133.5, 128.3, 125.5, 67.6, 61.14, 61.09, 14.3, 14.1. IR (ATR) ν: 2959, 2941, 1547, 1455 cm^–1^. HRMS (ESI^+^) *m*/*z*: [M + Na]^+^ calcd for C_32_H_32_O_12_S_4_Na 759.0669; found 759.0669.

### Electrochemistry

All electrochemical experiments were performed in DMSO (for DNA and peptide synthesis, Merck, containing max 0.025% H_2_O) using 0.1 M tetrabutylammonium hexafluorophosphate (>98.0%, TCI) as supporting electrolyte. Due to a low conductivity, the three-electrode system was applied. As the working electrode glassy carbon electrode (diameter o̷ 1 mm), or Pt disk electrode (o̷ 1 mm) were used. As the reference electrode, a saturated calomel electrode (SCE) separated from the investigated sample by a salt bridge filled by the blank (DMSO electrolyte solution) was used and as the counter (auxiliary) electrode Pt wire was applied. All experiments were carried out in an undivided 20 mL cell, filled with 10 mL of the studied solution. Oxygen was removed from the solution by passing a stream of argon (Ar, 99.998%, Messer). The concentration of studied compounds is for each compound specified in ESI in the corresponding record descriptions. Scan rates used for CV experiments were 100, 200, 500, and 1000 mV·s^–1^. Linear sweep voltammetry on RDE was measured at a scan rate of 10 mV·s^–1^ with several rotation rates (100, 250, 500, and 1000 s^–1^). Before each measurement, the working electrodes (GC, or Pt) were mechanically cleaned using a polishing pad. All measurements were carried out using the computer-driven digital potentiostat PGSTAT101 (Autolab-Metrohm) controlled by software NOVA 1.11.

### X-ray Measurements

Generally: All single-crystals suitable for X-ray measurements were obtained by slow evaporation from a mixture of CH_2_Cl_2_/CHCl_3_/MeOH at room temperature.

#### Crystallographic Data for Compound **9**

*M* = 772.04 g·mol^–1^, triclinic system, space group *P*1̅, *a* = 11.4064 (3) Å, *b* = 11.5917 (3) Å, *c* = 16.3461 (4) Å, α = 82.3124 (8)°, β = 75.2557 (8)°, γ = 64.7618 (7)°, *Z* = 2, *V* = 1889.80 (8) Å^3^, *D*_*c*_ = 1.357 g.cm^–3^, μ(Cu-Kα) = 2.74 mm^–1^, crystal dimensions of 0.30 × 0.21 × 0.20 mm. Data were collected at 200 (2) K on a Bruker D8 Venture Photon CMOS diffractometer with Incoatec microfocus sealed tube Cu-Kα radiation. The structure was solved by charge flipping methods^20^ and anisotropically refined by full matrix least-squares on *F* squared using the CRYSTALS^21^ to final value *R* = 0.030 and *wR* = 0.080 using 6911 independent reflections (θ_max_ = 68.26°), 521 parameters and 55 restrains. The hydrogen atoms bonded to carbon atoms were placed in calculated positions refined with a riding constrains, while the hydrogen atoms bonded to oxygen atoms were located in residual electron density maps and refined with restrained geometry. The disordered solvent positions were found in difference electron density maps and refined with restrained geometry and ADPs. MCE^22^ was used for visualization of electron density maps. The occupancy of disordered solvent was constrained to full, resulting in final occupancy ratio of 895(3):105(3). The structure was deposited into Cambridge Structural Database under number CCDC 2424851.

#### Crystallographic Data for Compound **10a**

*M* = 608.86 g·mol^–1^, monoclinic system, space group *C*2/*c*, *a* = 33.940 (3) Å, *b* = 8.8814 (7) Å, *c* = 19.4661 (19) Å, β = 93.591 (4)°, *Z* = 8, *V* = 5856.3 (9) Å^3^, *D*_*c*_ = 1.381 g.cm^–3^, μ(Mo-Kα) = 0.36 mm^–1^, crystal dimensions of 0.70 × 0.09 × 0.04 mm. Data were collected at 180 (2) K on a Bruker D8 Venture Photon CMOS diffractometer with Incoatec microfocus sealed tube Mo-Kα radiation. The structure was solved by charge flipping methods^20^ and anisotropically refined by full matrix least-squares on *F* squared using the CRYSTALS^21^ to final value R = 0.085 and *wR* = 0.242 using 5975 independent reflections (θ_max_ = 26.37°), 372 parameters and 7 restrains. The hydrogen atoms bonded to carbon atoms were placed in calculated positions refined with a riding constrains. The disordered sulfur bridge positions were found in difference electron density maps and refined with restrained ADPs. MCE^22^ was used for visualization of electron density maps. The occupancy of disordered sulfur was constrained to full, resulting in final occupancy ratio of 792(17):208(17). The structure was deposited into Cambridge Structural Database under number CCDC 2424850.

#### Crystallographic Data for Compound **11**

*M* = 821.79 g·mol^–1^, triclinic system, space group *P*2_1_/*n*, *a* = 13.1583(5) Å, *b* = 11.8585(5) Å, *c* = 24.1797(9) Å, β = 95.5330(18)°, *Z* = 4, *V* = 3755.4(3) Å^3^, *D*_*c*_ = 1.453 g·cm^–3^, μ(Cu-Kα) = 4.15 mm^–1^, crystal dimensions of 0.04 × 0.20 × 0.25 mm. Data were collected at 180 (2) K on a Bruker D8 Venture Photon II 7 diffractometer with Incoatec microfocus sealed tube Cu–Kα radiation. Due to the small crystal size the total exposure time was 58 h. Data reduction, scaling and absorption correction were performed using Apex4.^23^ The structure was solved by direct methods^24^ and refined anisotropically by full-matrix least-squares on *F*^2^ in the CRYSTALS programs^21^ to final values of R = 0.065 and *wR* = 0.1998 using 7113 independent reflections (θ_max_ = 70.171°), 559 parameters and 30 restraints. Hydrogen atoms bonded to carbon atoms were placed in calculated positions and refined with riding constrains. The disordered solvent positions were found in difference electron density maps and refined with restrained geometry and ADPs. MCE^22^ was used for visualization of the electron density maps. One part of the main molecule exhibited disorder and was modeled in two positions. The occupancy of the disordered parts was initially refined, then rounded and fixed at 0.6 and 0.4 during the final stages of the refinement. The occupancy of the disordered dichloromethane was initially refined, then rounded and fixed, which resulted in a complete molecule. The structure was deposited into Cambridge Structural Database under number CCDC 2422748.

## Data Availability

The data underlying this study are available in the published article and its online Supporting Information.

## Supporting Information Available

Copies of ^1^H, ^13^C NMR, IR spectra, and HRMS of new compounds, X-ray crystallographic data for compound, and electrochemical measurements (PDF)
