# Supplementary material for: Synthesis of Thiacalix[4]arene Skeleton by the Conjugate Addition of Benzoquinone
Source: J Org Chem. 2025 Apr 30;90(18):6294–303. doi: 10.1021/acs.joc.5c00405 (PMC12070407; doi:10.1021/acs.joc.5c00405)
Supplement: Supplementary file 1 — jo5c00405_si_001.pdf [file jo5c00405_si_001.pdf]

Supporting Information

Synthesis of Thiacalix[4]arene Skeleton by the Conjugate Addition of Benzoquinone

Kamil Mamleev,[a] Nicolai Nikishkin,[a] Jan Čejka,[b] Václav Eigner,[b] Karolína Salvadori,[c,d] Hana Dvořáková[e] and Pavel Lhoták[a,*]

[a] Department of Organic Chemistry, University of Chemistry and Technology Prague (UCTP), Technická 5, 166 28, Prague 6, Czech Republic. E-mail: lhotakp@vscht.cz

[b] Department of Solid State Chemistry, UCTP, Technická 5, 166 28, Prague 6, Czech Republic.

[c] J. Heyrovský Institute of Physical Chemistry, Academy of Sciences CR, Dolejškova 2155/3, 182 00, Prague 8, Czech Republic

[d] Department of Analytical Chemistry, UCTP, Technická 5, 166 28, Prague 6, Czech Republic

[e] Laboratory of NMR spectroscopy, UCTP, Technická 5, 166 28, Prague 6, Czech Republic.

Table of Contents

1. Synthetic procedures 2
2. Spectral characterization of compounds 5
3. Dynamic NMR experiments 30
4. Electrochemical measurements 36
5. X-ray data 49
6. Literature 57

1. Synthetic procedures

General Information.

All chemicals were purchased from commercial sources and used without further purification. THF and CH_3CN were dried using a column solvent purification system PureSolv MD7 (Inert). Melting points were measured on a Heiztisch Mikroskop Polytherm A (Wagner & Munz), and they are not corrected. The ^{1}H and $^{13}C\{^{1}H\}$ NMR spectra were recorded on an Agilent 400-MR DDR2 and JEOL-ECZL400G (^{1}H: 400 MHz, ^{13}C: 100 MHz). The ^{1}H VT NMR experiments were carried out on a Bruker Avance III™ 500 MHz. The chemical shifts (δ) are reported in parts per million (ppm) and were referenced to the residual peaks of the solvent or TMS as an internal standard; the coupling constants (J) are expressed in Hz. All the NMR data were processed and displayed using MestReNova software. The FTIR analysis was performed on a Nicolet iS50 spectrometer (Thermo-Nicolet, USA) connected with a GladiATR diamond placed outside the conventional sample compartment, equipped with DTGS KBr detector. Reflectance data were acquired with the following parameters - spectral range: 4000-400 cm^{-1}, resolution: 4 cm^{-1}, number of spectra accumulations: 64, apodisation: Happ-Genzel. The spectra were collected and processed by Omnic 9 (Thermo-Nicolet Instruments Co., USA) including baseline correction and Savitzky-Golay smoothing filter (set to the number of 11 points used in the algorithm). The resulting spectrum is the average of three independent measurements. ESI HRMS spectra were measured on a LC-MS LTQ-Orbitrap Velos (Thermo) spectrometer. Substance purities and the reaction progress were monitored by thin layer chromatography (TLC) using silica gel 60 F_{254} on aluminium-backed sheets (Merck) and analysed at 254 and/or 365 nm. Radial chromatography was carried out on Chromatotron (Harrison Research) connected with a lab pump RHSY2 (Fluid Metering). Self-prepared glass discs were covered by silica gel 60 PF_{254} containing $CaSO_4$ (Merck). Self-prepared glass plates for preparative TLC (20 x 20 cm) were covered by silica gel 60 PF_{254} containing $CaSO_4$ (Merck).

Synthesis of benzene-1,3-dithiol

This compound was prepared by modification of the described procedure.[1]

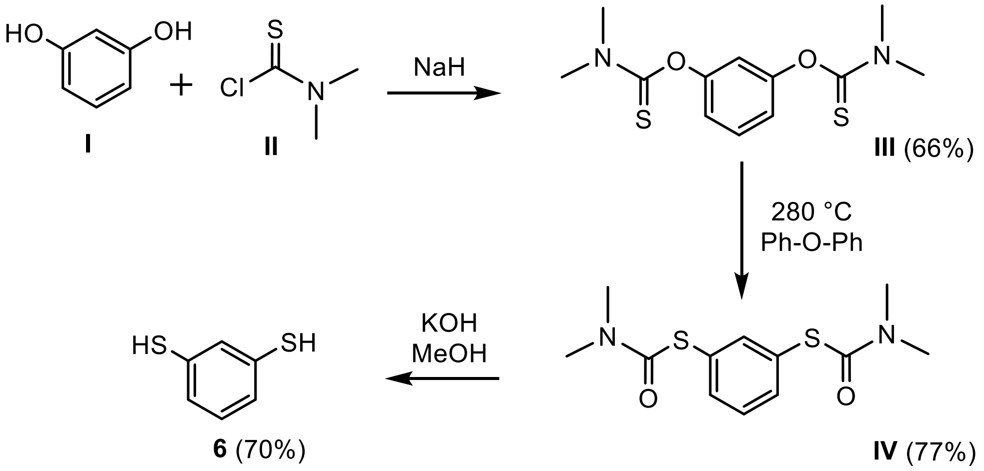

***O,O'*-(1,3-phenylene) bis(dimethylcarbamothioate) (III)**. Resorcinol **I** (10 g, 90.20 mmol) was dissolved in 200 mL of dry DMF. Sodium hydride (9.2 g, 230.00 mmol) and dimethylthiocarbamoyl chloride **II** (25 g, 202.27 mmol) were added and the reaction mixture was stirred and heated (heating block) at 70 °C for 3 h. KOH (5%, 500 mL) was added and the crude product was extracted with benzene (3 x 120 mL). Water (30 mL) was added and the crude product was extracted with chloroform (3 x 30 mL), the organic phase was then washed with 5% KOH and with water, dried over $MgSO_4$ and separated by vacuum dry-column chromatography on silica gel (eluent = DCM). **Intermediate III** was obtained in 66% (17.08 g) yield as a yellowish oil.

^{1}H NMR ($CDCl_3$, 400 MHz, 298 K) δ (ppm): 7.41 – 7.36 (m, 1H), 6.97 (dd, J = 8.2, 2.3 Hz, 2H), 6.84 (t, J = 2.2 Hz, 1H), 3.44 (s, 6H), 3.32 (s, 6H). ^{13}C NMR ($CDCl_3$, 100 MHz, 298 K) δ (ppm): 187.4, 154.3, 129.2, 120.6, 118.2, 43.4, 39.0.

***S,S'*-(1,3-phenylene) bis(dimethylcarbamothioate) (IV)**. Intermediate **III** (17,08 g, 60.06 mmol) was dissolved in 90 mL of dry diphenyl ether and the reaction mixture was stirred and heated (heating block) at 300 °C for 3 h under argon atmosphere. The cooled product was separated by vacuum dry-column chromatography on silica gel (eluent = cyclohexane to isolate diphenyl ether and eluent = DCM to isolate product). **Intermediate IV** was obtained in 77% (13.16 g) yield as a yellowish solid.

^{1}H NMR ($CDCl_3$, 400 MHz, 298 K) δ (ppm): 7.64 (t, *J* = 1.8 Hz, 1H), 7.55 – 7.48 (m, 2H), 7.44 – 7.37 (m, 1H), 3.08 (s, 6H), 3.02 (s, 6H). ^{13}C NMR ($CDCl_3$, 100 MHz, 298 K) δ (ppm): 166.6, 142.3, 136.5, 129.6, 129.3, 53.6, 37.0.

Benzene-1,3-dithiol (6). Intermediate **IV** (9,06 g, 31.86 mmol) was dissolved in 150 mL of dry MeOH and KOH (35 g, 0,62 mol) was added and the reaction mixture was stirred and heated (heating block) at reflux for 4 h under argon atmosphere. The reaction mixture was allowed to cool to rt before being poured into 1M HCl (100 mL) in an ice bath. 5M HCl was added to create acid conditions. The crude product was extracted with diethyl ether (5 x 40 mL), the organic phase was then washed with water and dried over $MgSO_4$. The product was isolated by vacuum distillation. Compound **6** was obtained in 70% (3.16 g) yield as yellowish liquid. All the data are in agreement with the literature.[2]

^{1}H NMR ($CDCl_3$, 400 MHz, 298 K) δ (ppm): 7.20 – 7.18 (m, 1H), 7.12 – 7.02 (m, 3H), 3.43 (s, 2H).

2. Spectral characterization of compounds

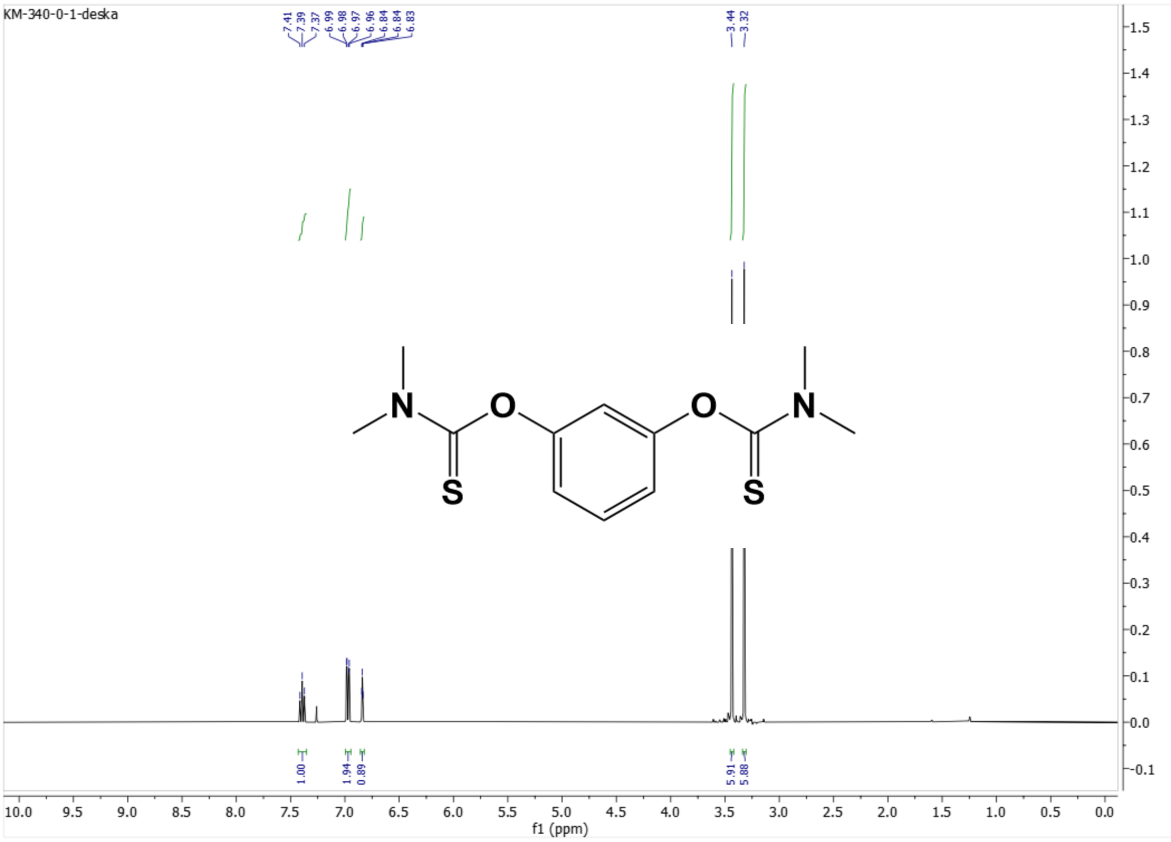

Figure S1: ^{1}H NMR of intermediate **III** ($CDCl_3$, 400 MHz).

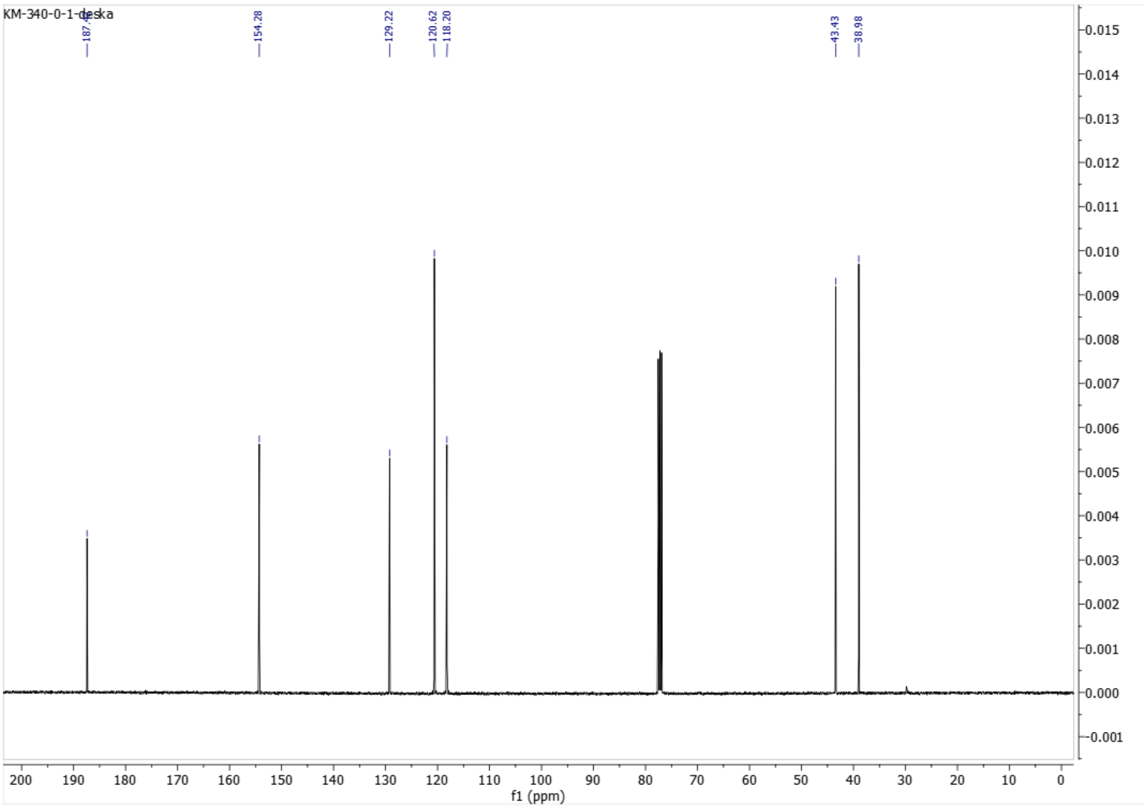

Figure S2: ^{13}C NMR of intermediate **III** ($CDCl_3$, 100 MHz).

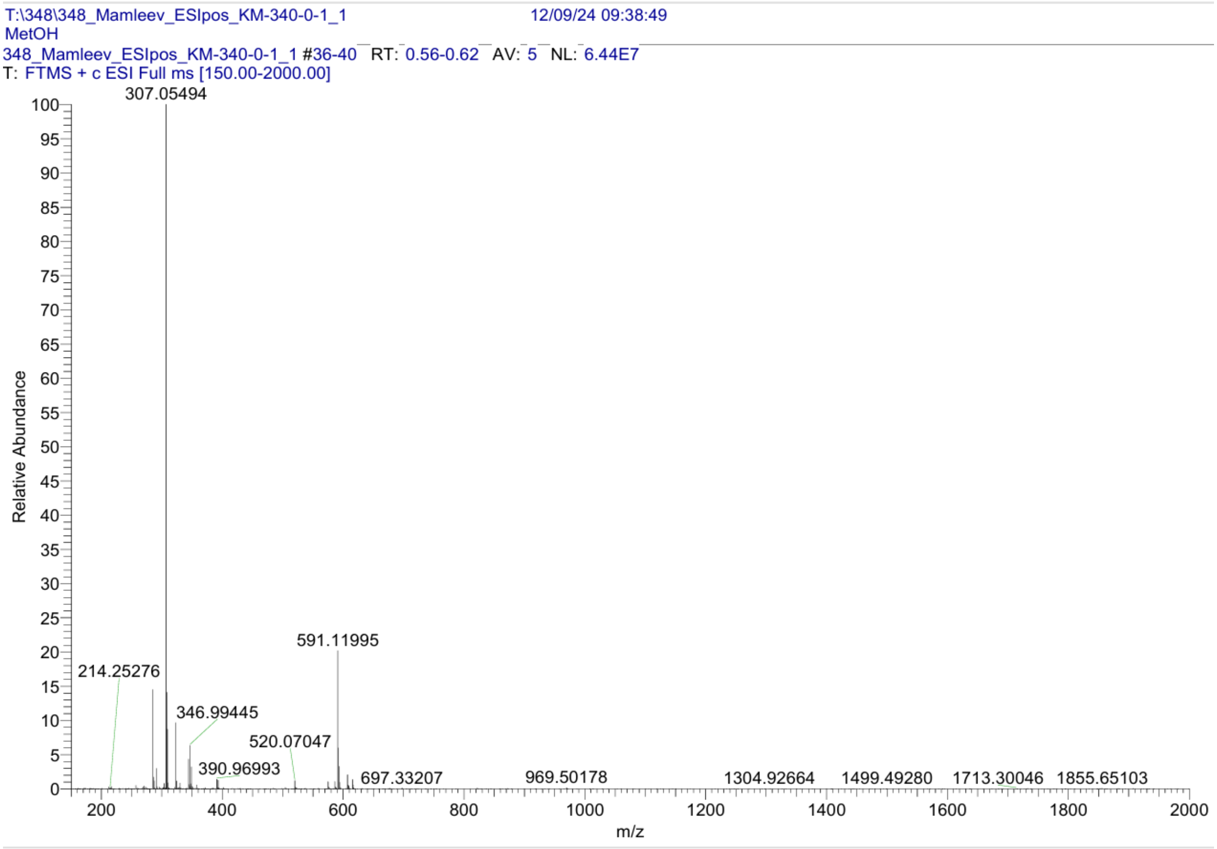

Figure S3: Full spectrum HRMS of intermediate **III** (ESI$^+$).

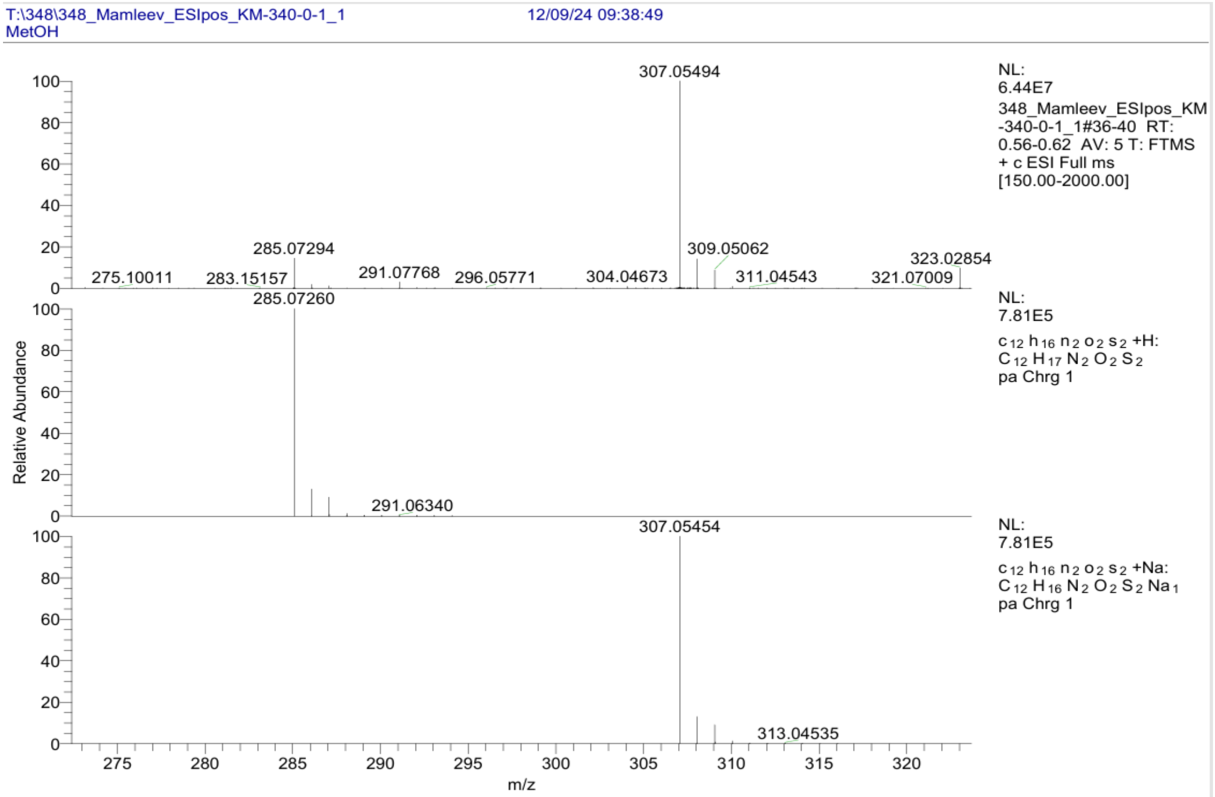

Figure S4: HRMS of intermediate **III** (ESI$^+$).

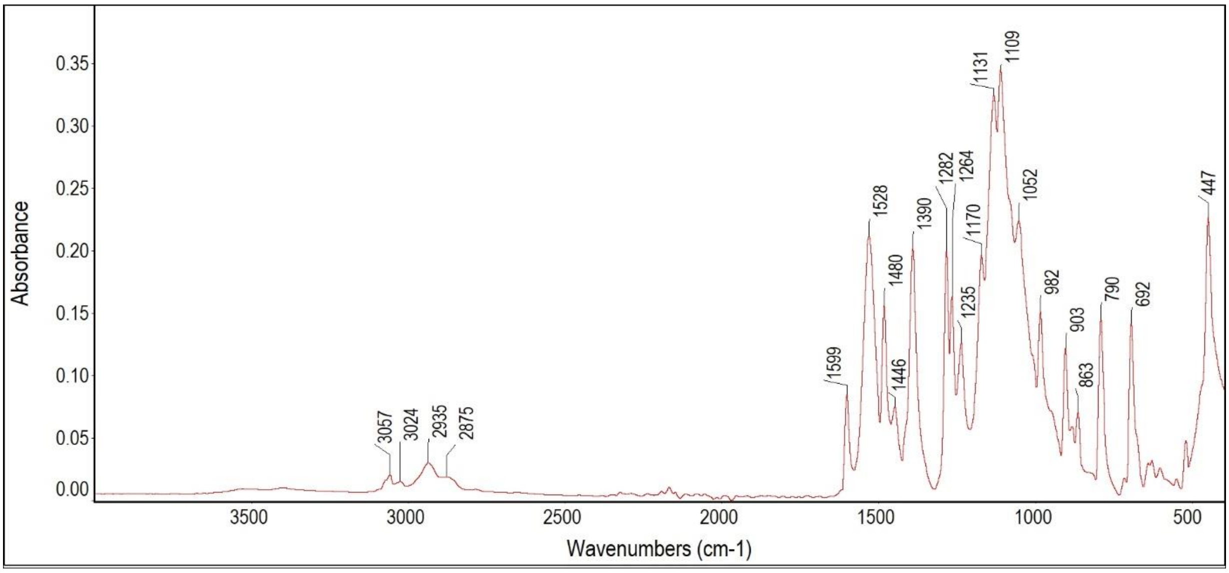

Figure S5: IR spectrum (ATR) of intermediate **III**.

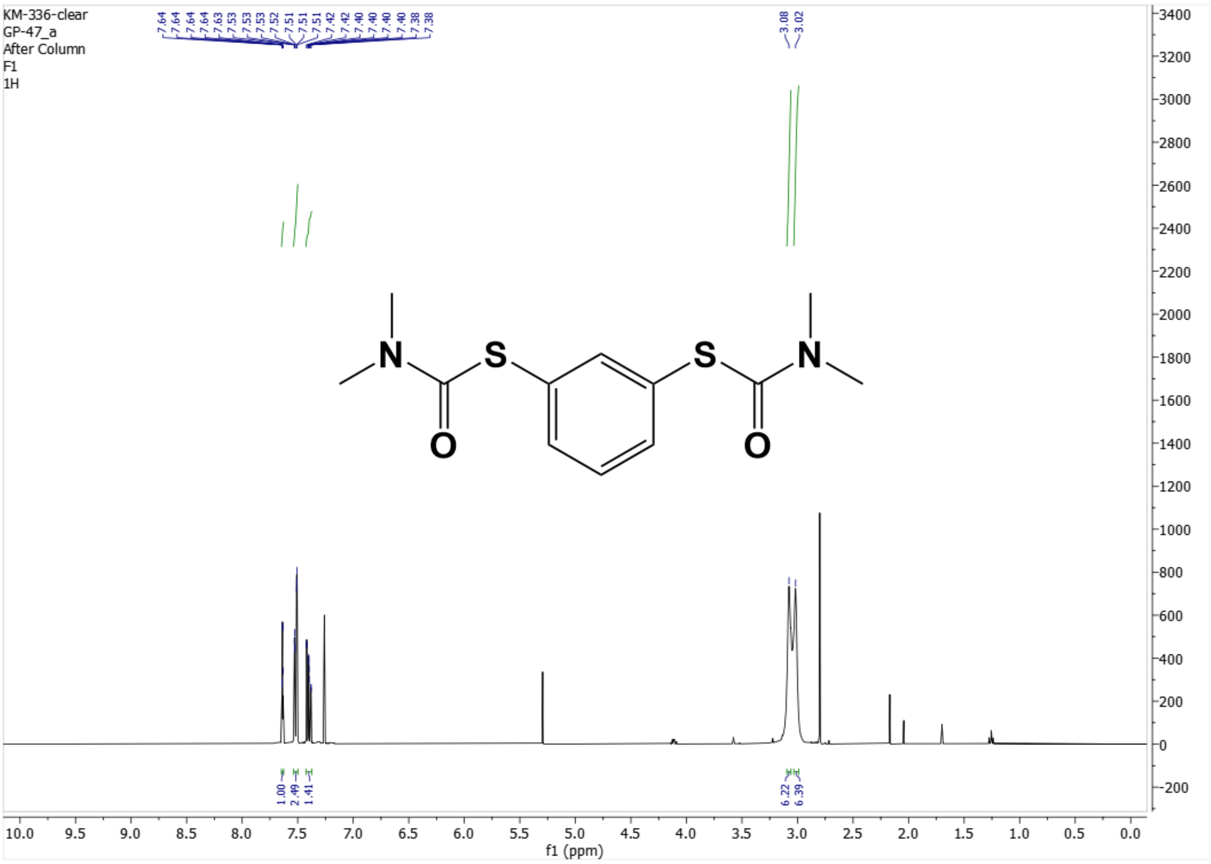

Figure S6: ^{1}H NMR of intermediate **IV** ($CDCl_3$, 400 MHz).

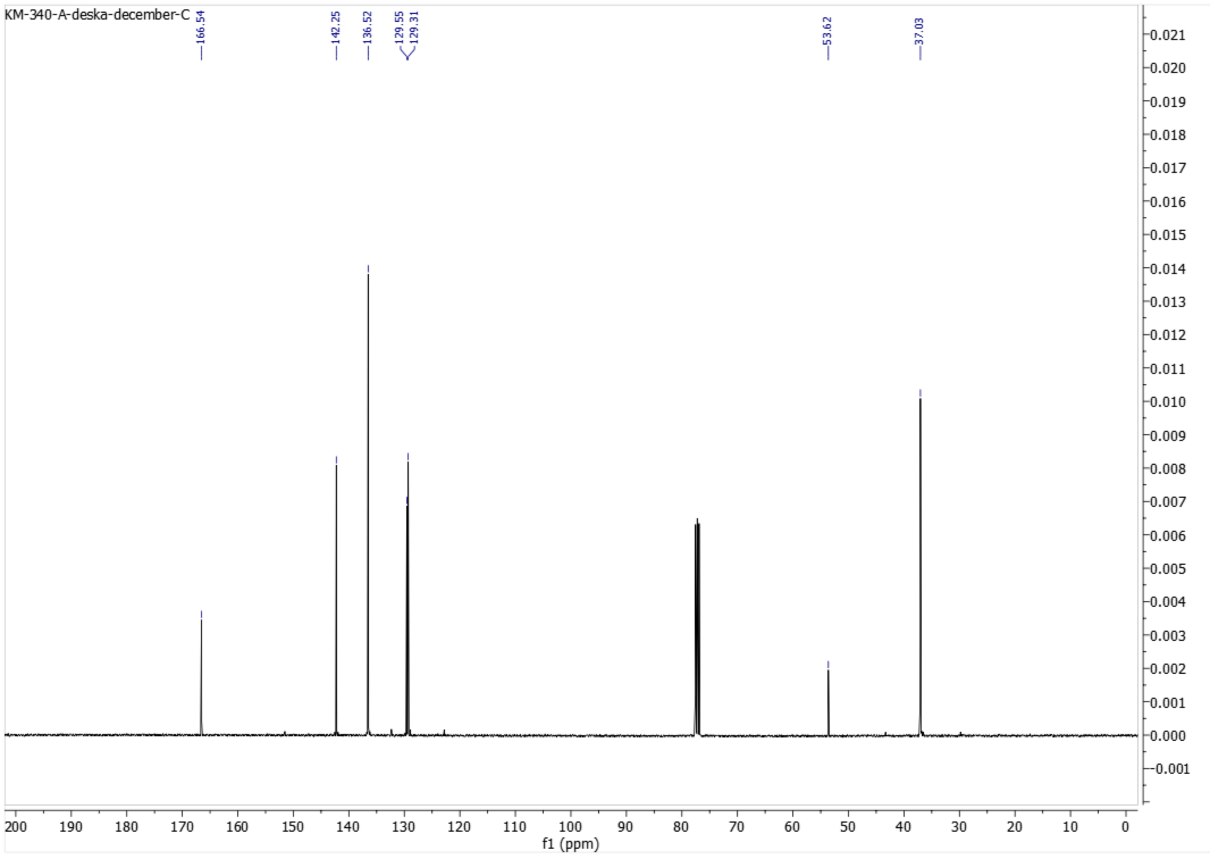

Figure S7: ^{13}C NMR of intermediate **IV** ($CDCl_3$, 100 MHz).

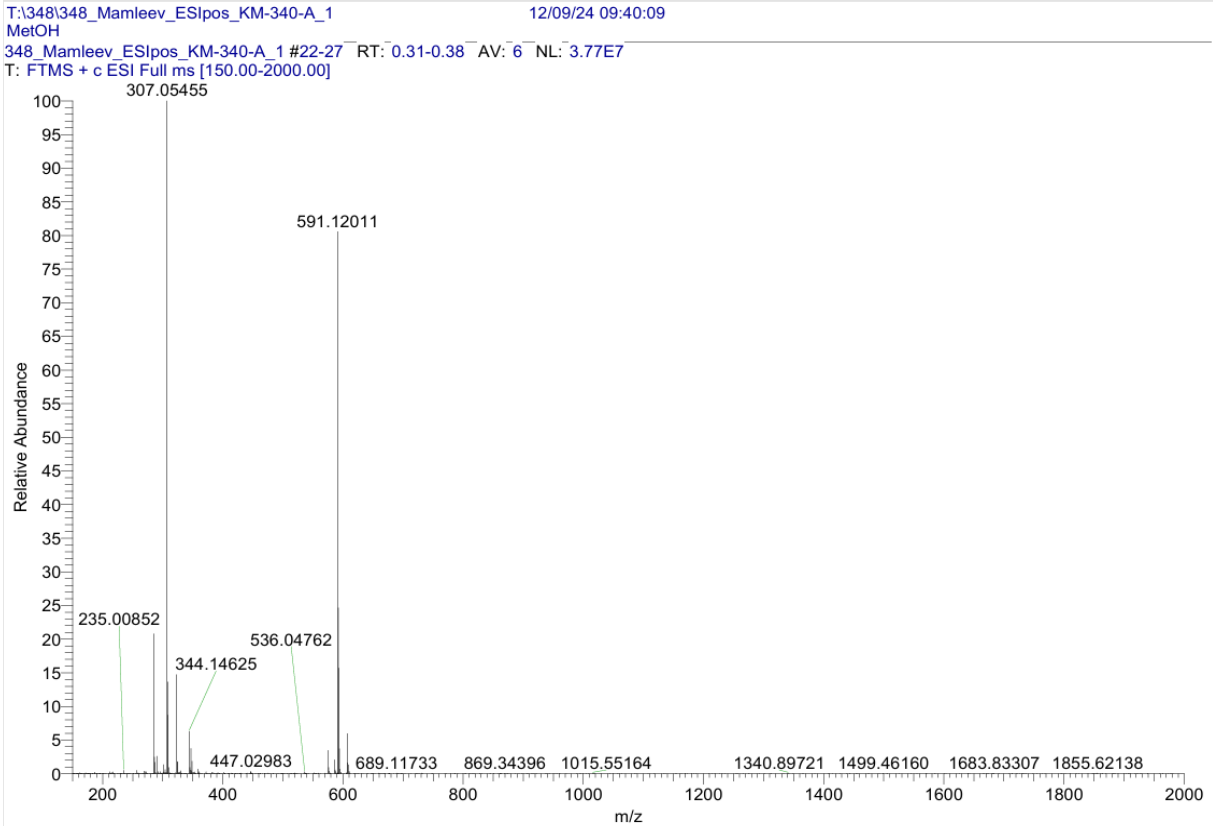

Figure S8: Full spectrum HRMS of intermediate **IV** (ESI$^+$).

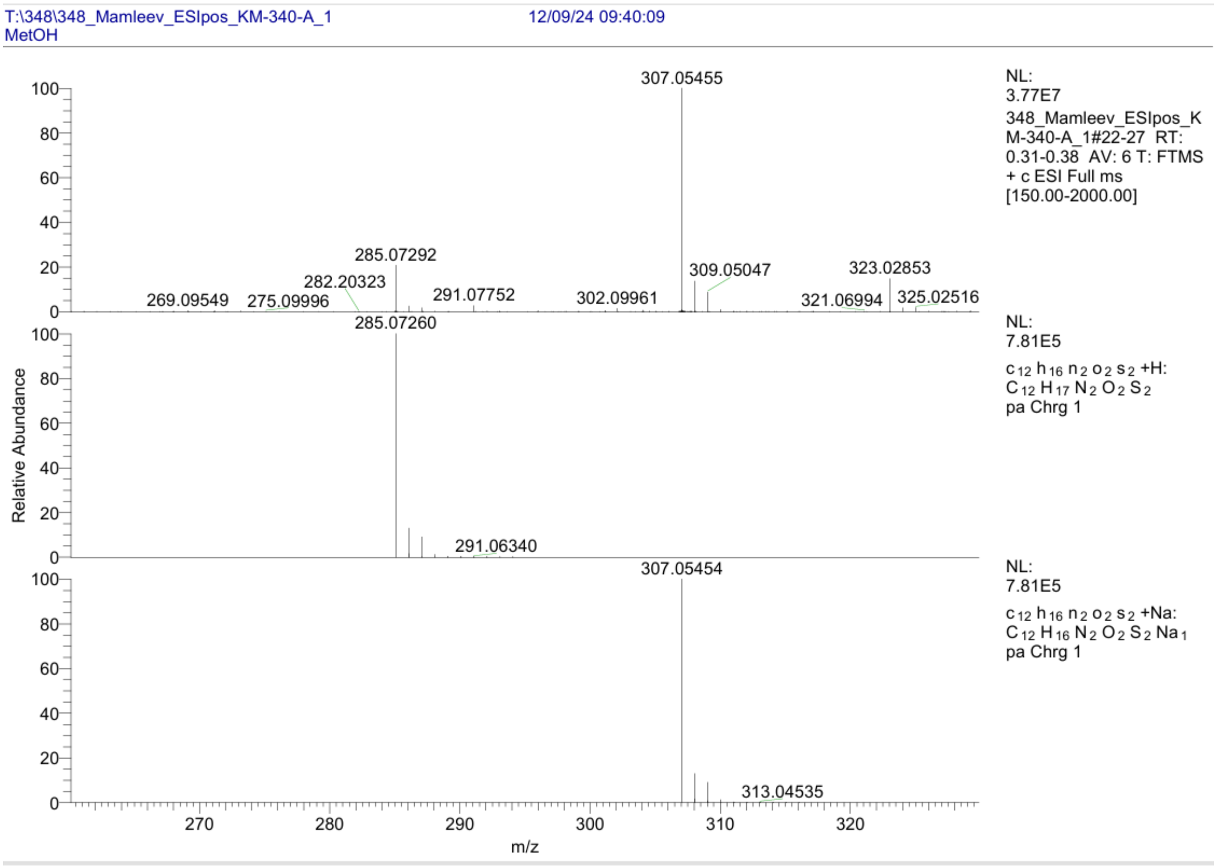

Figure S9: HRMS of intermediate **IV** (ESI$^+$).

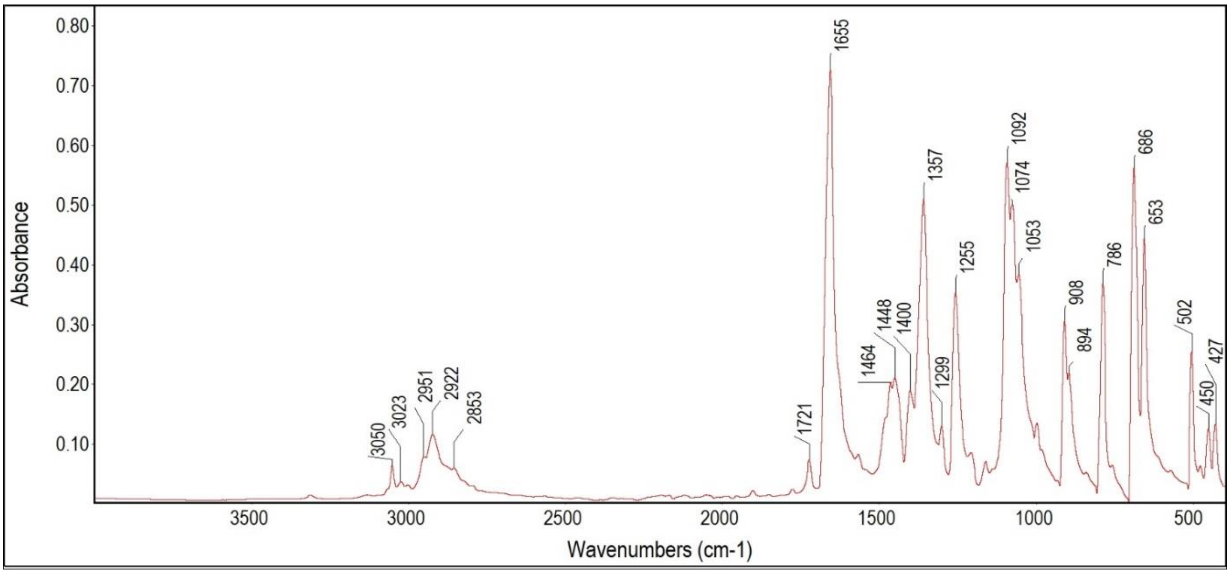

Figure S10: IR spectrum (ATR) of intermediate **IV**.

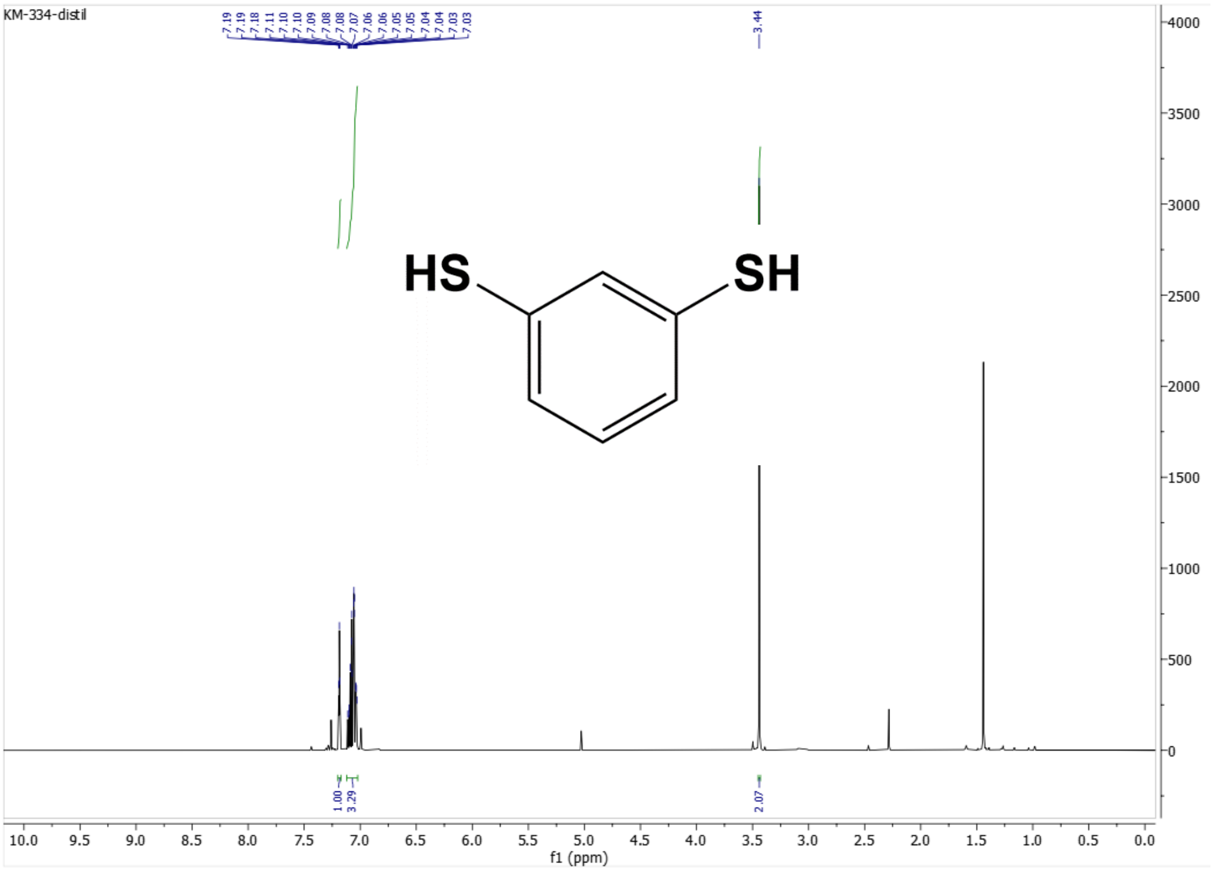

Figure S11: ^{1}H NMR of benzene-1,3-dithiol **6** ($CDCl_3$, 400 MHz).

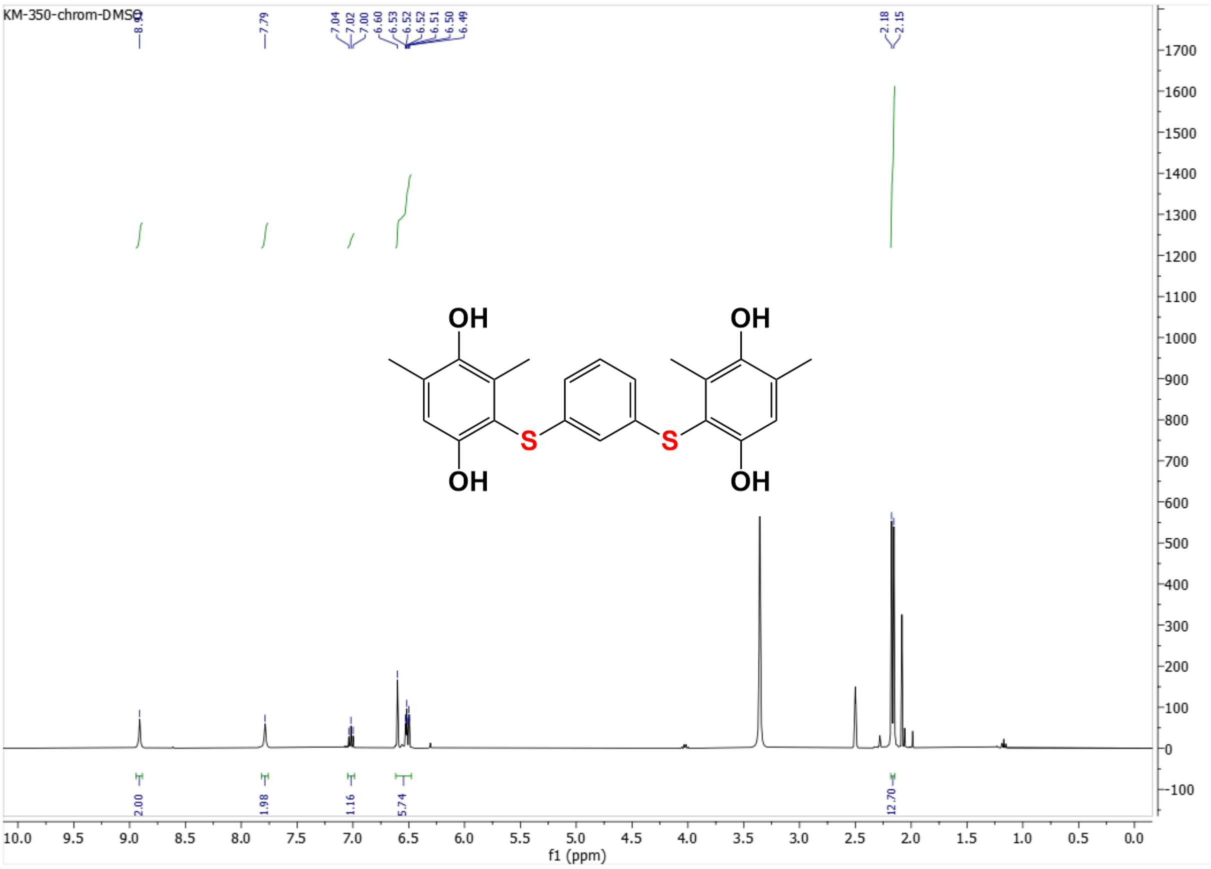

Figure S12: ^{1}H NMR of compound **7** (DMSO-d_6, 400 MHz).

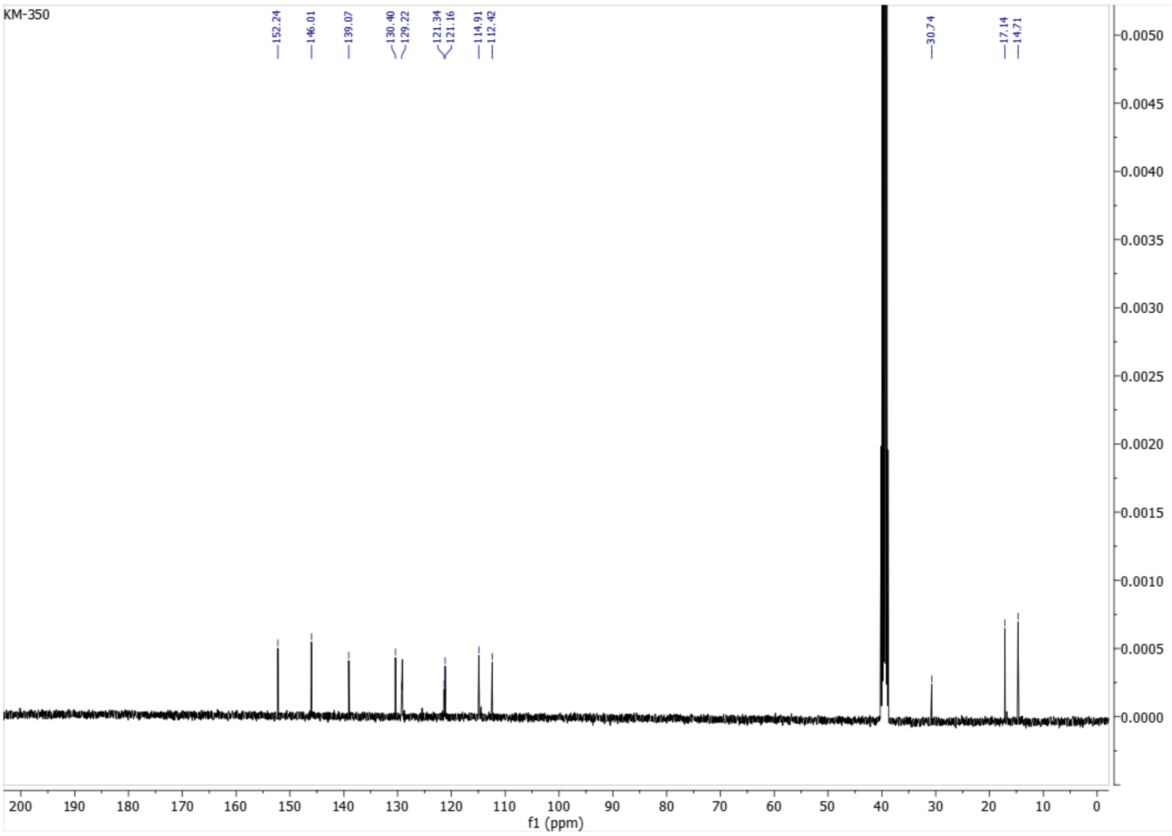

Figure S13: ^{13}C NMR of compound **7** (DMSO-d_6, 100 MHz).

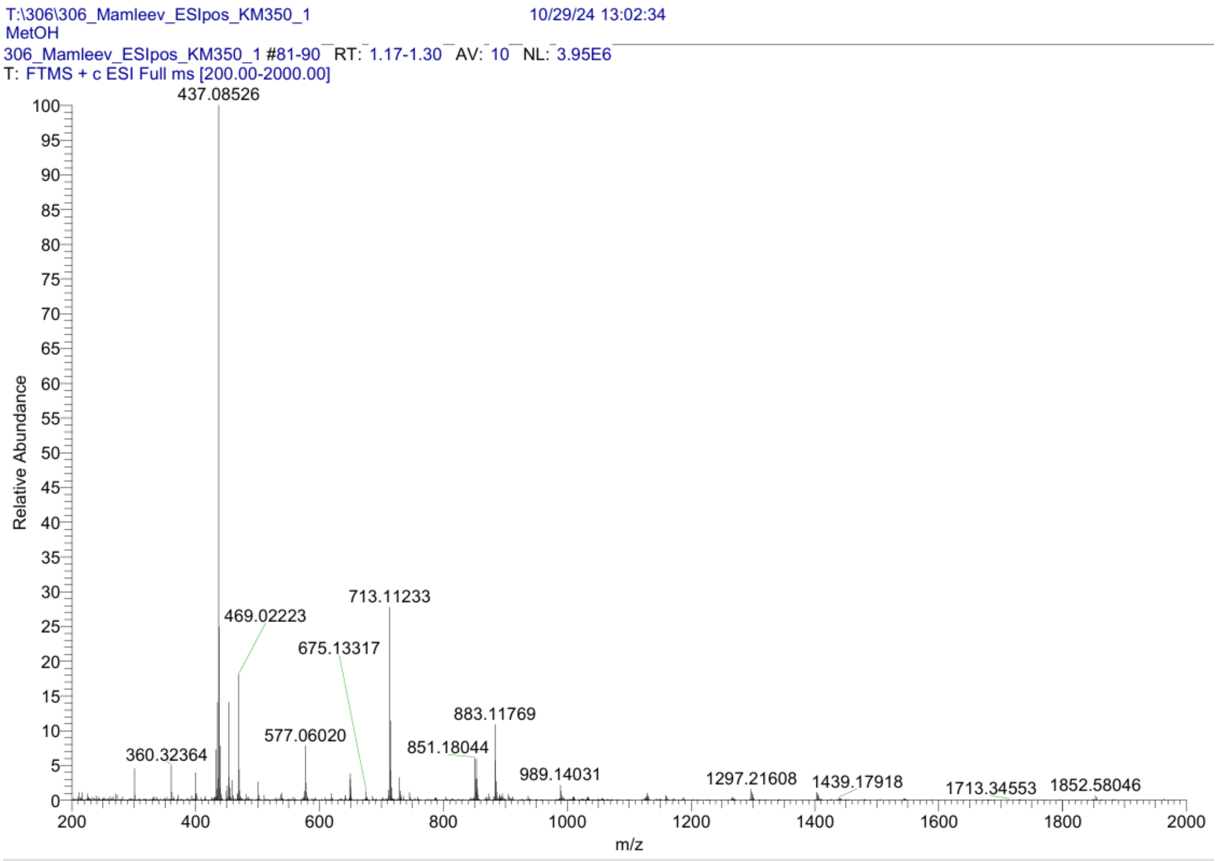

Figure S14: Full spectrum HRMS of compound **7** (ESI+).

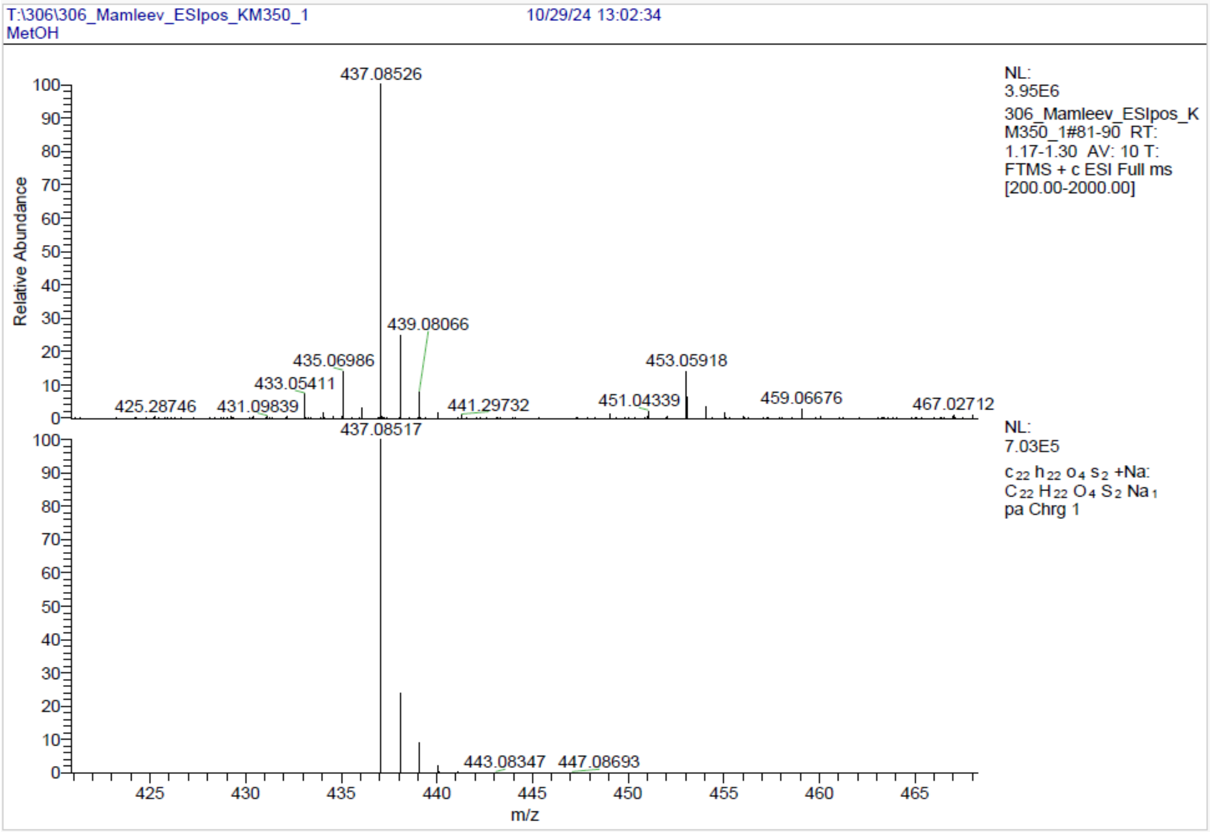

Figure S15: HRMS of compound **7** (ESI+).

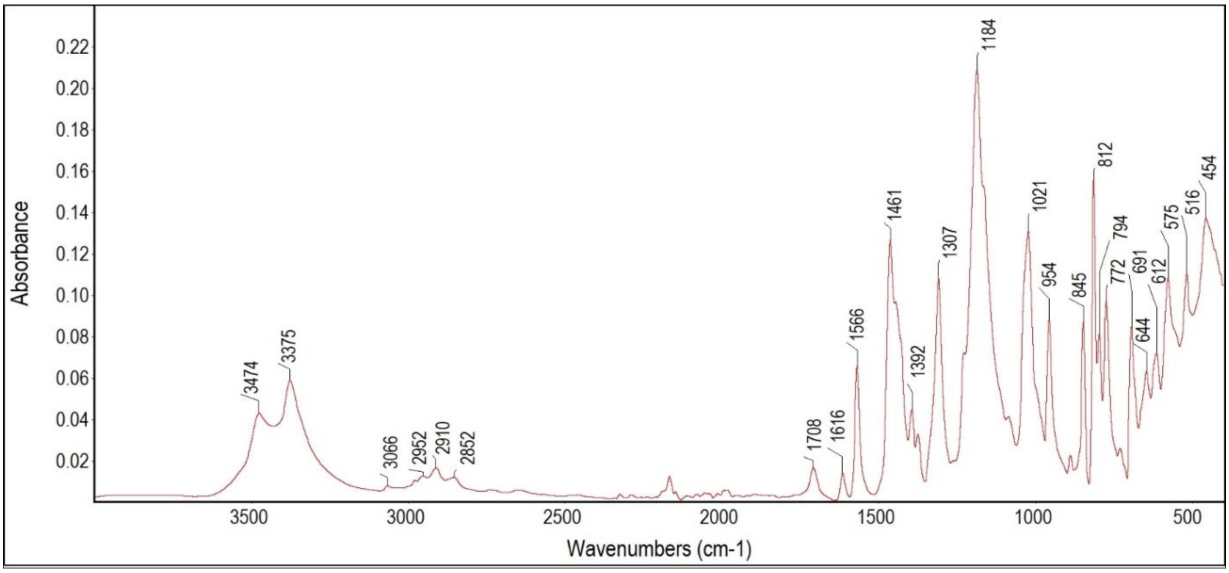

Figure S16: IR spectrum (ATR) of compound **7**.

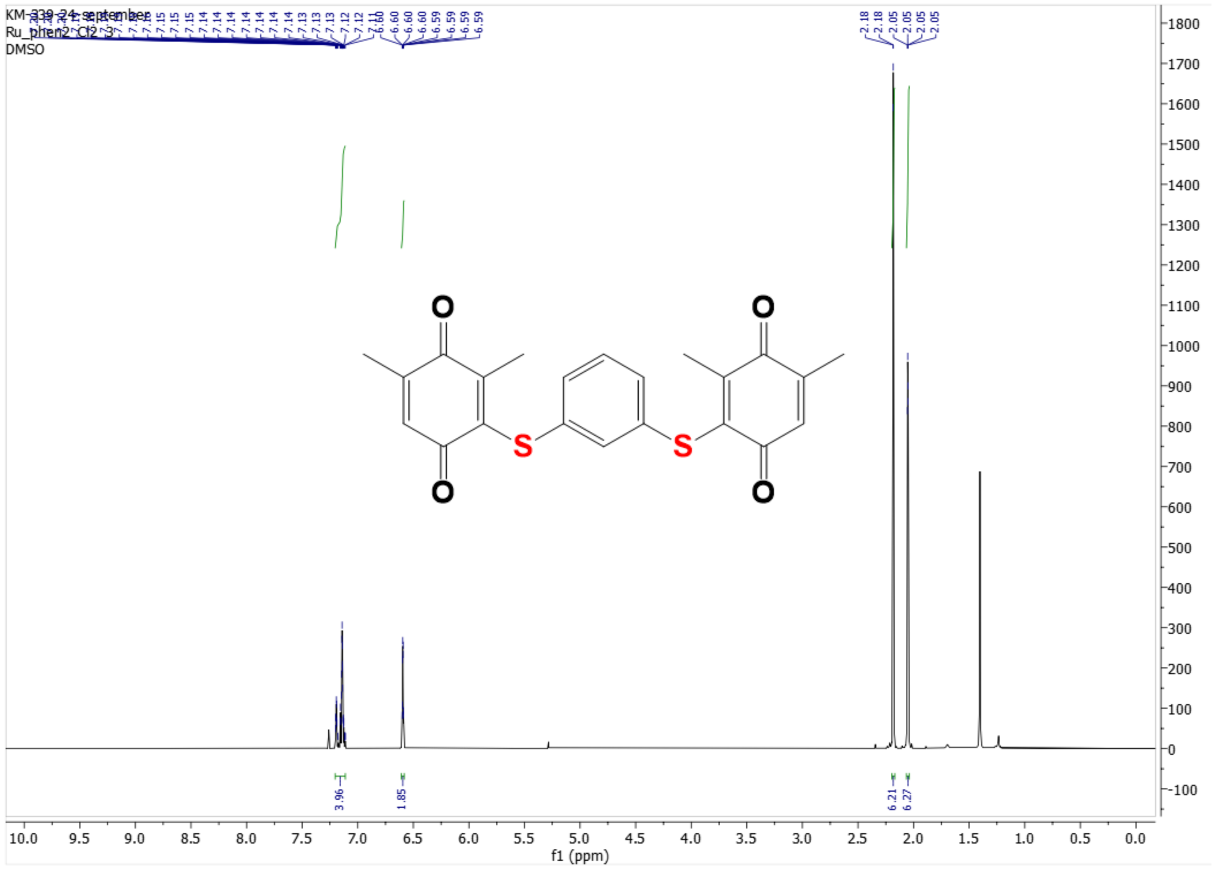

Figure S17: ^{1}H NMR of compound **8** ($CDCl_3$, 400 MHz).

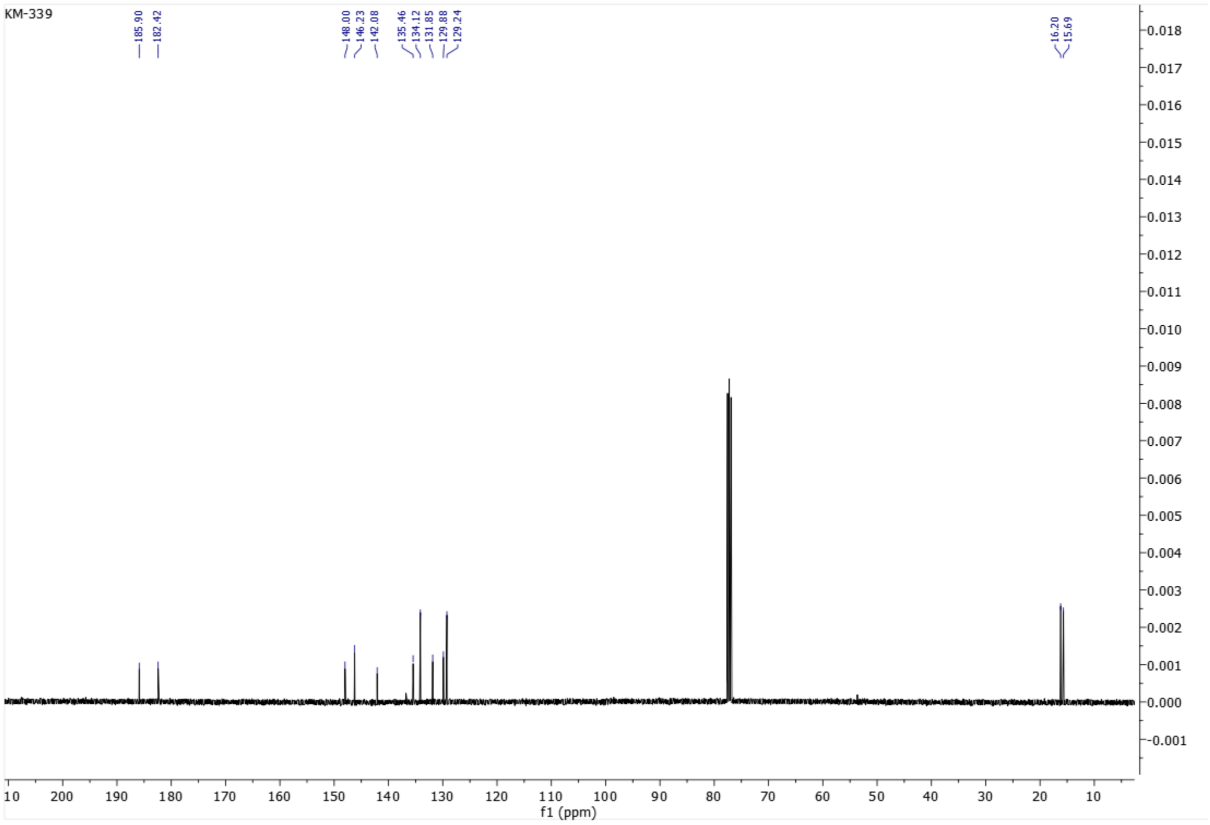

Figure S18: ^{13}C NMR of compound **8** ($CDCl_3$, 100 MHz).

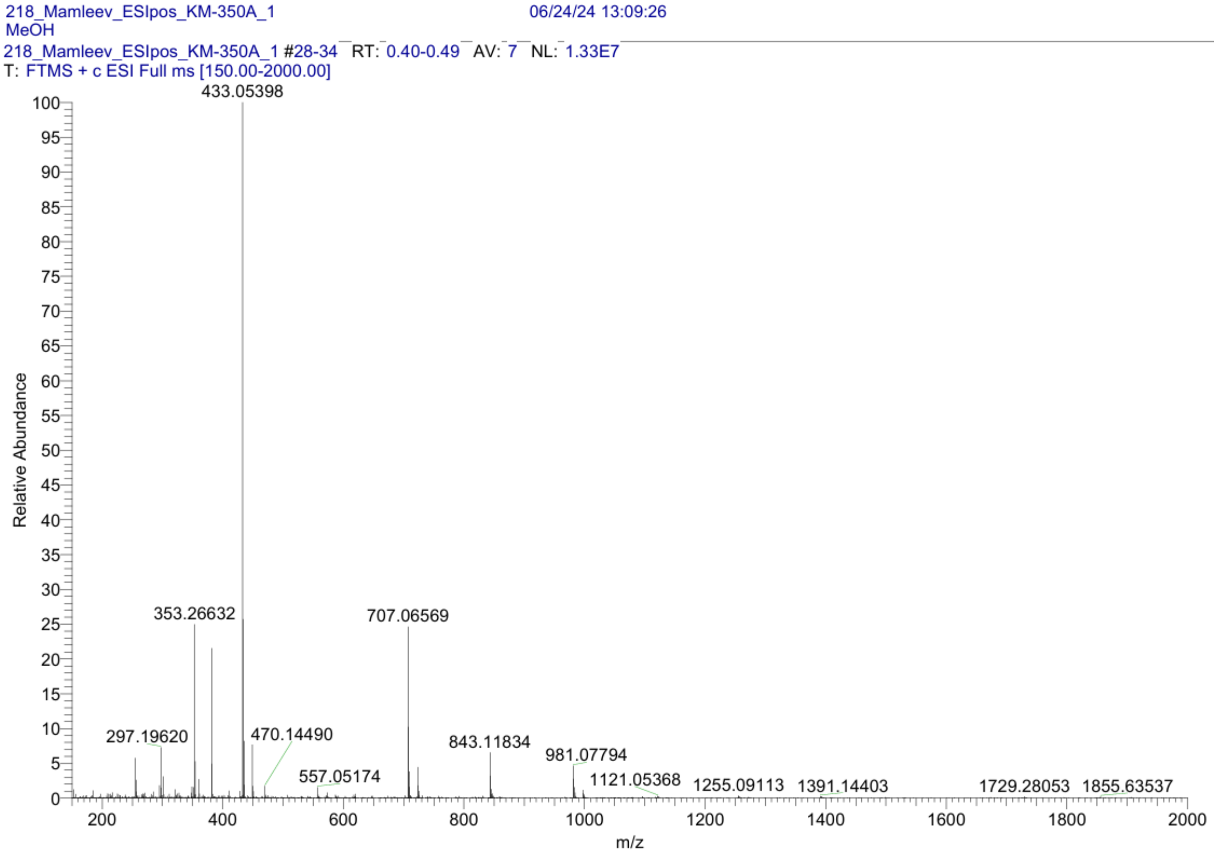

Figure S19: Full spectrum HRMS of compound **8** (ESI^+).

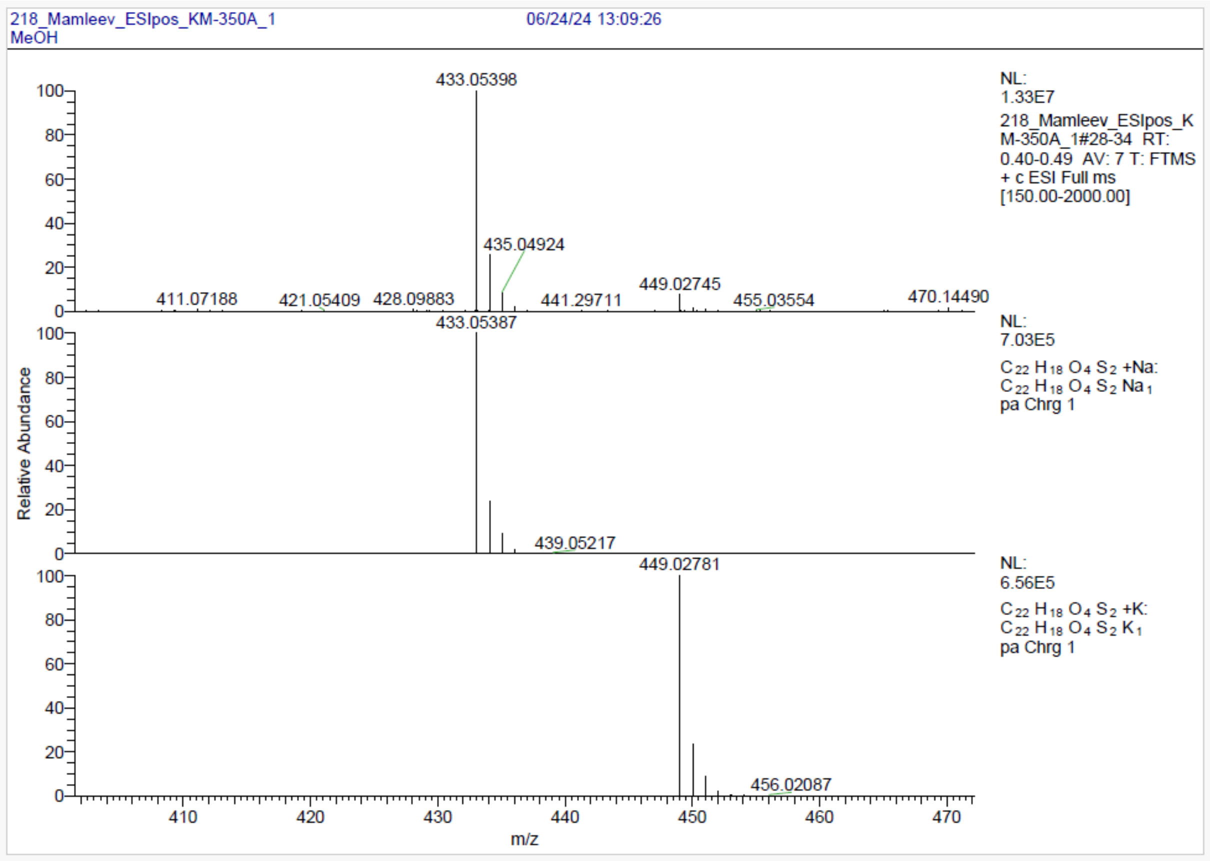

Figure S20: HRMS of compound **8** (ESI$^+$).

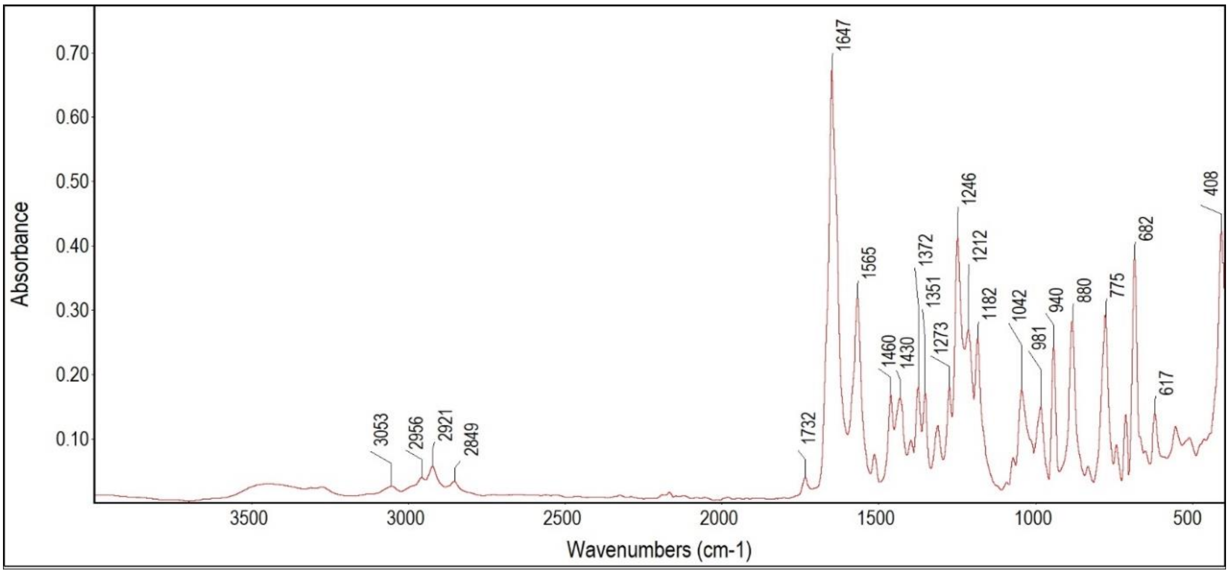

Figure S21: IR spectrum (ATR) of compound **8**.

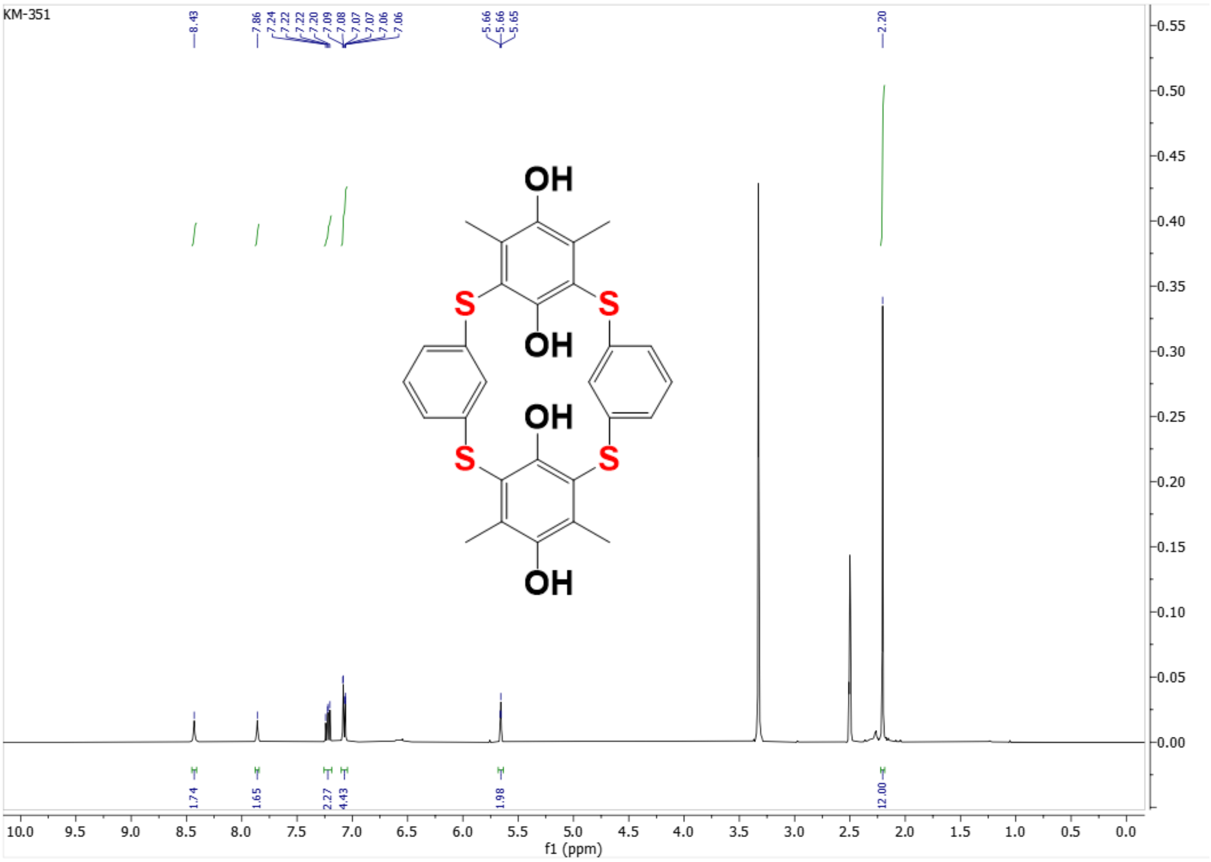

Figure S22: ^{1}H NMR of compound **9** (DMSO-d_6, 400 MHz).

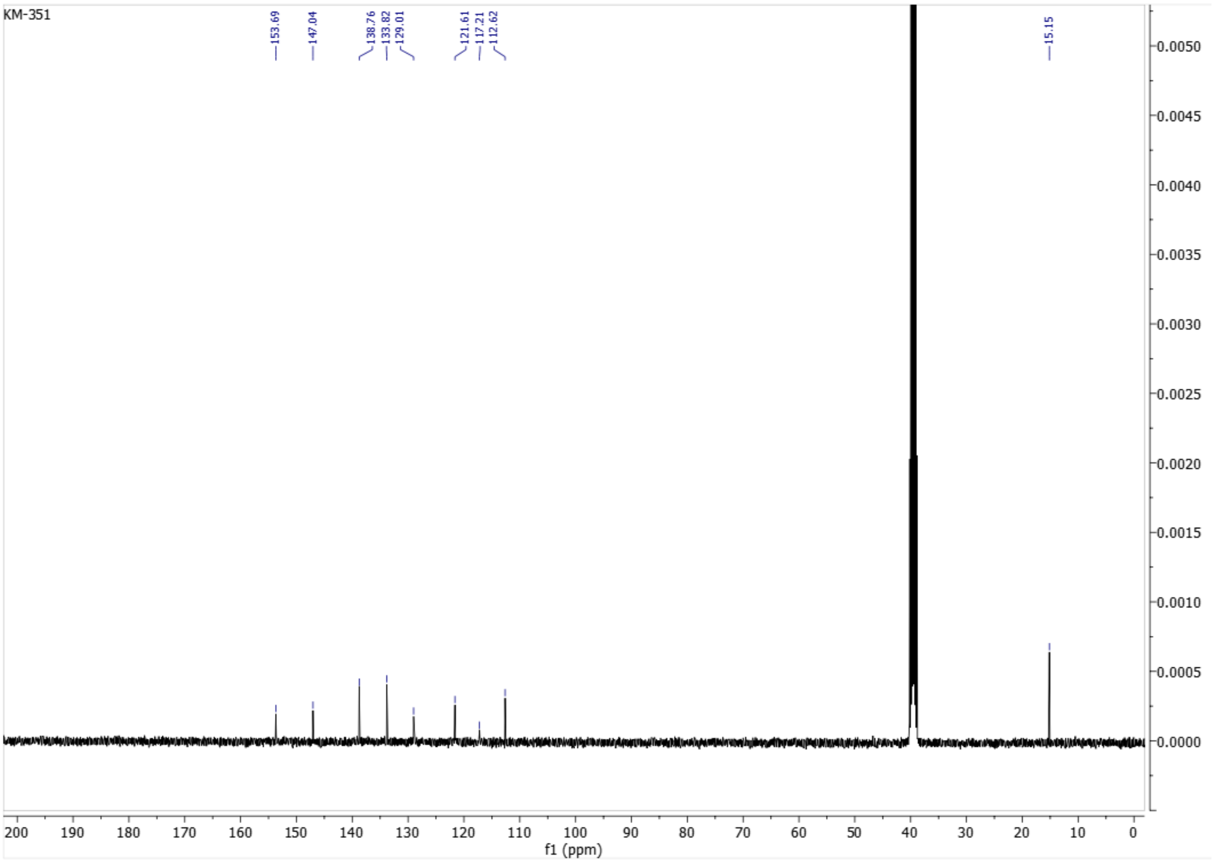

Figure S23: ^{13}C NMR of compound **9** (DMSO-d_6, 100 MHz).

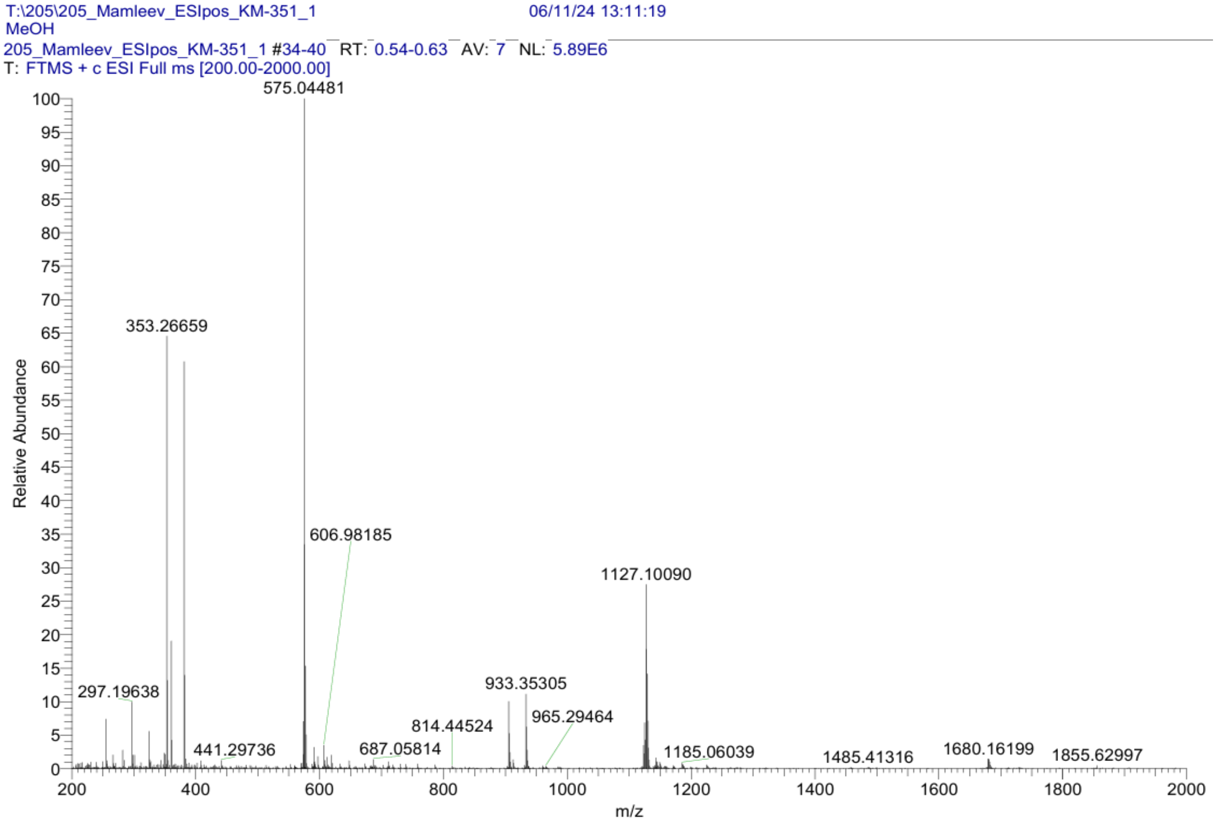

Figure S24: Full spectrum HRMS of compound **9** (ESI$^+$).

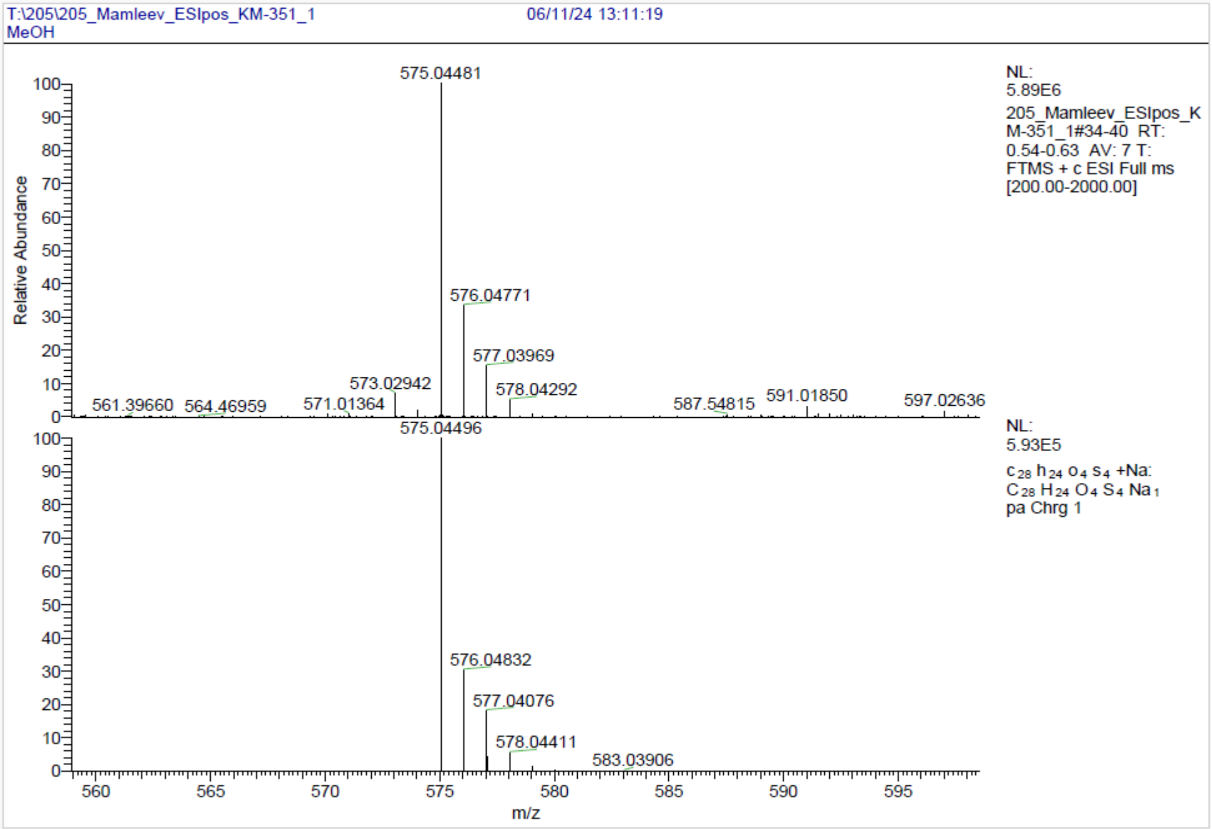

Figure S25: HRMS of compound **9** (ESI^{+}).

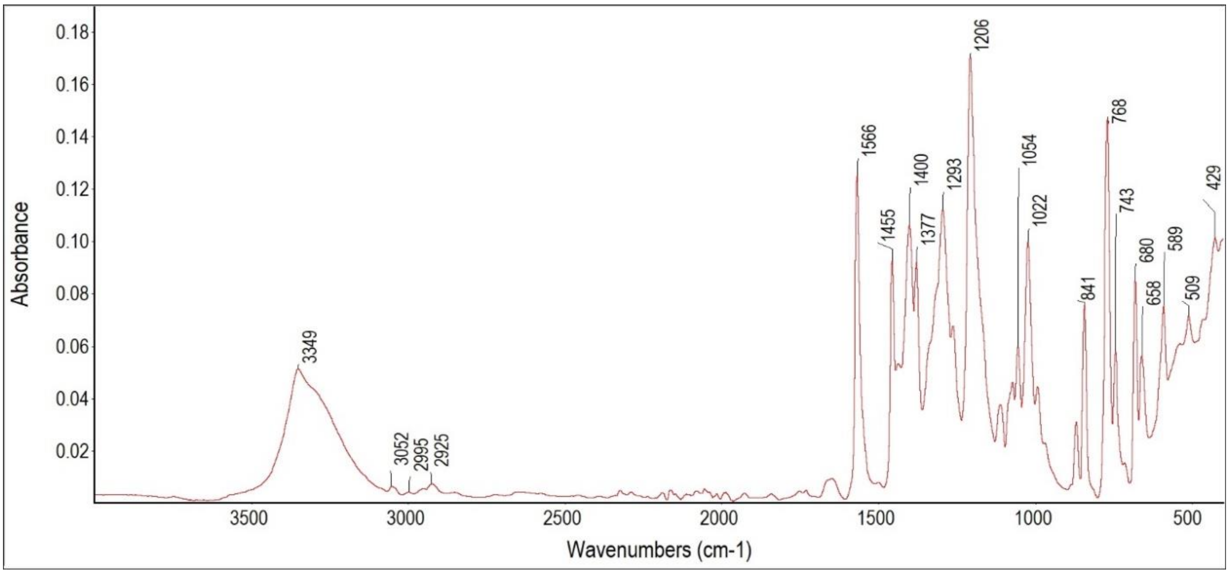

Figure S26: IR spectrum (ATR) of compound **9**.

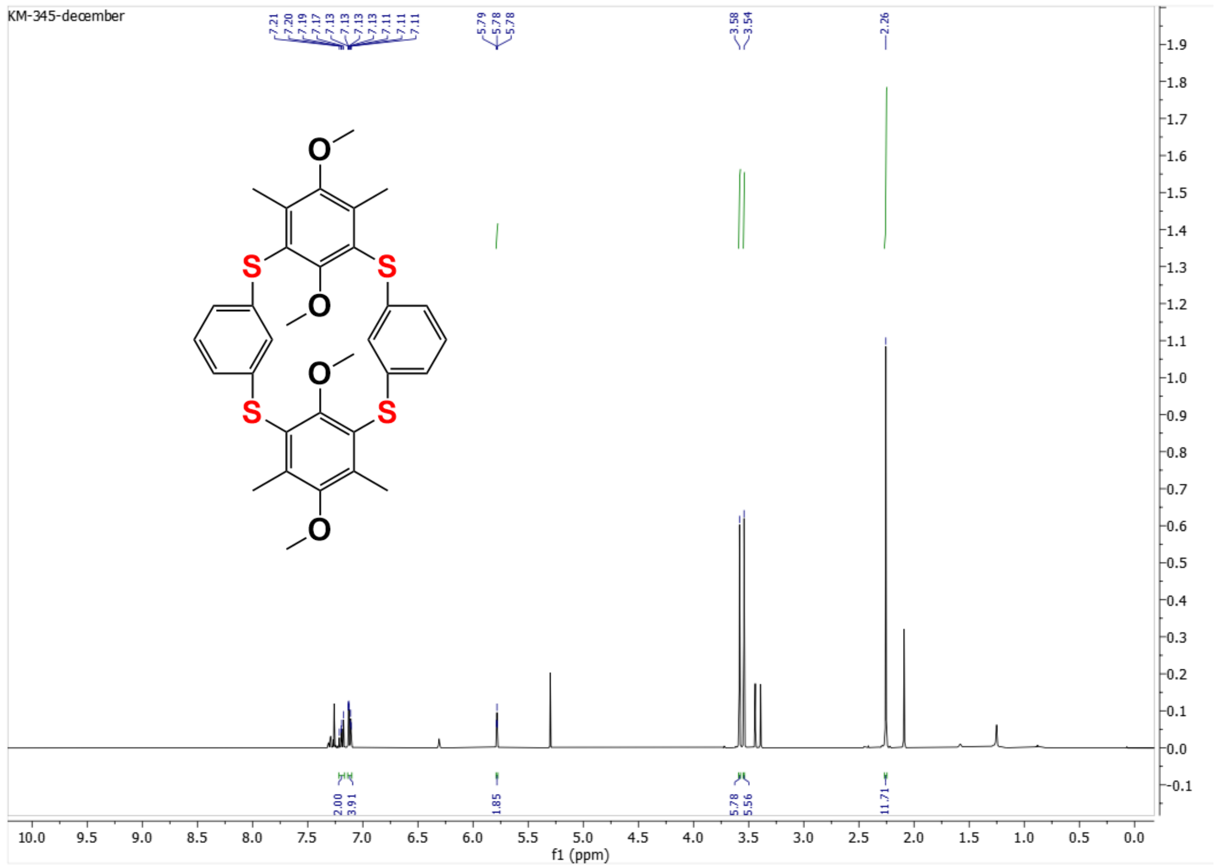

Figure S27: ^{1}H NMR of compound **10a** ($CDCl_3$, 400 MHz).

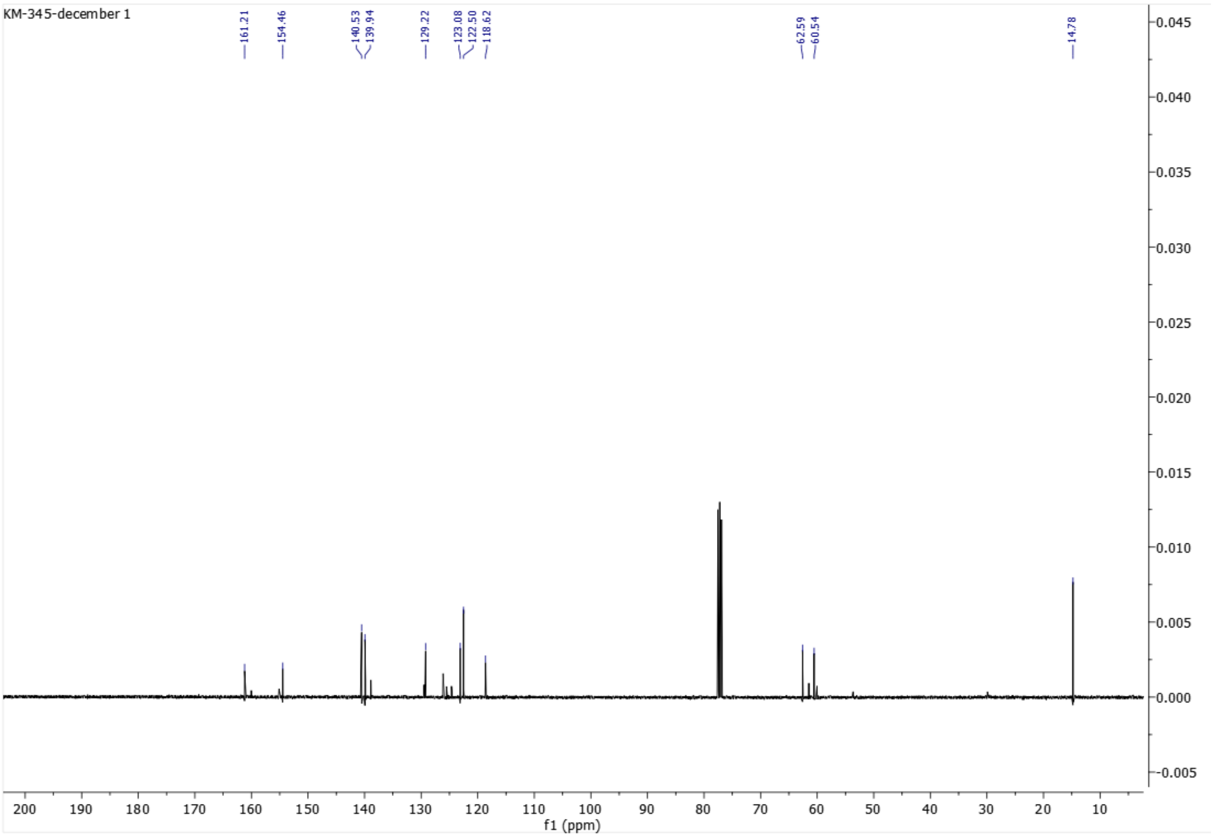

Figure S28: ^{13}C NMR of compound **10a** ($CDCl_3$, 100 MHz).

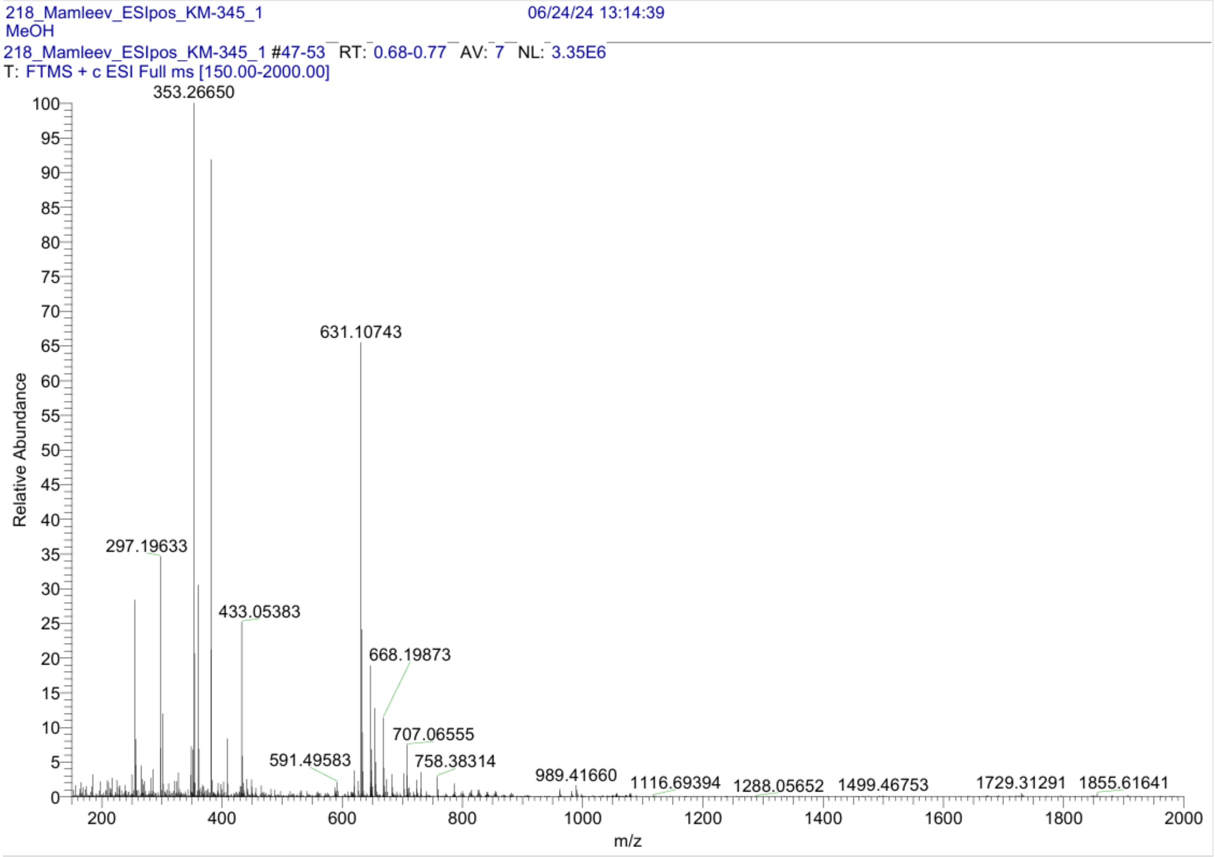

Figure S29: Full spectrum HRMS of compound **10a** (ESI^+).

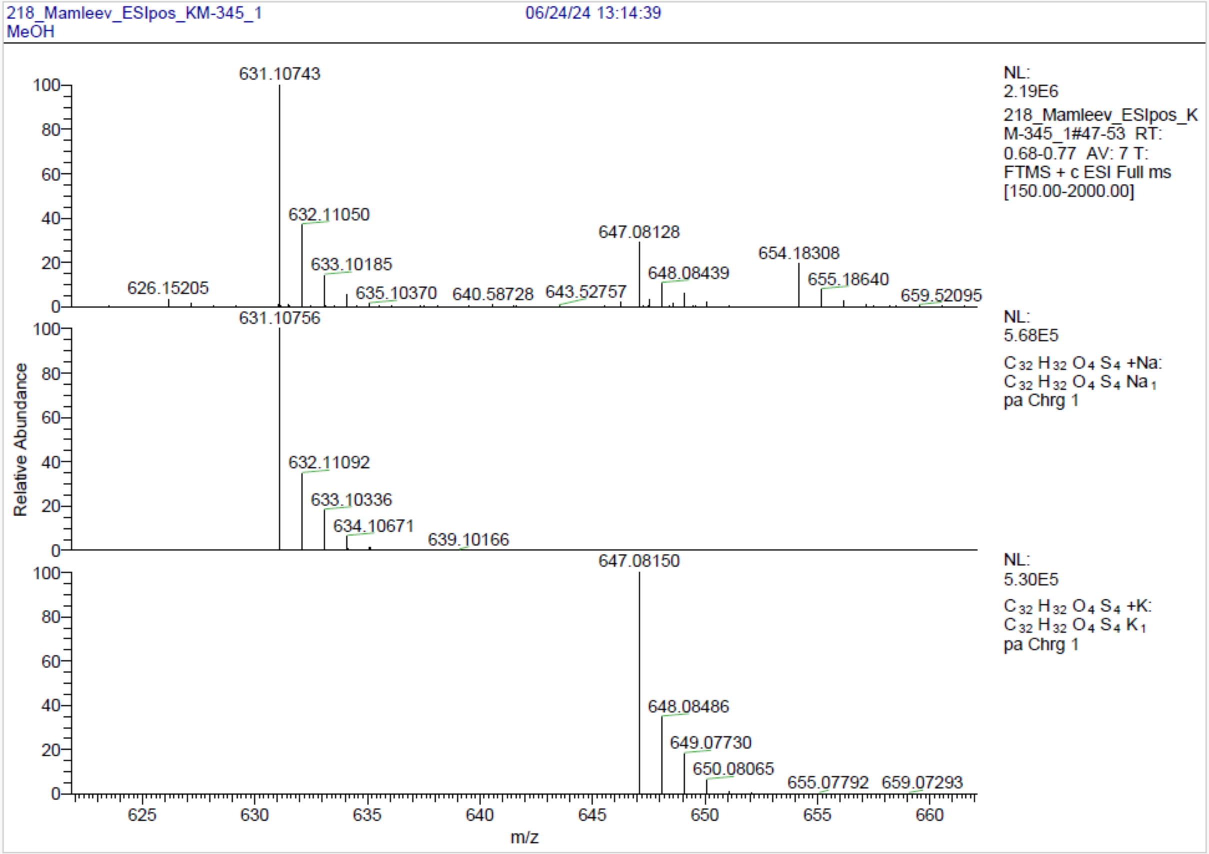

Figure S30: HRMS of compound **10a** (ESI$^+$).

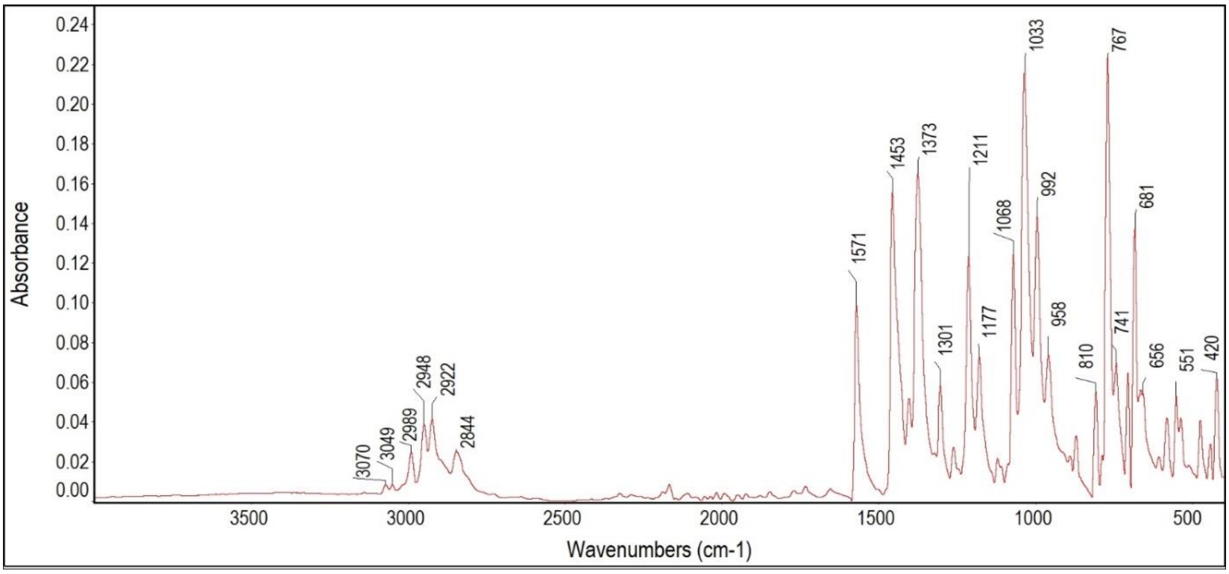

Figure S31: IR spectrum (ATR)of compound **10a**.

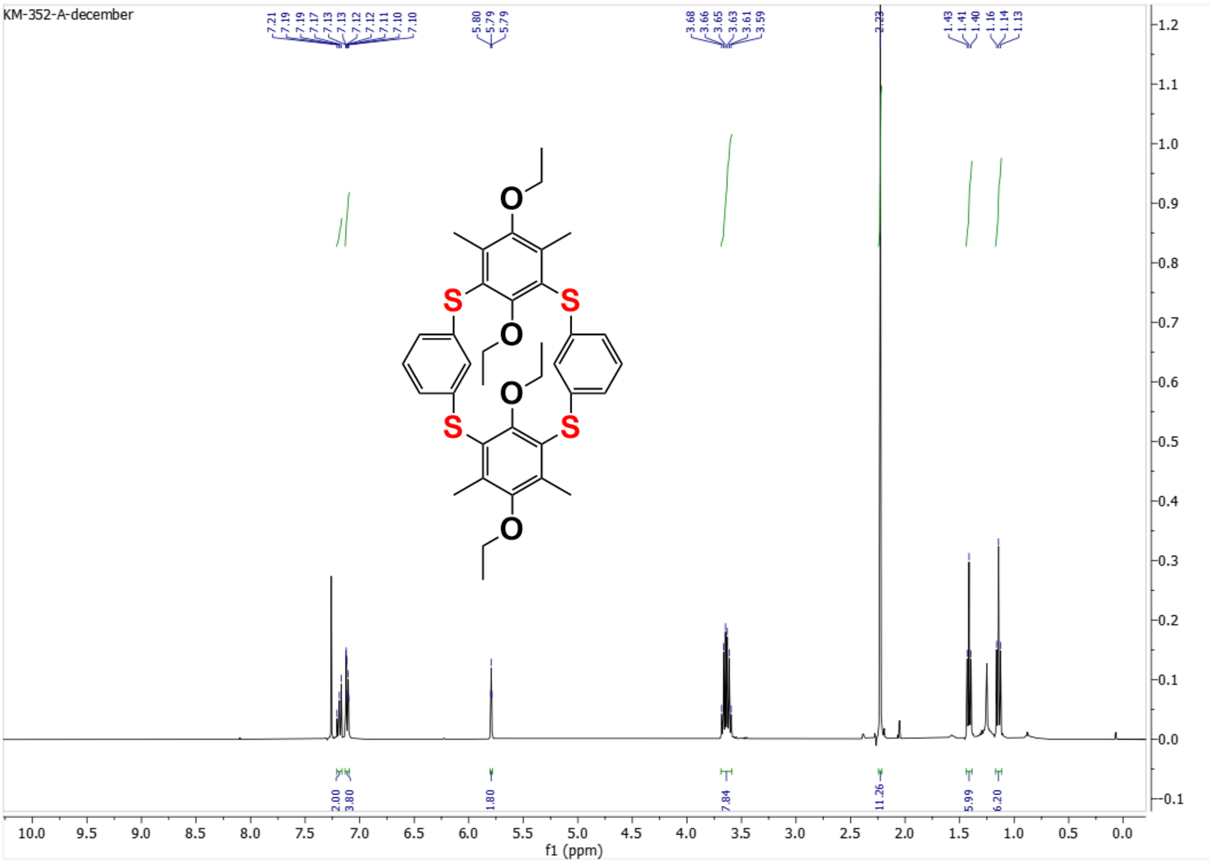

Figure S32: ^{1}H NMR of compound **10b** ($CDCl_3$, 400 MHz).

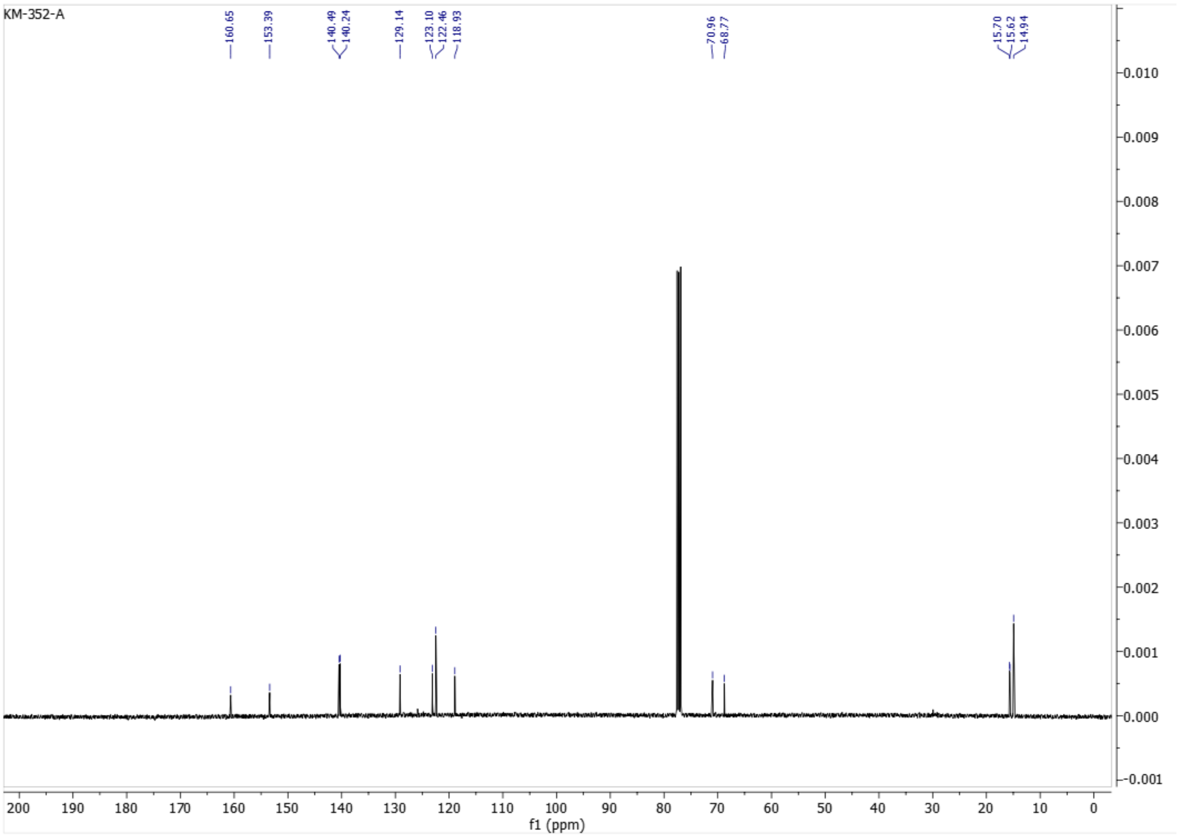

Figure S33: ^{13}C NMR of compound **10b** ($CDCl_3$, 100 MHz).

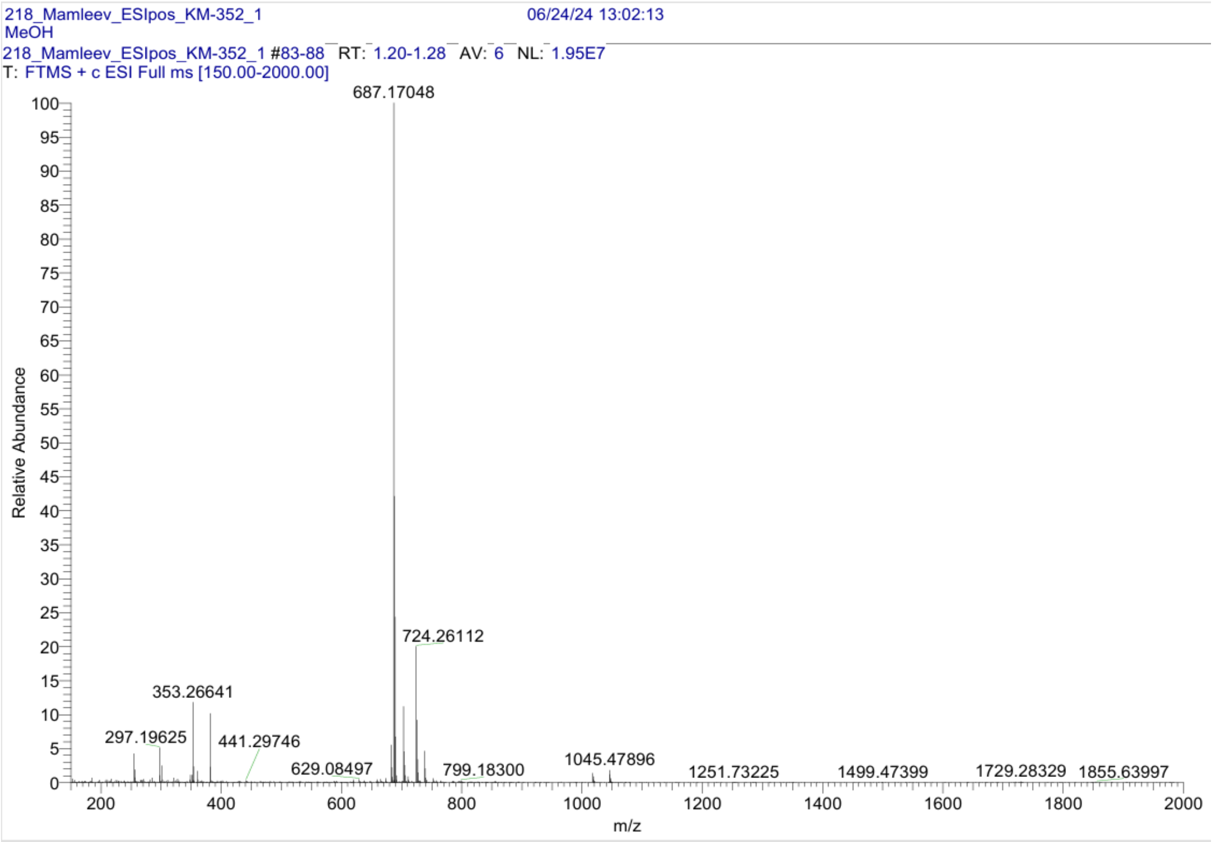

Figure S34: Full spectrum HRMS of compound **10b** (ESI^{+}).

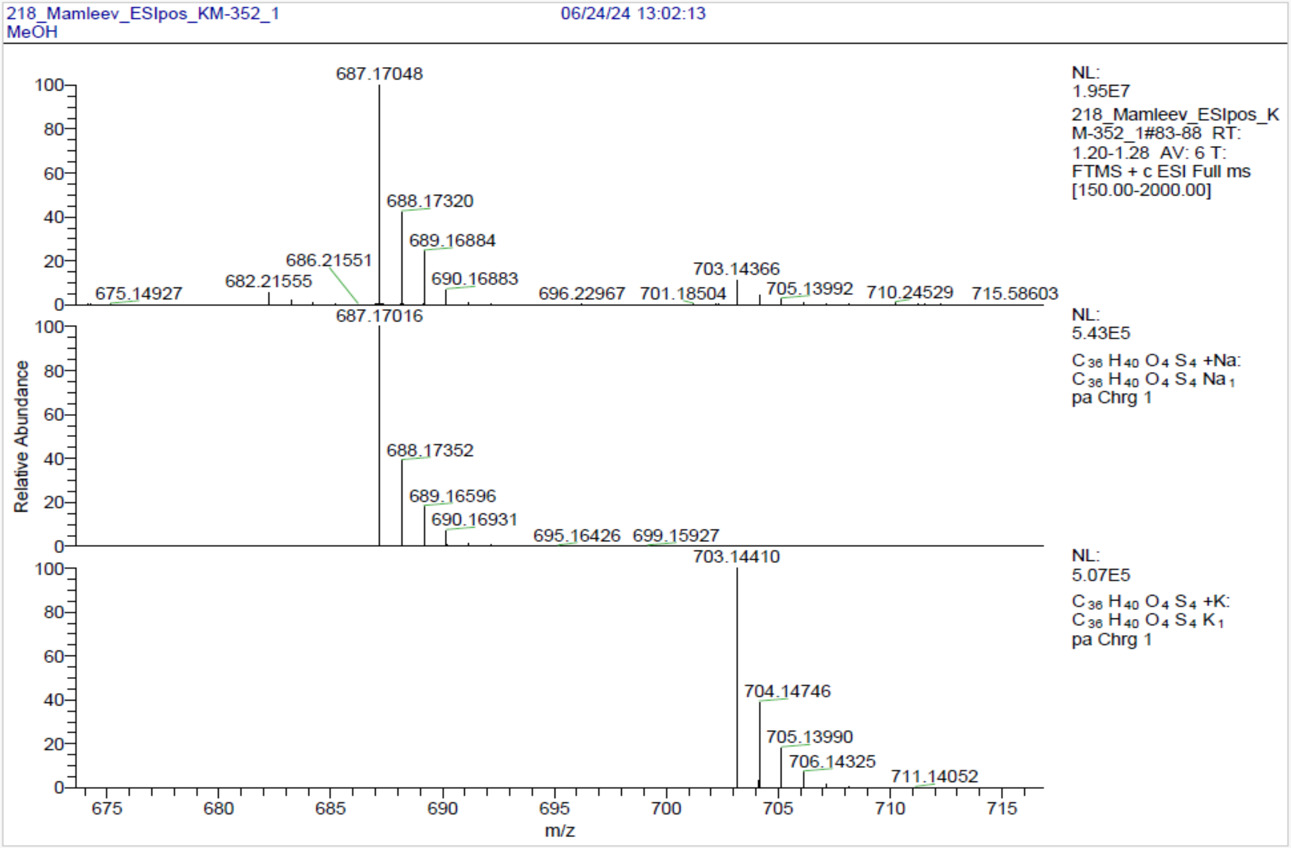

Figure S35: HRMS of compound **10b** (ESI^{+}).

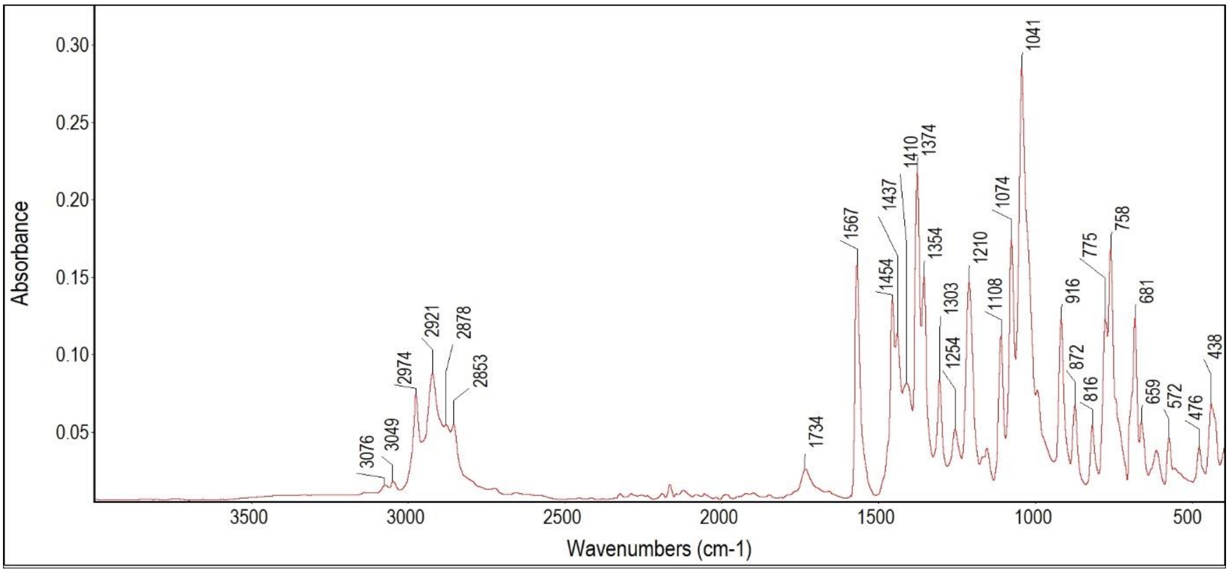

Figure S36: IR spectrum (ATR) of compound **10b**.

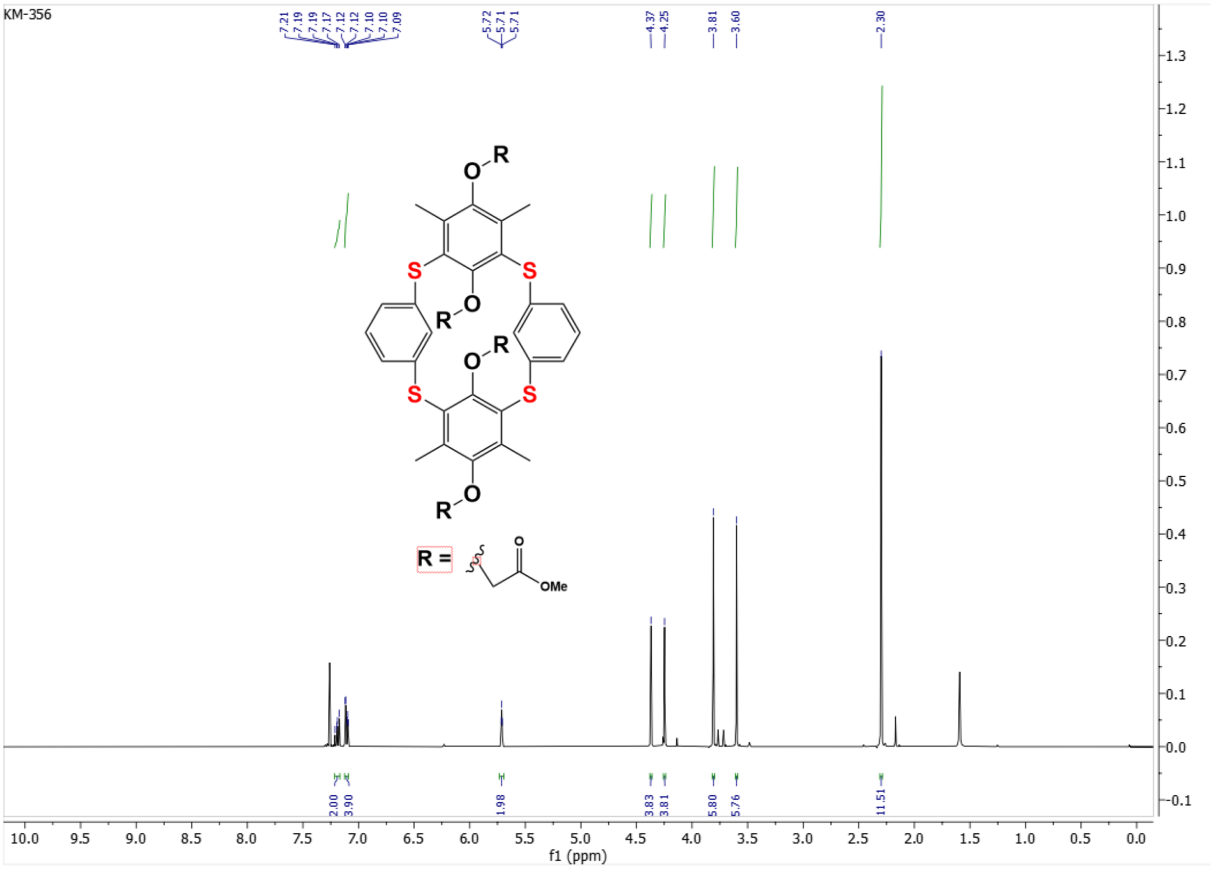

Figure S37: ^{1}H NMR of compound **10c** ($CDCl_3$, 400 MHz).

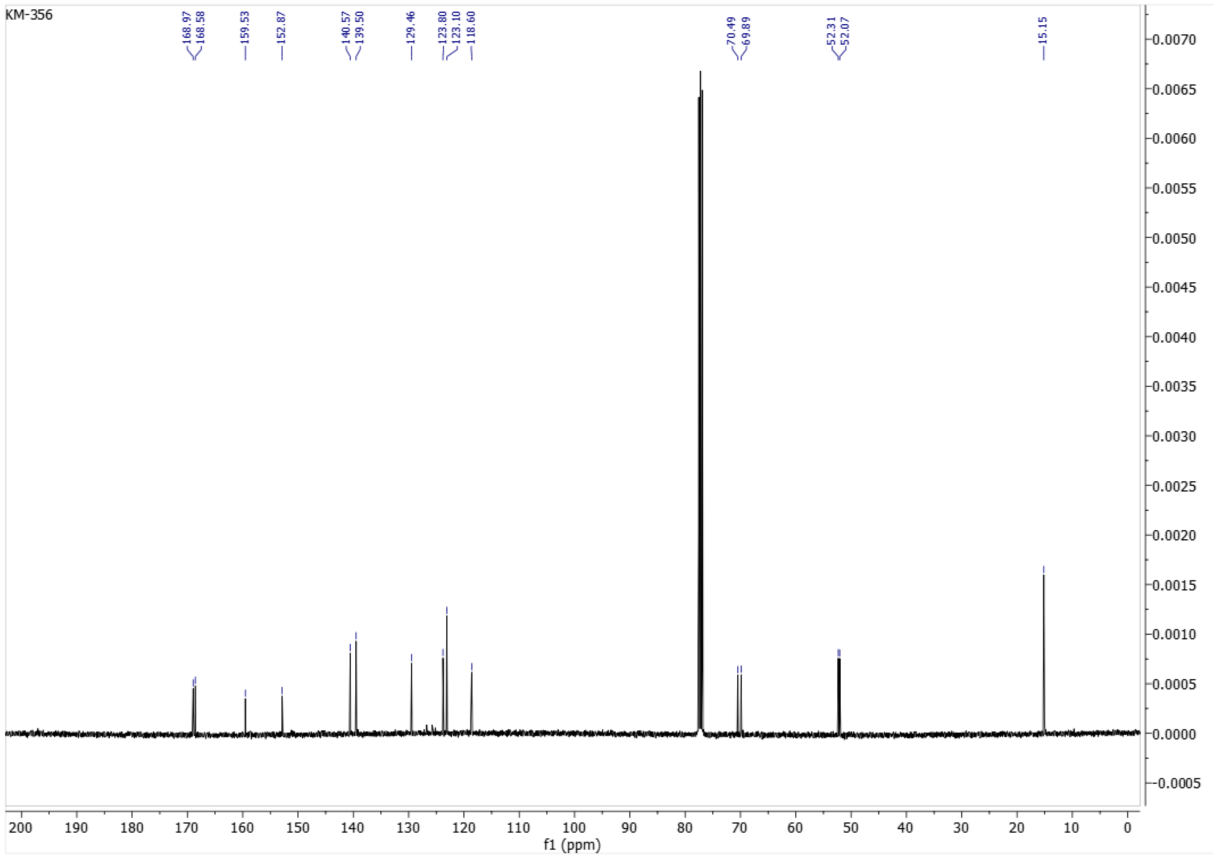

Figure S38: ^{13}C NMR of compound **10c** ($CDCl_3$, 100 MHz).

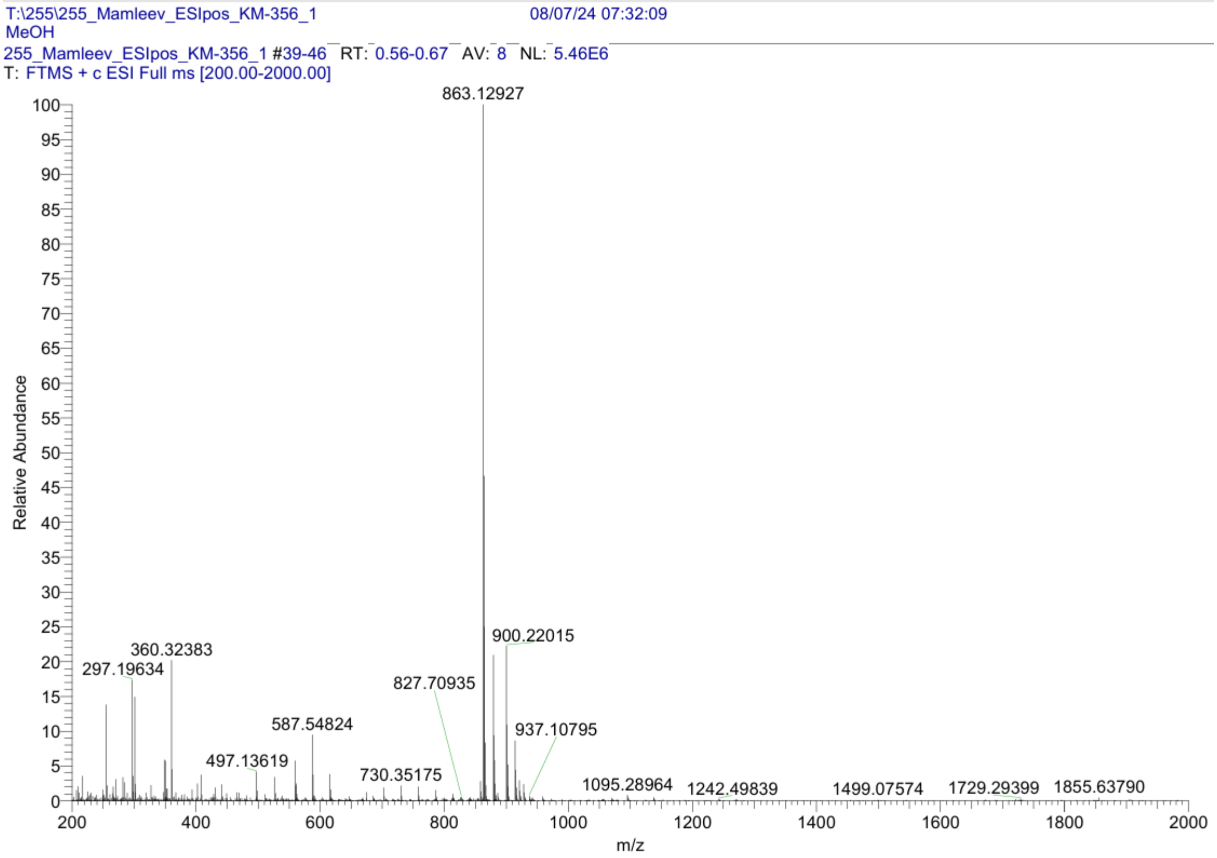

Figure S39: Full spectrum HRMS of compound **10c** (ESI+).

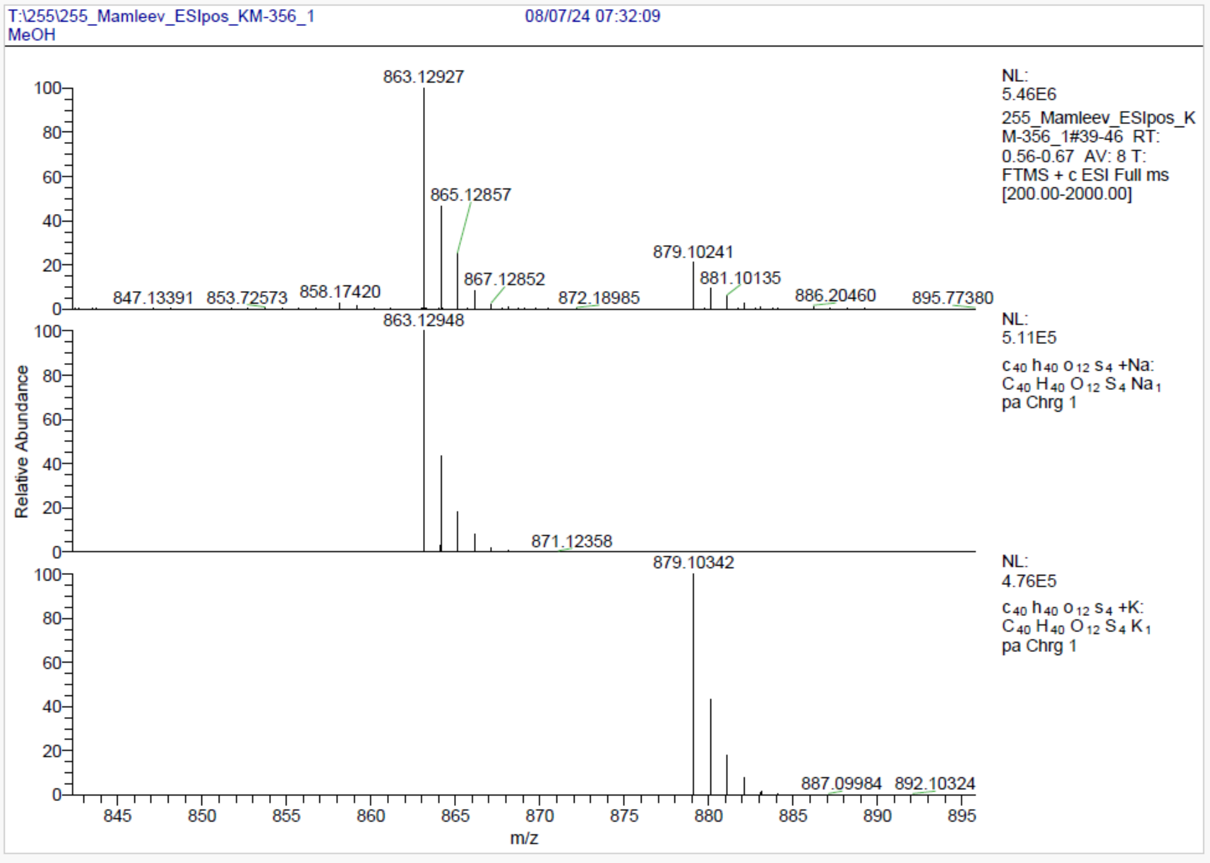

Figure S40: HRMS of compound **10c** (ESI+).

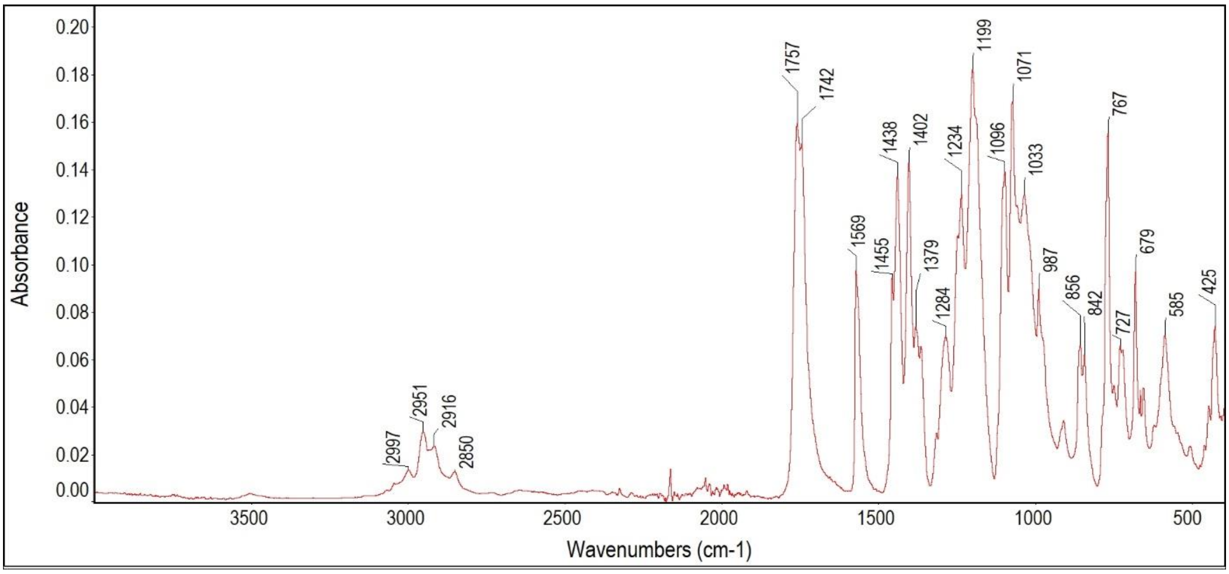

Figure S41: IR spectrum (ATR) of compound **10c**.

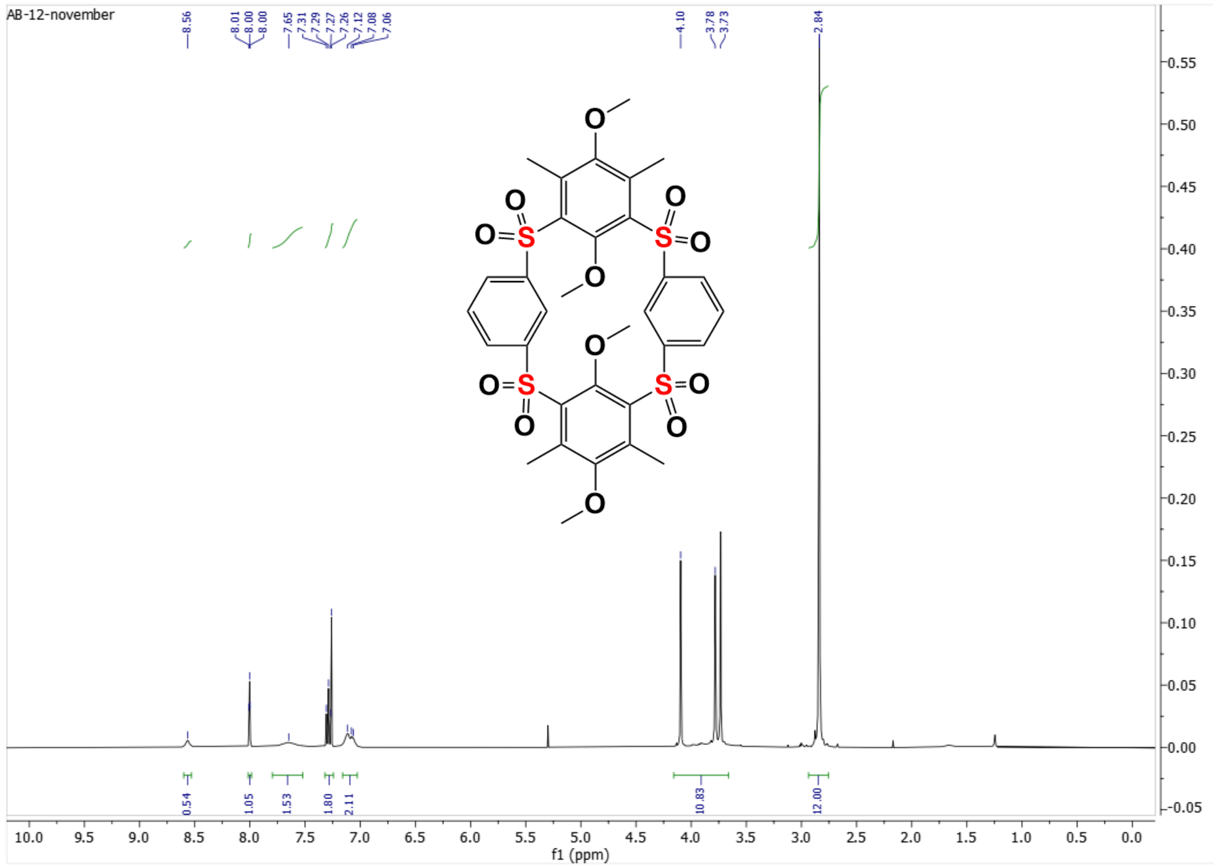

Figure S42: ^{1}H NMR of compound **11** ($CDCl_3$, 400 MHz).

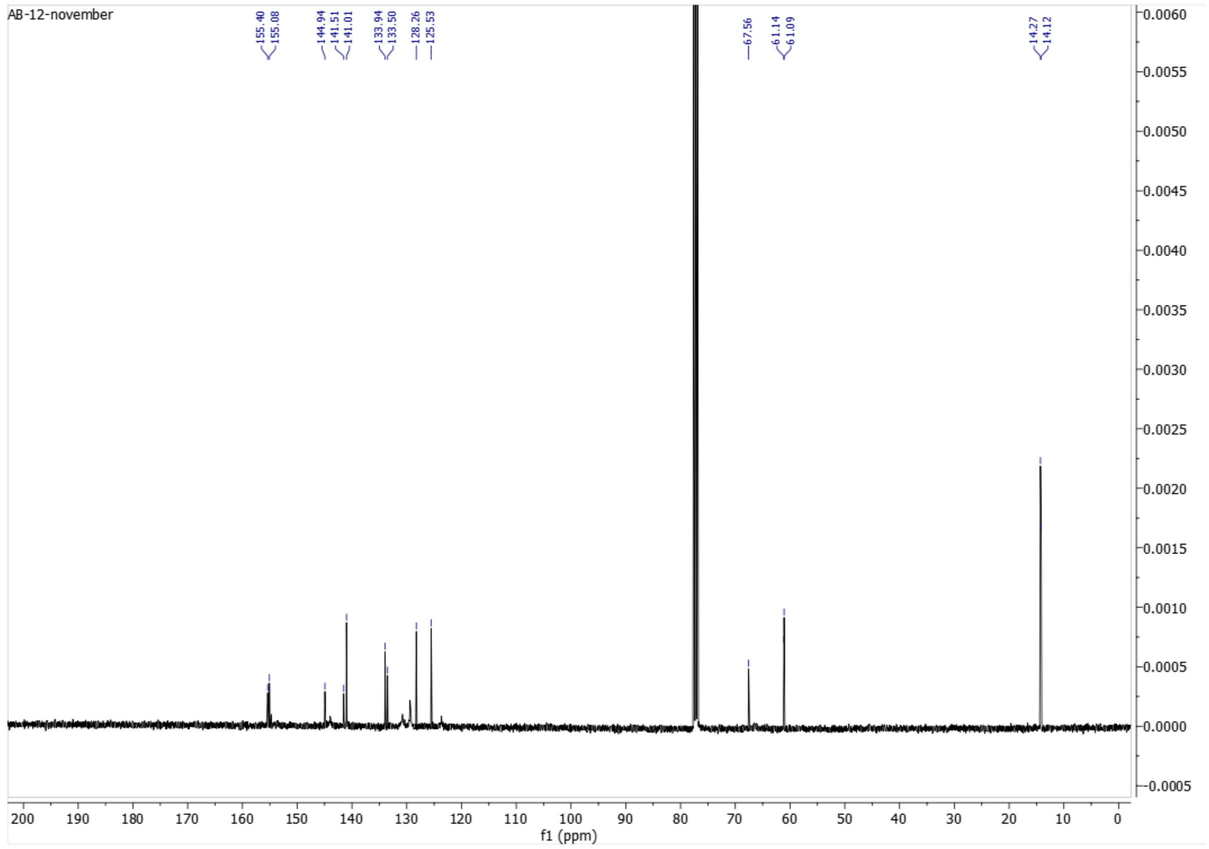

Figure S43: ^{13}C NMR of compound **11** ($CDCl_3$, 100 MHz).

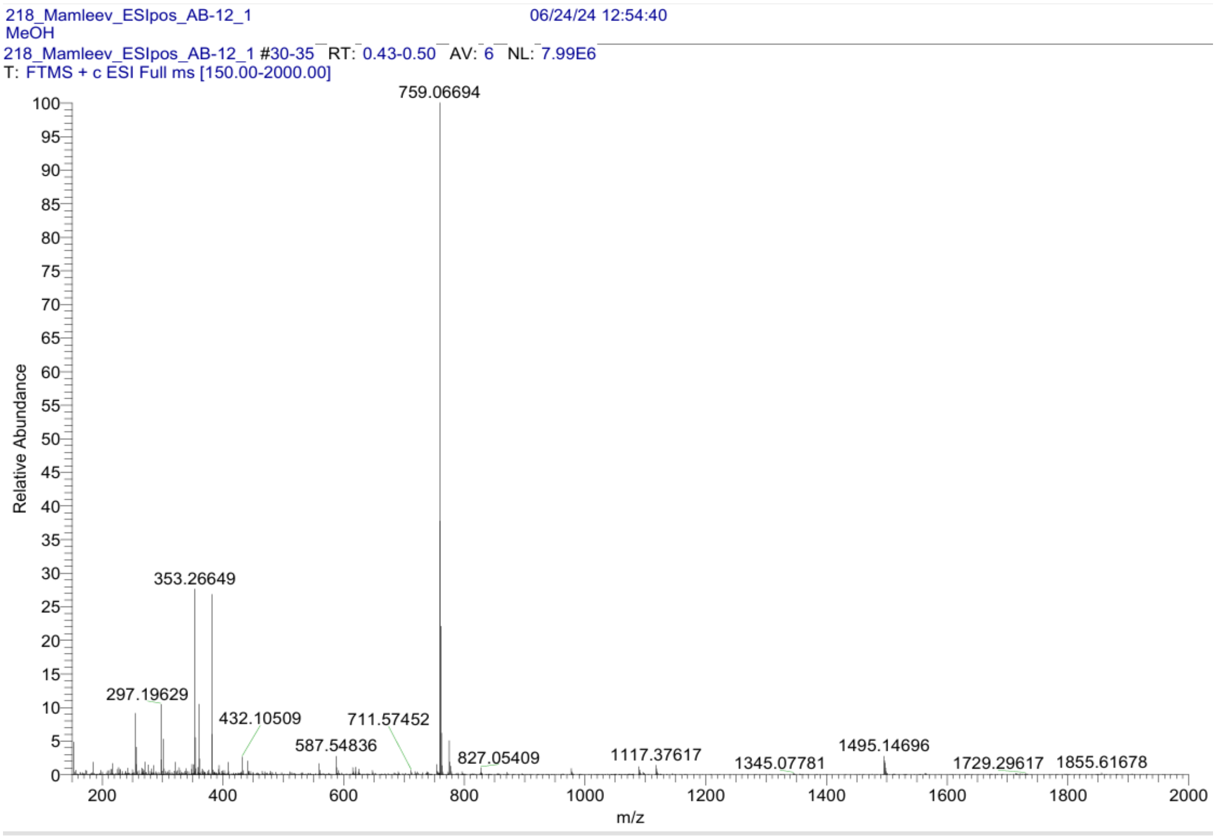

Figure S44: Full spectrum HRMS of compound **11** (ESI^+).

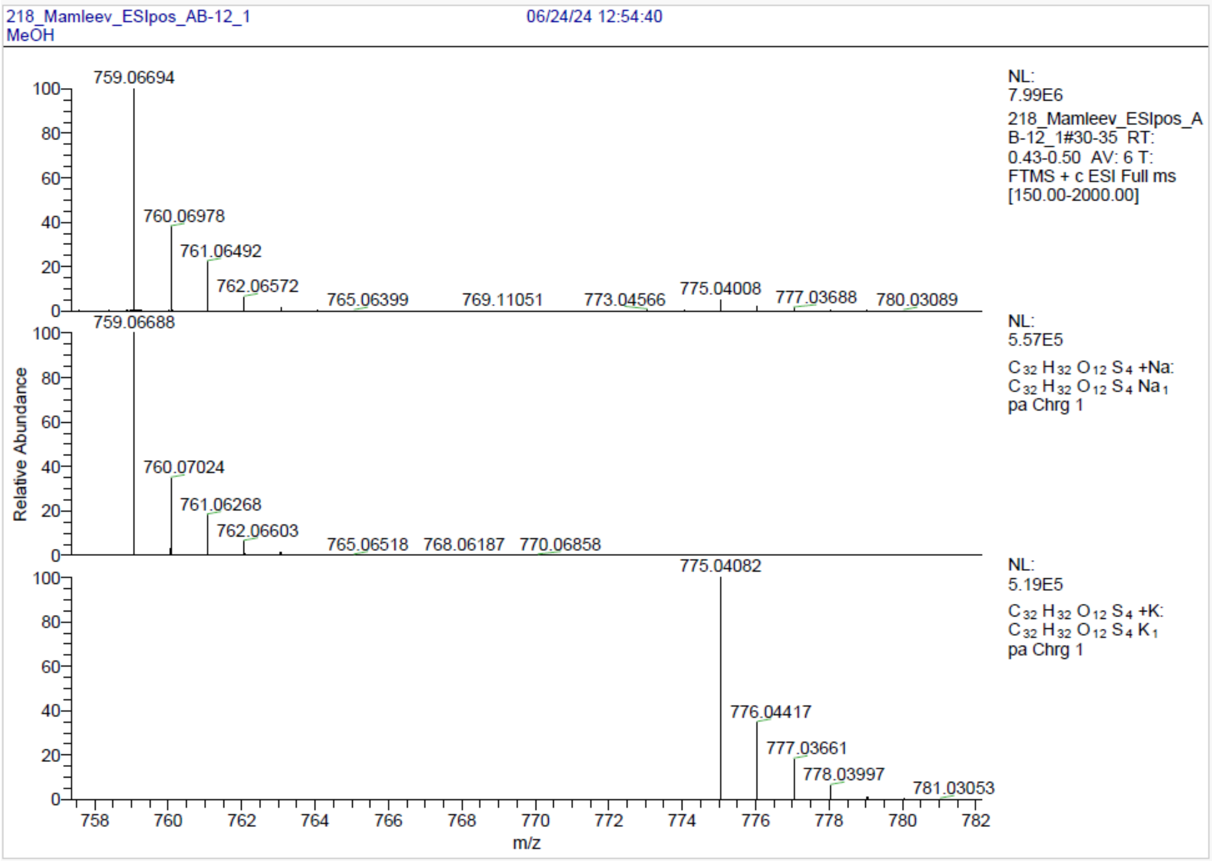

Figure S45: HRMS of compound **11** (ESI^+).

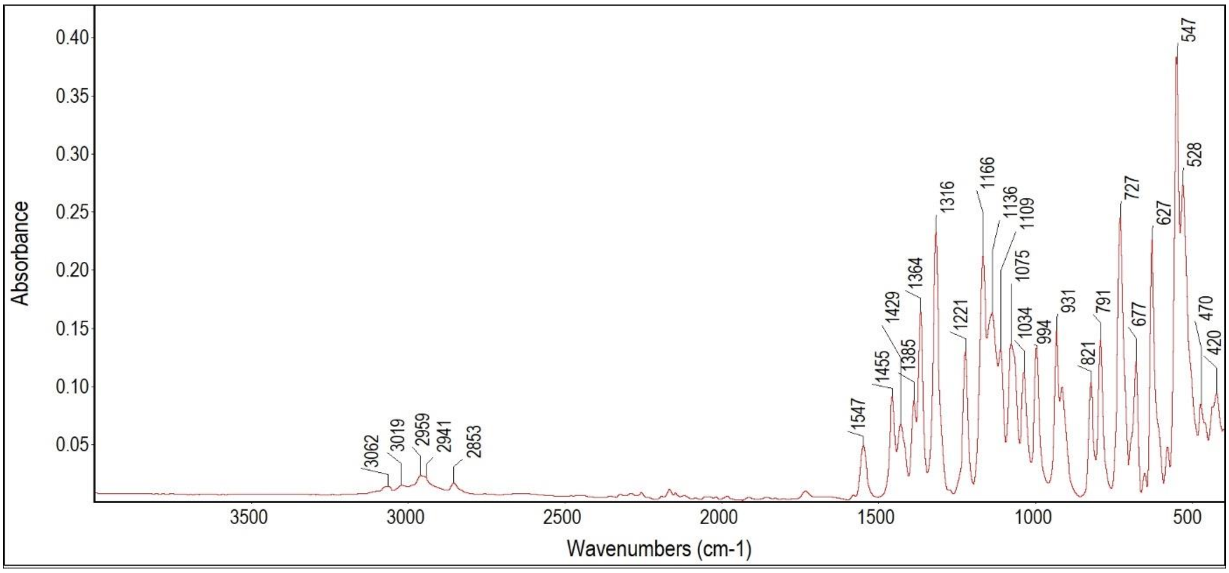

Figure S46: IR spectrum (ATR) of compound **11**.

3. Dynamic NMR experiments

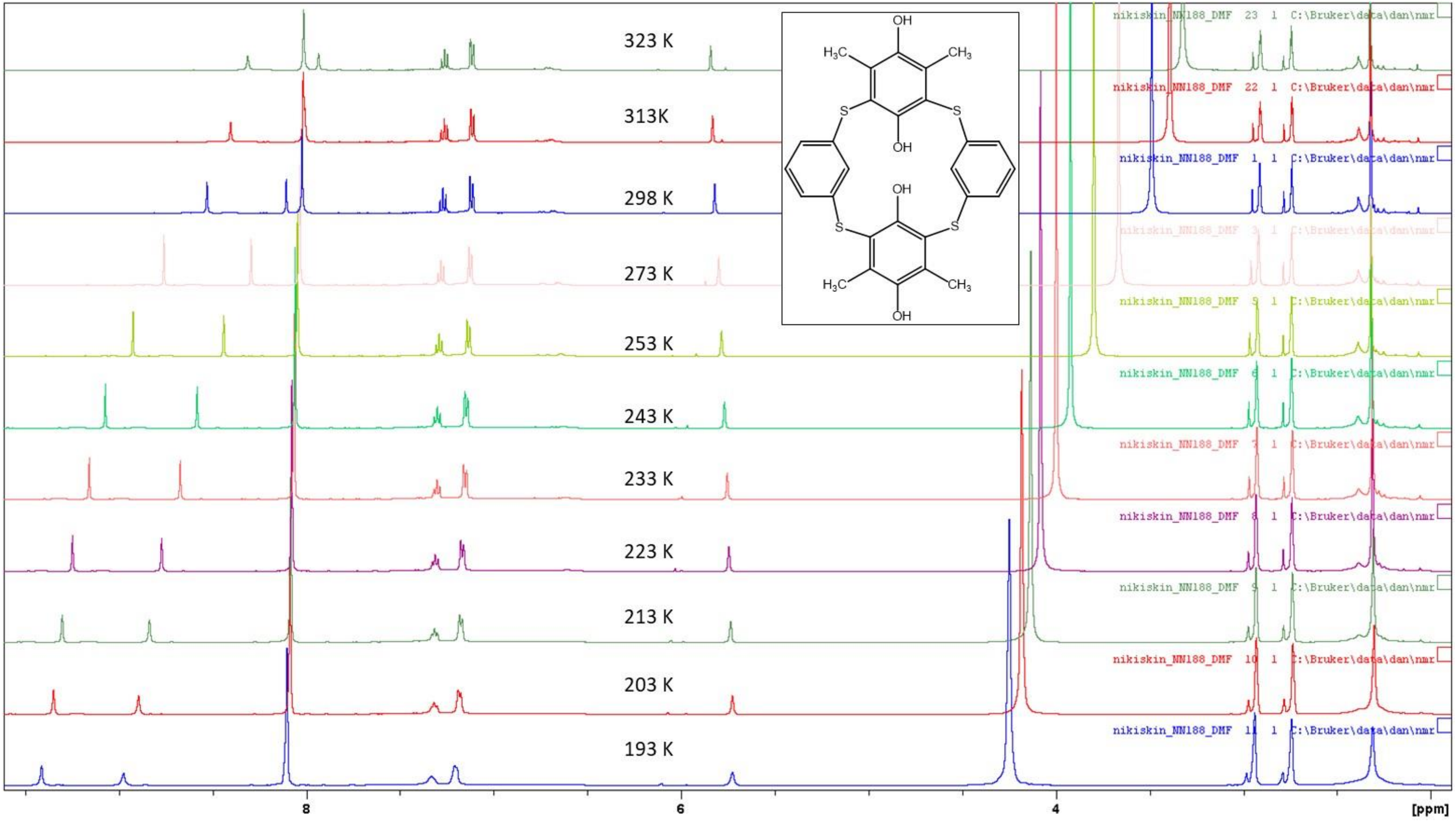

Figure S47: VT ^{1}H NMR spectra (CD_2Cl_2, 500 MHz) of compound **9**.

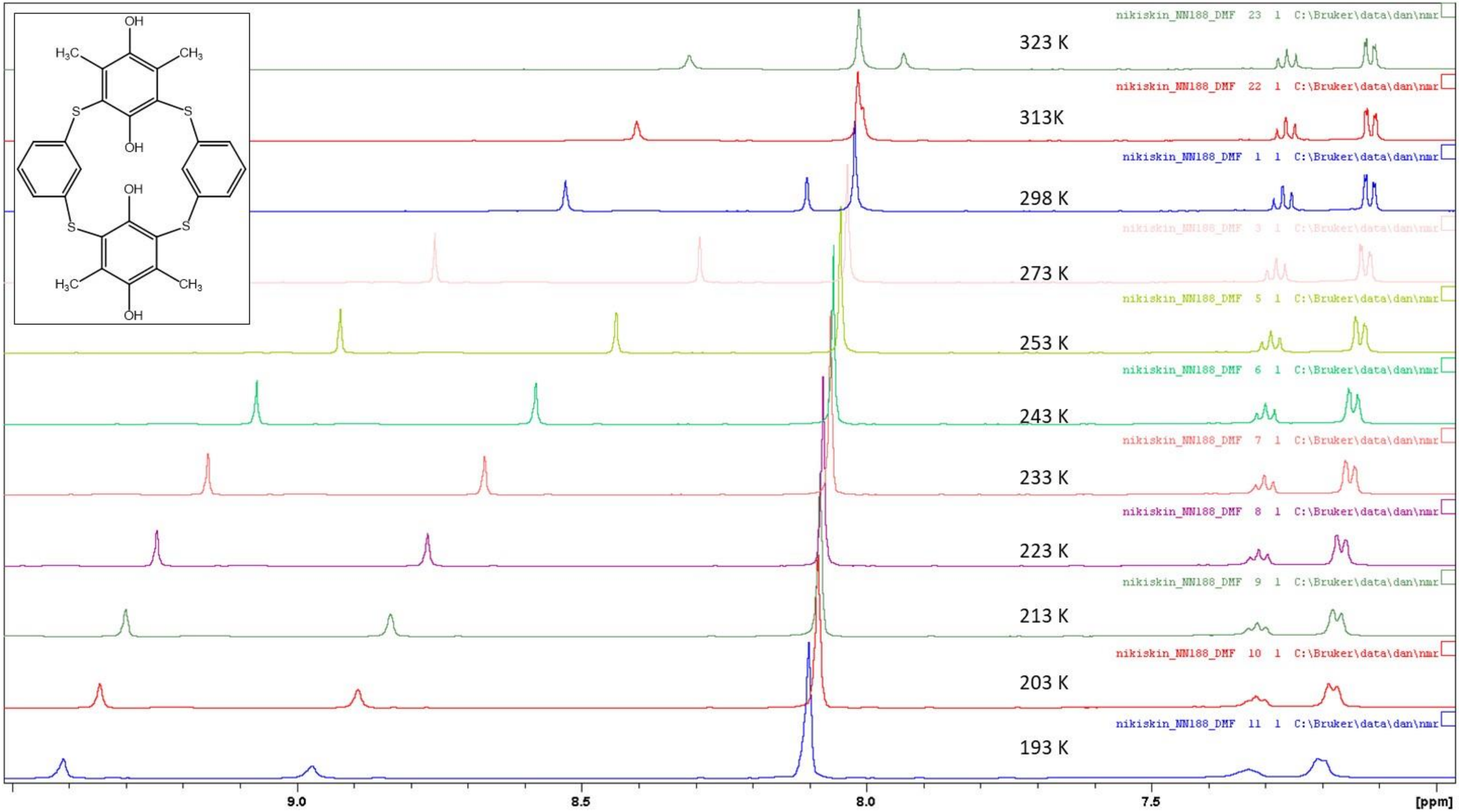

Figure S48: VT ^{1}H NMR spectra (CD_2Cl_2, 500 MHz) of compound **9** (aromatic region).

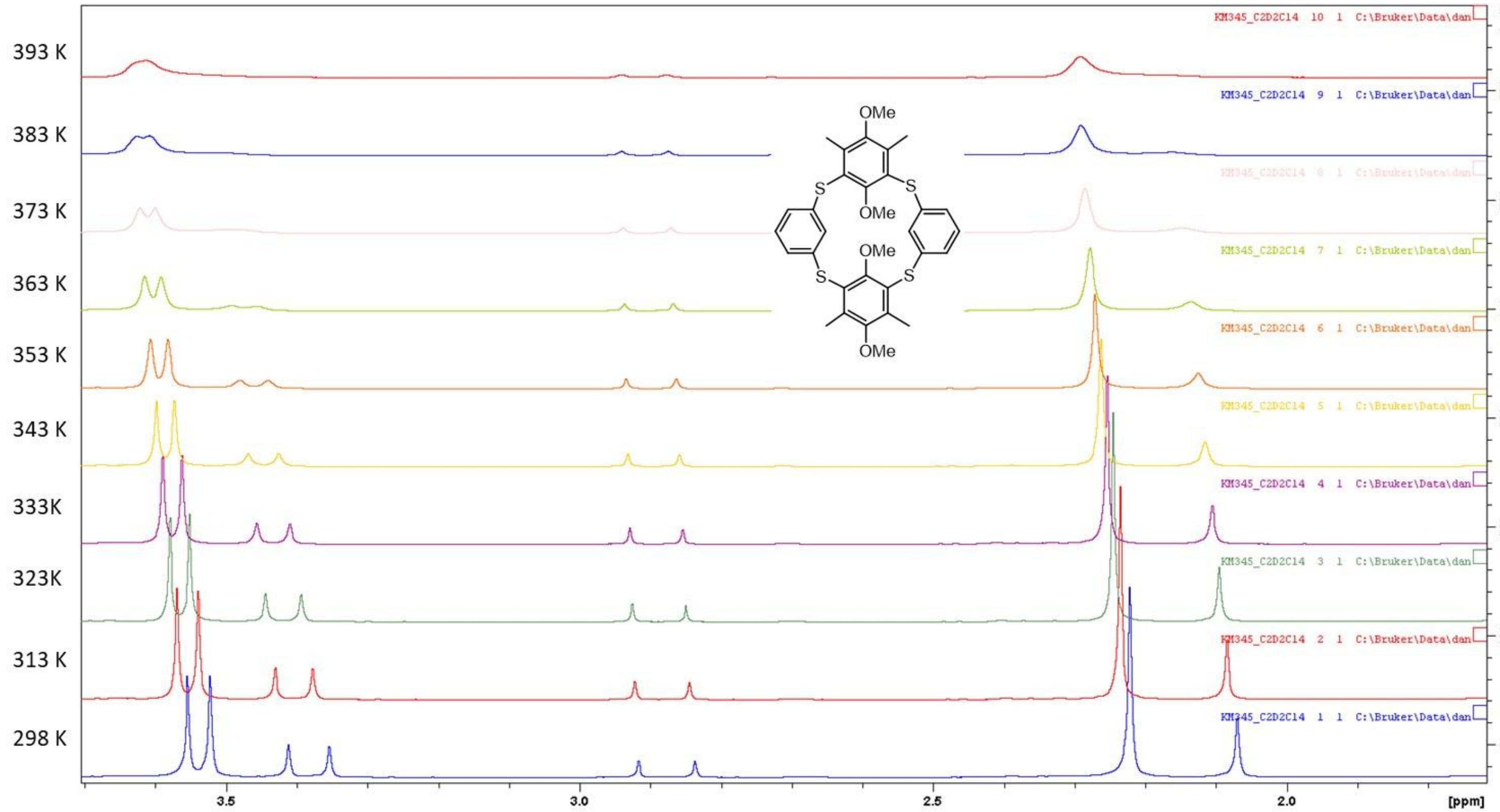

Figure S49: VT ^{1}H NMR spectra (CD_2Cl-CD_2Cl, 500 MHz) of compound **10a** (aliphatic region).

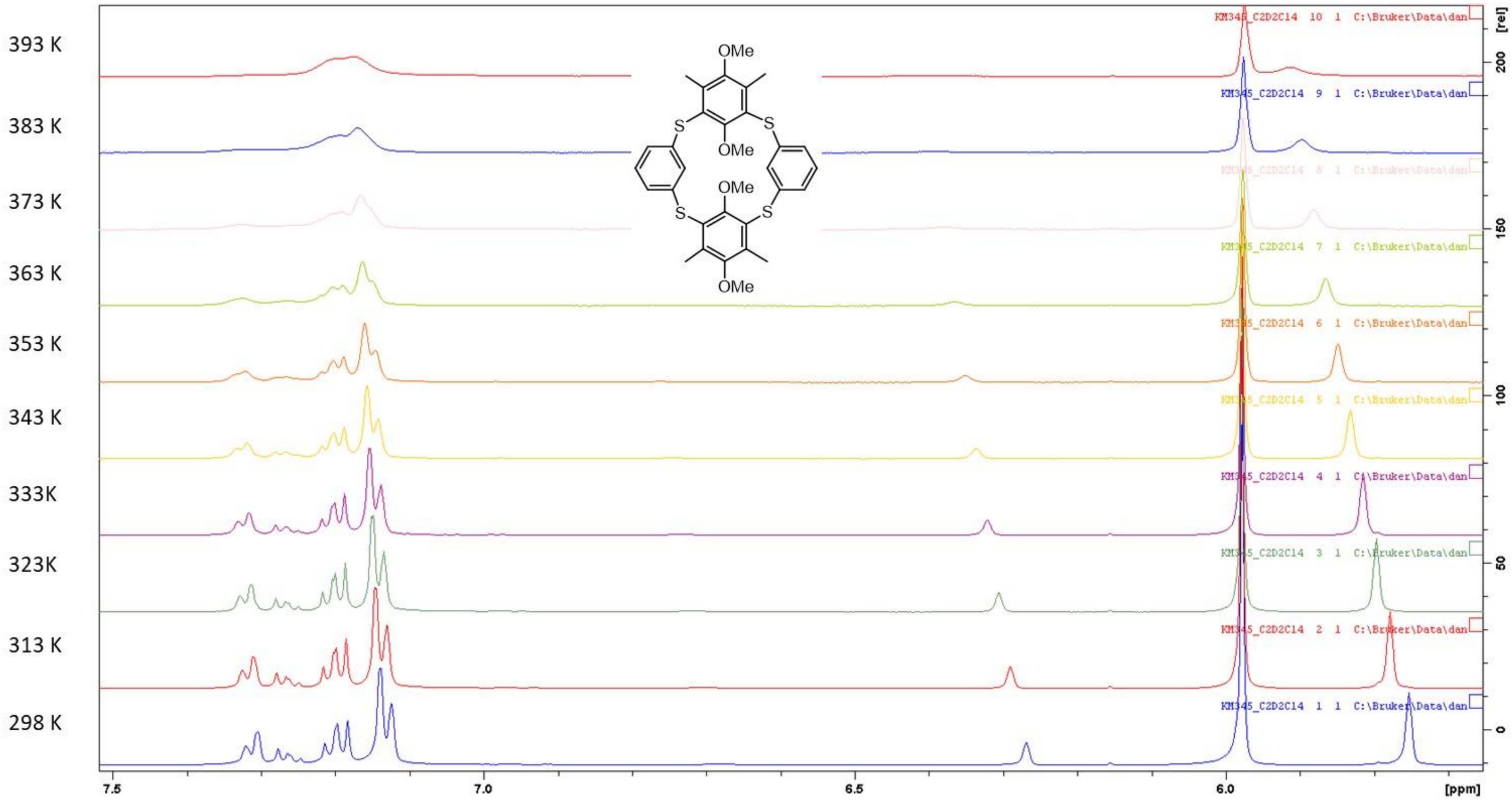

Figure S50: VT ^{1}H NMR spectra (CD_2Cl-CD_2Cl, 500 MHz) of compound **10a** (aromatic region).

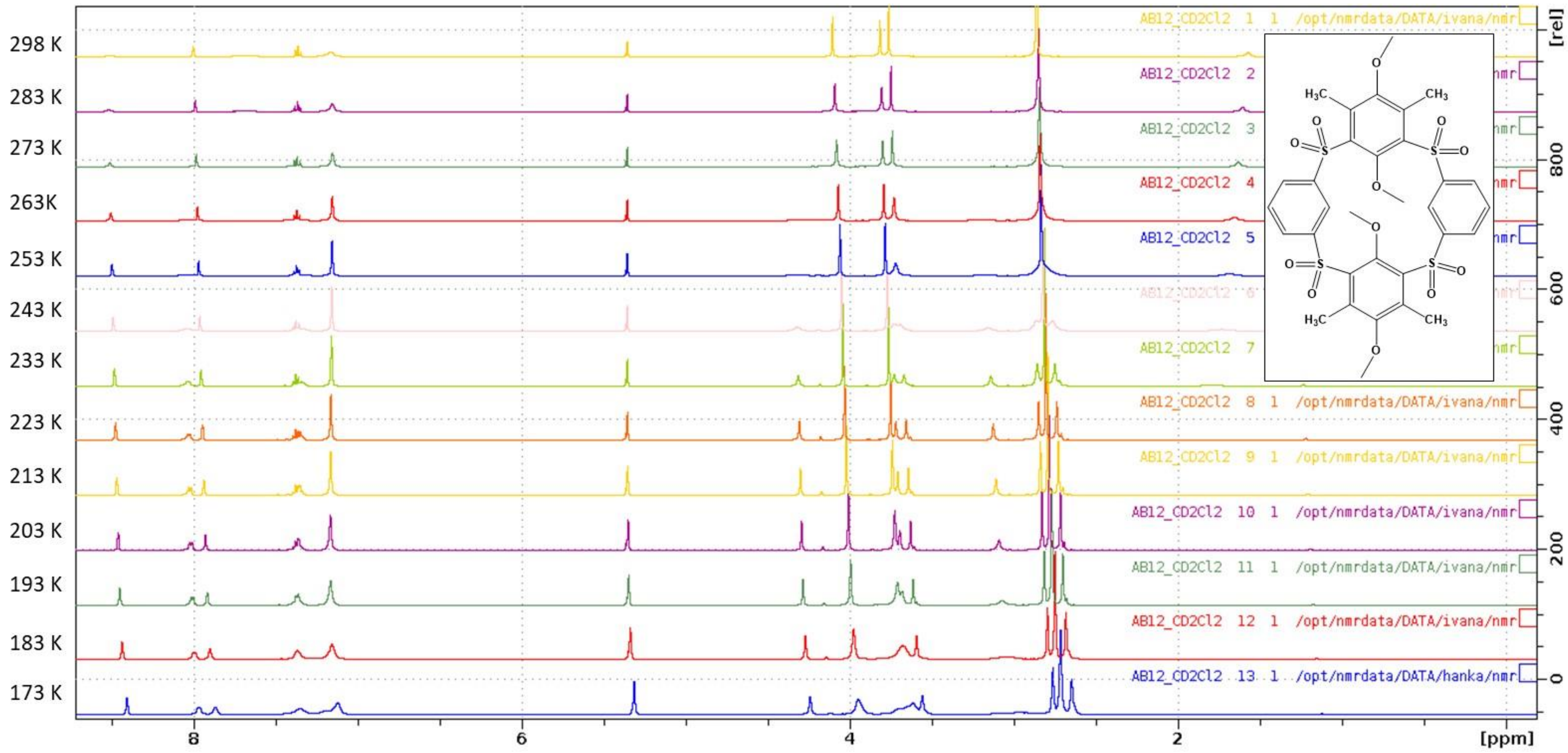

Figure S51a: VT ^{1}H NMR spectra (CD_2Cl-CD_2Cl, 500 MHz) of compound **11** (whole spectra).

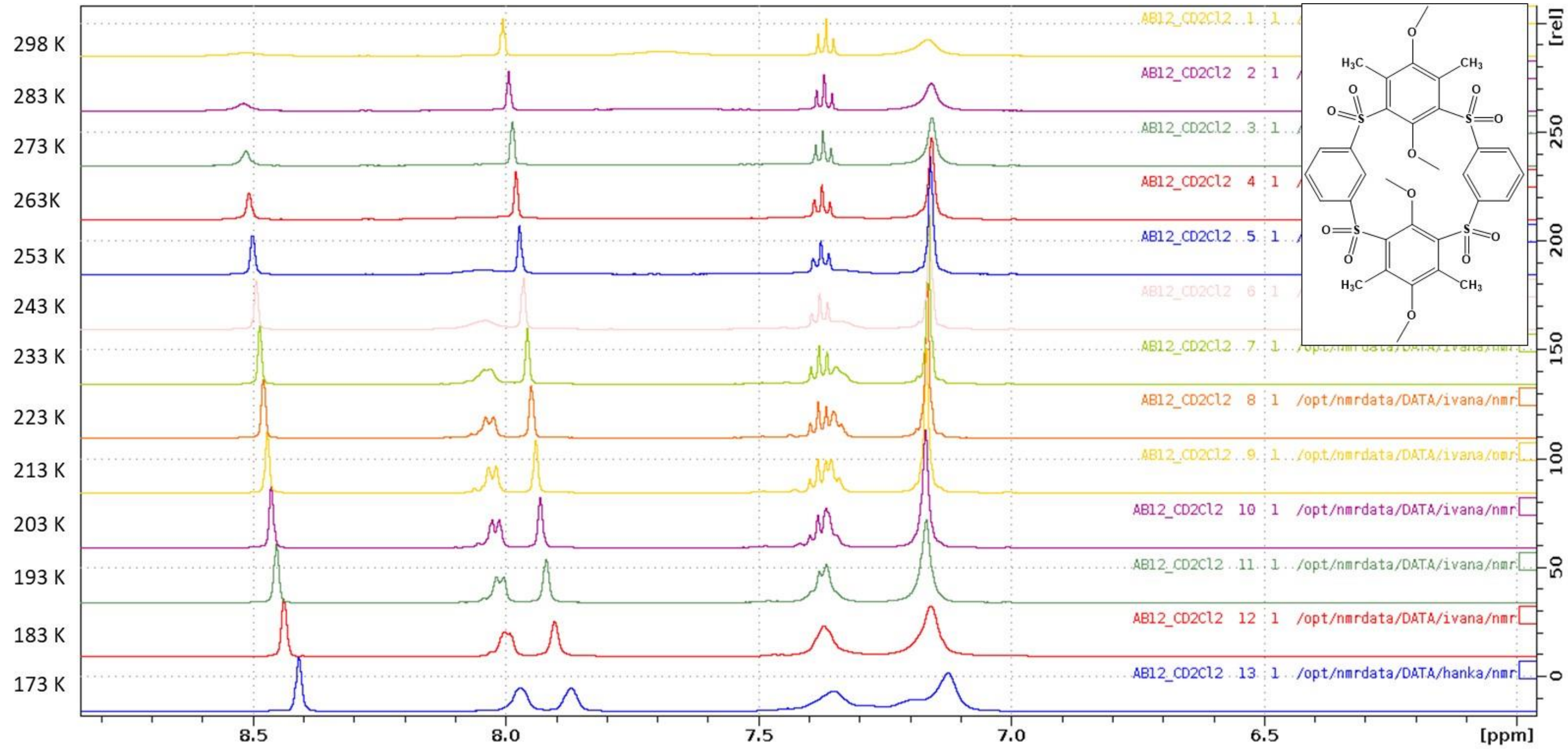

Figure S51b: VT ^{1}H NMR spectra (CD_2Cl-CD_2Cl, 500 MHz) of compound **11** (aromatic region).

4. Electrochemical measurements

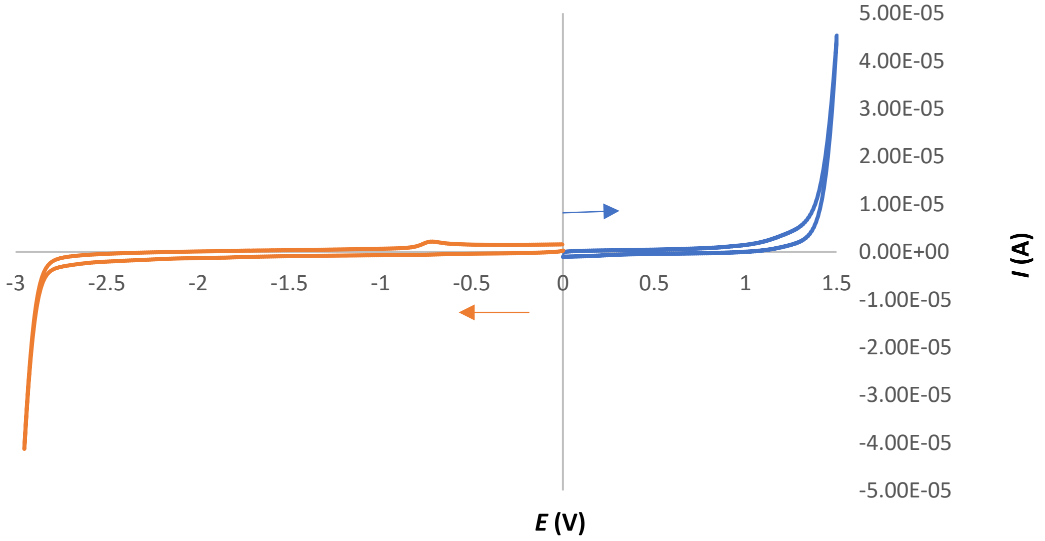

Figure S52: Cyclic voltammograms (W = GC, Ref = SCE, Aux = Pt) of typical blank experiment of DMSO (0.1 M $TBAHPF_6$) measured with scan rate 100 mV/s – orange (reduction: potential window from 0 to -2.95 V); blue (oxidation: potential window from 0 to 1.5 V).

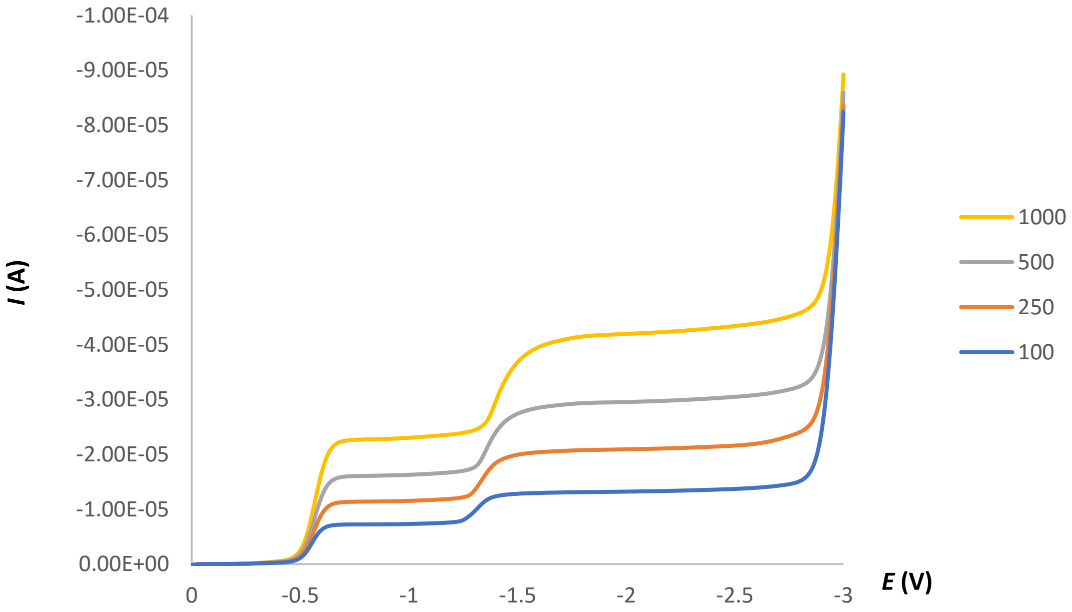

Figure S53: Linear sweep voltammetry of **1c** (1.09 mM) in DMSO (0.1 M $TBAHPF_6$) (W = RDE = GC; Ref = SCE; Aux = Pt) was measured with scan rate of 10 mV/s, using several rotating rates (100, 250, 500 and 1000 s^{-1}) in potential window from 0 to -3.0 V.

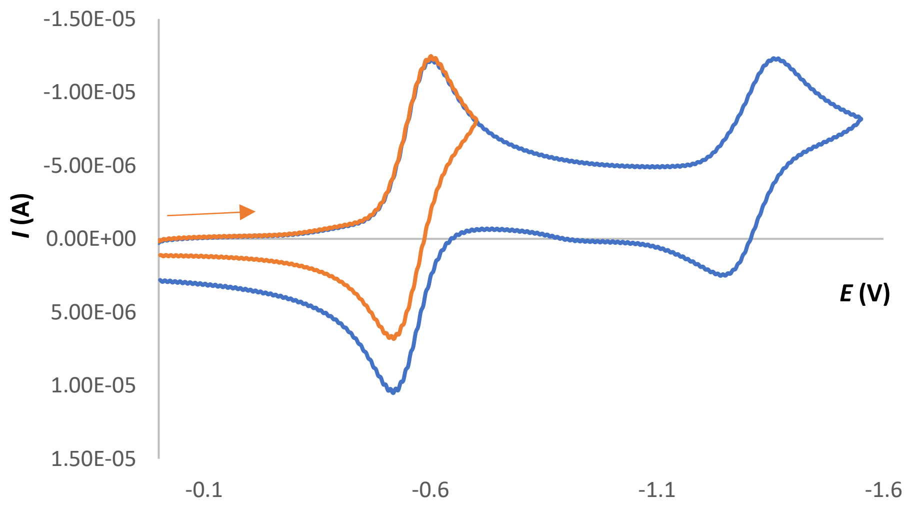

Figure S54: Reductive cyclic voltammogram (W = GC, Ref = SCE, Aux = Pt) of compound **1c** (1.09 mM) in DMSO (0.1 M TBAHPF$_6$) measured with scan rate of 100 mV/s (polarographic plotting convention).

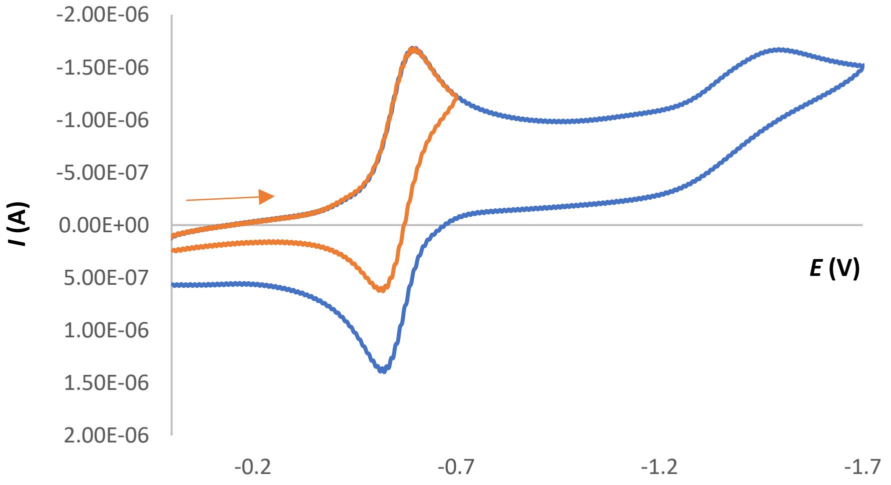

Figure S55: Reductive cyclic voltammogram (W = Pt, Ref = SCE, Aux = Pt) of compound **1c** (1.09 mM) in DMSO (0.1 M TBAHPF$_6$) measured with scan rate of 100 mV/s (polarographic plotting convention).

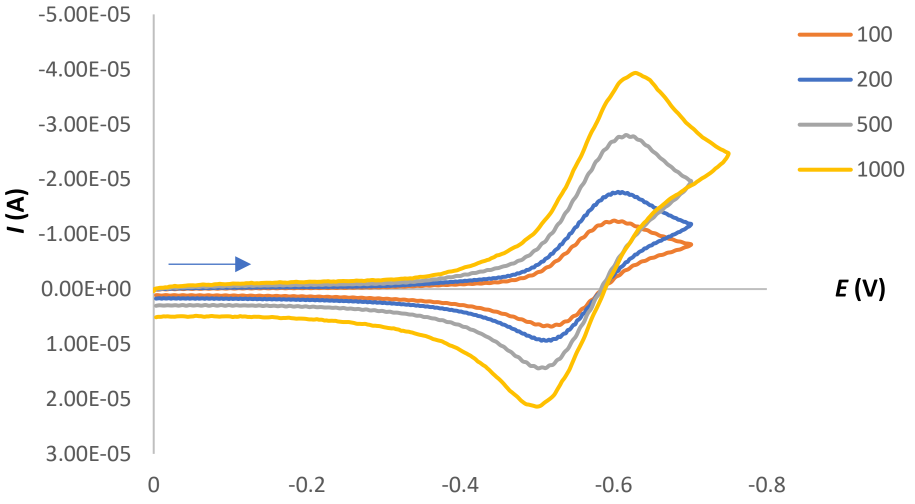

Figure S56: Reductive cyclic voltammograms (W = GC, Ref = SCE, Aux = Pt) of the 1st peak of compound **1c** (1.09 mM) in DMSO (0.1 M TBAHPF$_6$) measured with several different scan rates (polarographic plotting convention).

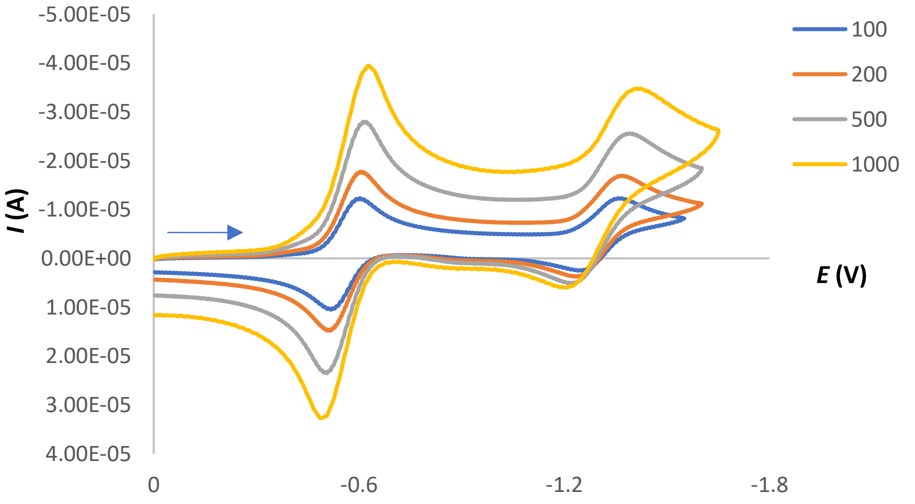

Figure S57: Reductive cyclic voltammograms (W = GC, Ref = SCE, Aux = Pt) of the 2nd peak of compound **1c** (1.09 mM) in DMSO (0.1 M TBAHPF$_6$) measured with several different scan rates (polarographic plotting convention).

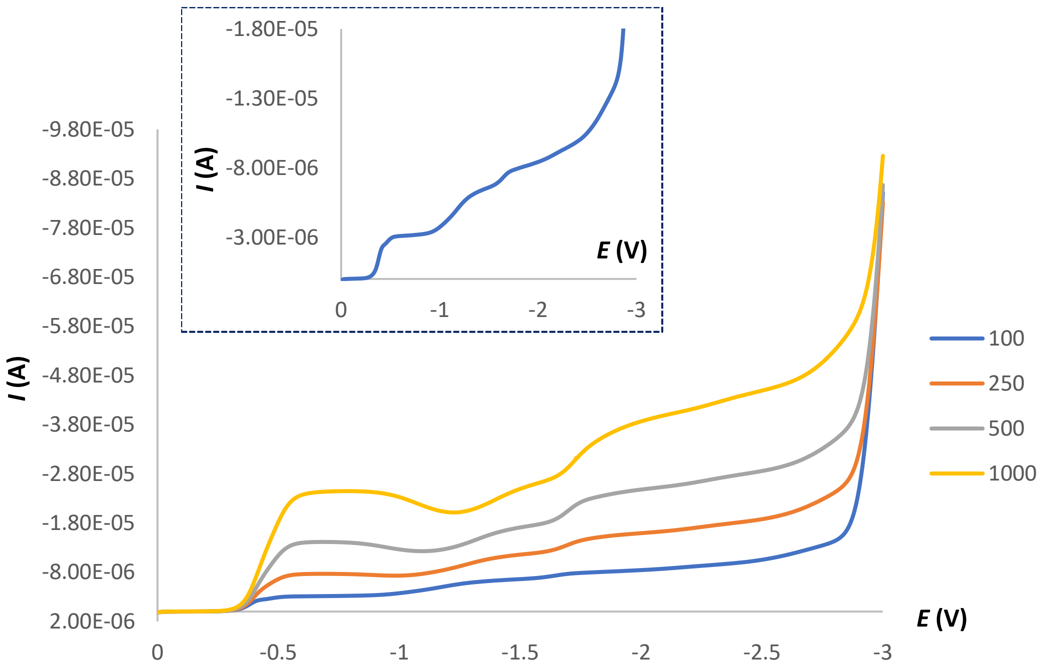

Figure S58: Linear sweep voltammetry of **8** (1.00 mM) in DMSO (0.1 M TBAHPF$_6$) (W = RDE = GC; Ref = SCE; Aux = Pt) was measured with scan rate of 10 mV/s, using several rotating rates (100, 250, 500 and 1000 s^{-1}) in potential window from 0 to -3.0 V. In the diagnostic window there is the record obtained during lowest rotation rate speed (100 s^{-1}).

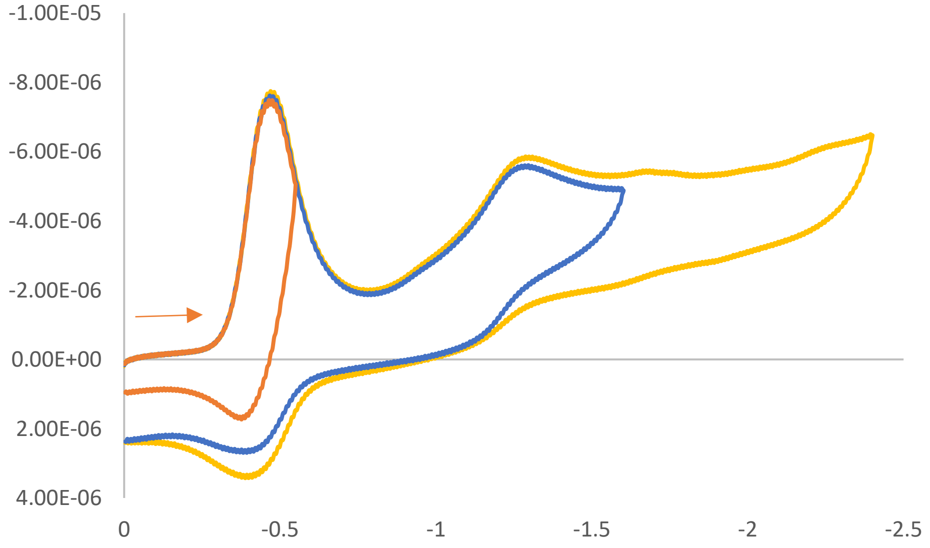

Figure S59: Reductive cyclic voltammogram (W = GC, Ref = SCE, Aux = Pt) of compound **8** (1.00 mM) in DMSO (0.1 M TBAHPF$_6$) measured with scan rate of 100 mV/s (polarographic plotting convention).

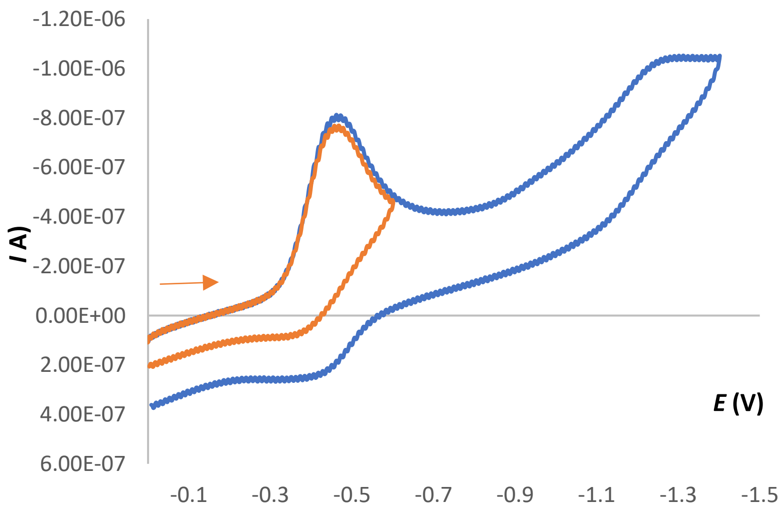

Figure S60: Reductive cyclic voltammogram (W = Pt, Ref = SCE, Aux = Pt) of compound **8** (1.00 mM) in DMSO (0.1 M $TBAHPF_6$) measured with scan rate of 100 mV/s (polarographic plotting convention).

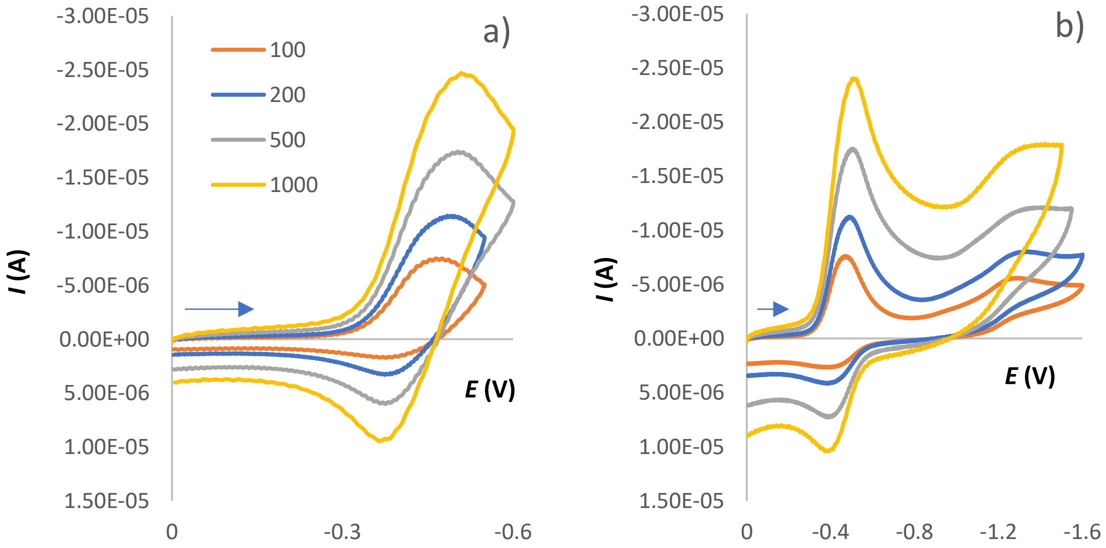

Figure S61: Reductive cyclic voltammograms (W = GC, Ref = SCE, Aux = Pt) of the 1st (a) and 2nd (b) peak of compound **8** (1.00 mM) in DMSO (0.1 M $TBAHPF_6$) measured with several different scan rates (polarographic plotting convention).

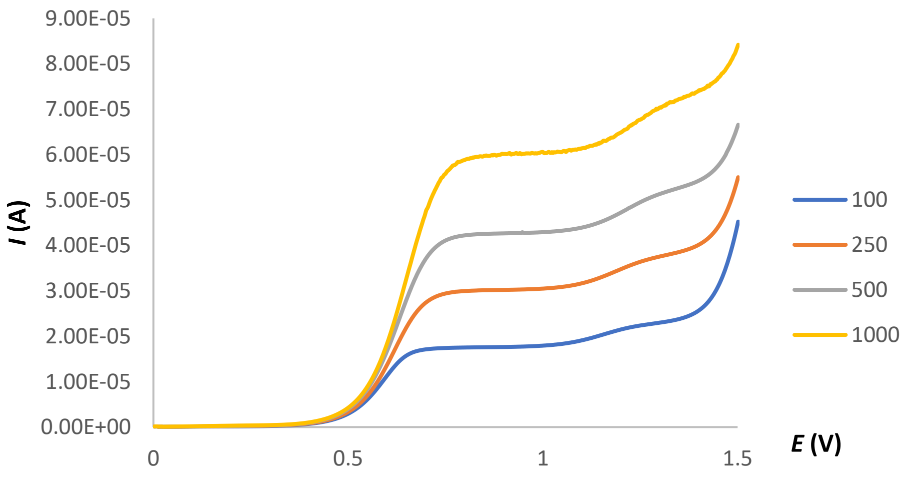

Figure S62: Linear sweep voltammetry of 2,6-dimethylhydroquinone (1.71 mM) in DMSO (0.1 M $TBAHPF_6$) (W = RDE = GC; Ref = SCE; Aux = Pt) was measured with scan rate of 10 mV/s, using several rotating rates (100, 250, 500 and 1000 s^{-1}) in potential window from 0 to 1.5 V.

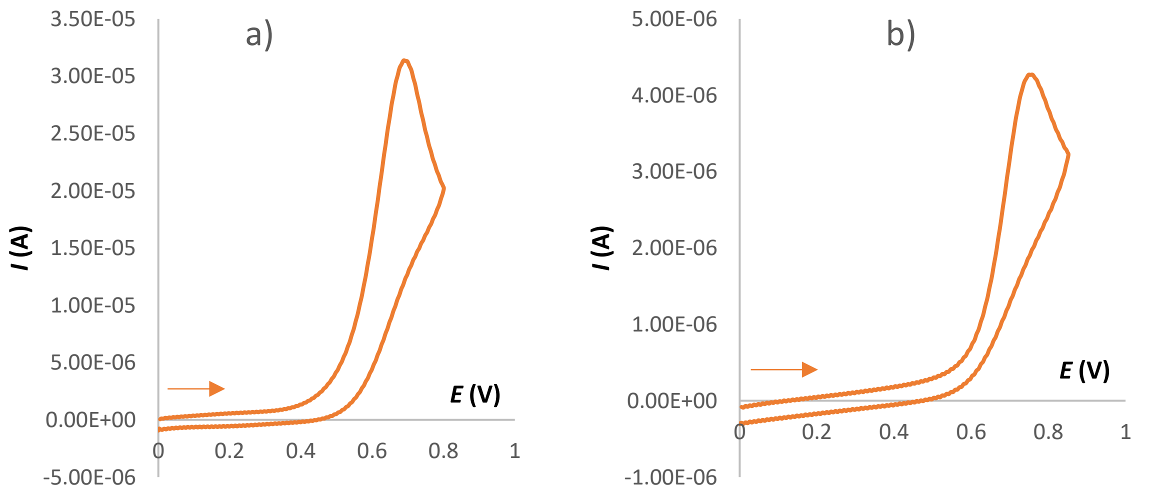

Figure S63: Oxidative cyclic voltammogram of 2,6-dimethylhydroquinone (1.71 mM) in DMSO (0.1 M $TBAHPF_6$) measured with scan rate of 100 mV/s (IUPAC plotting convention). For Graf a) (W = GC, Ref = SCE, Aux = Pt); Graf b) (W = Pt, Ref = SCE, Aux = Pt).

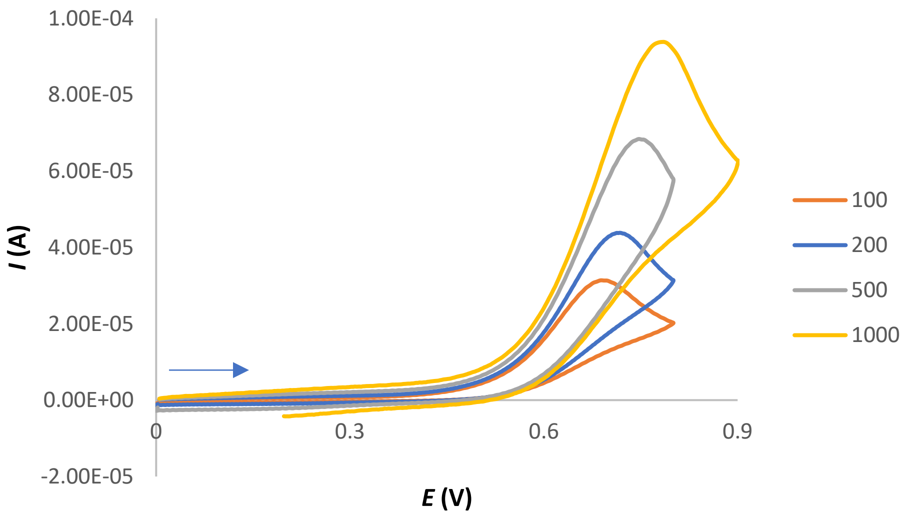

Figure S64: Oxidative cyclic voltammograms (W = GC, Ref = SCE, Aux = Pt) of 2,6-dimethylhydroquinone (1.71 mM) in DMSO (0.1 M $TBAHPF_6$) measured with several different scan rates (IUPAC plotting convention).

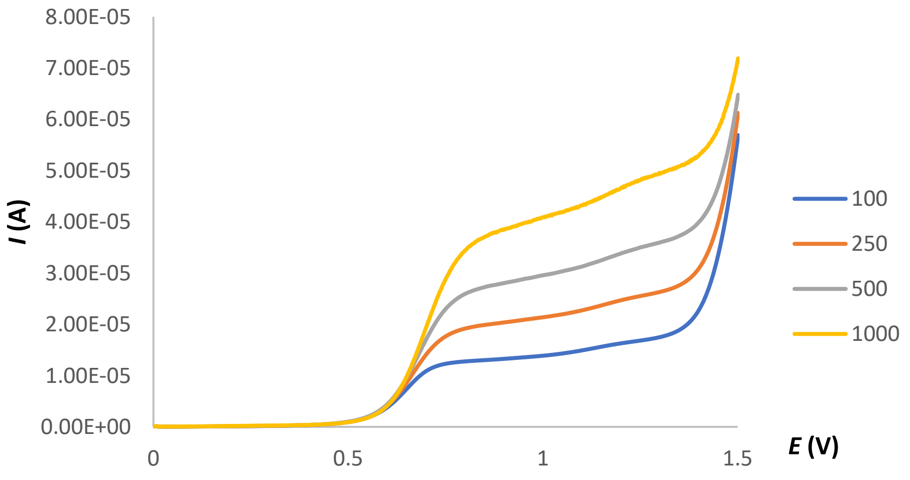

Figure S65: Linear sweep voltammetry of compound **7** (1.03 mM) in DMSO (0.1 M $TBAHPF_6$) (W = RDE = GC; Ref = SCE; Aux = Pt) was measured with scan rate of 10 mV/s, using several rotating rates (100, 250, 500 and 1000 s^{-1}) in potential window from 0 to 1.5 V.

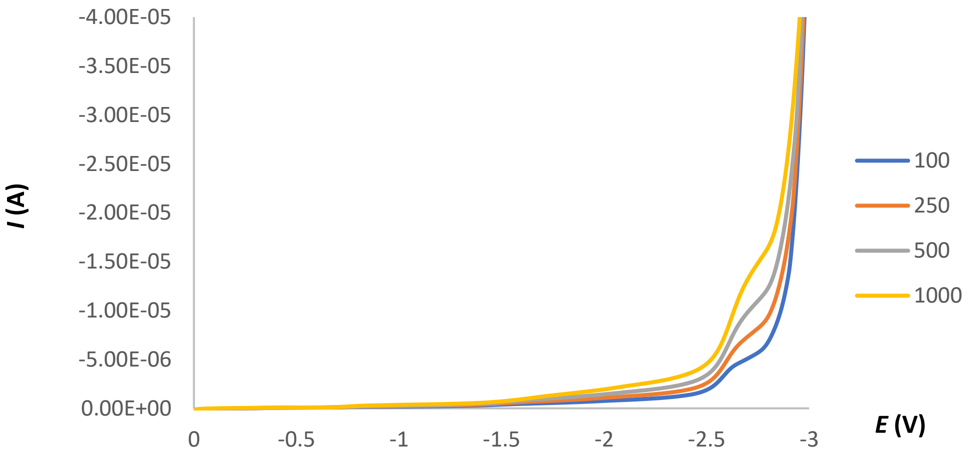

Figure S66: Linear sweep voltammetry of compound **7** (1.03 mM) in DMSO (0.1 M $TBAHPF_6$) (W = RDE = GC; Ref = SCE; Aux = Pt) was measured with scan rate of 10 mV/s, using several rotating rates (100, 250, 500 and 1000 s^{-1}) in potential window from 0 to -3.0 V.

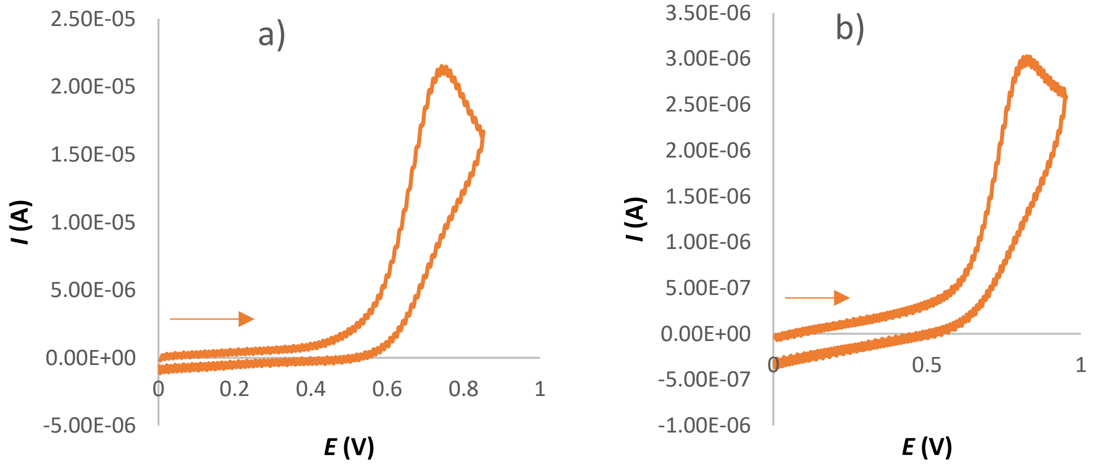

Figure S67: Oxidative cyclic voltammogram of compound **7** (1.03 mM) in DMSO (0.1 M $TBAHPF_6$) measured with scan rate of 100 mV/s (IUPAC plotting convention). For Graf a) (W = GC, Ref = SCE, Aux = Pt); Graf b) (W = Pt, Ref = SCE, Aux = Pt).

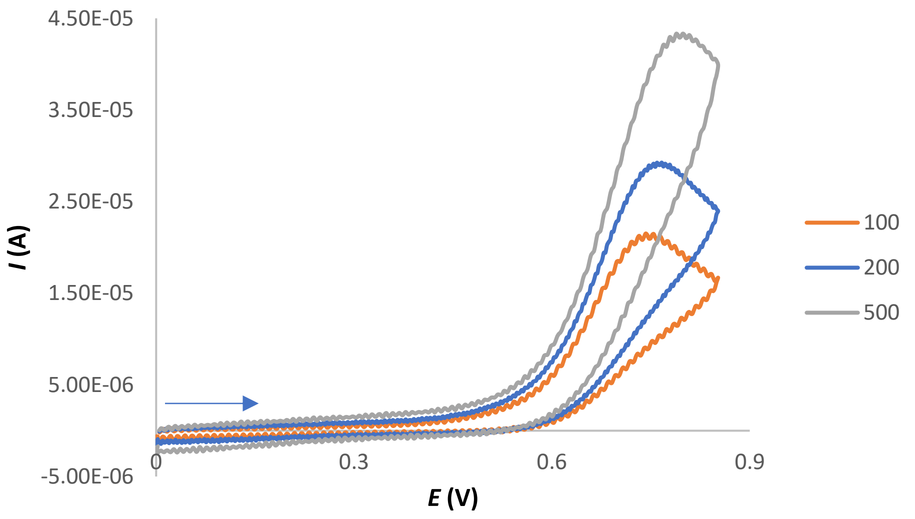

Figure S68: Oxidative cyclic voltammograms (W = GC, Ref = SCE, Aux = Pt) of compound **7** (1.03 mM) in DMSO (0.1 M $TBAHPF_6$) measured with several different scan rates (IUPAC plotting convention).

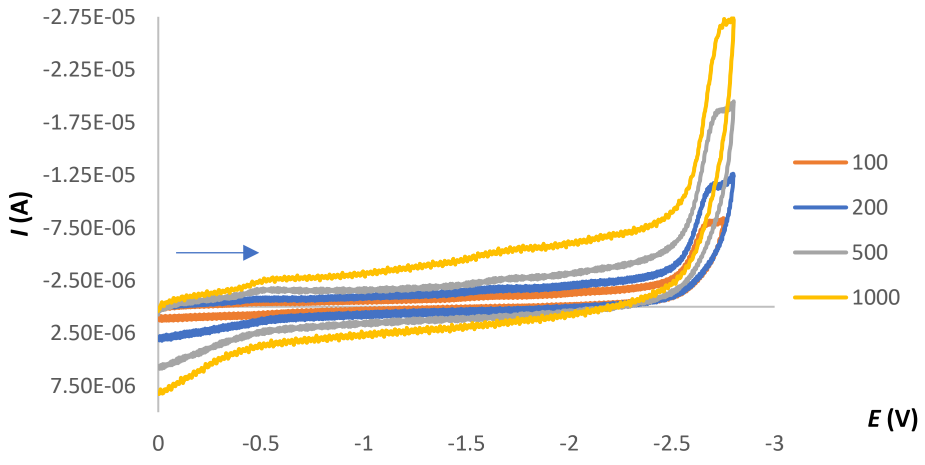

Figure S69: Reductive cyclic voltammograms (W = GC, Ref = SCE, Aux = Pt) of macrocycle **7** (1.03 mM) in DMSO (0.1 M $TBAHPF_6$) measured with several different scan rates (polarographic plotting convention).

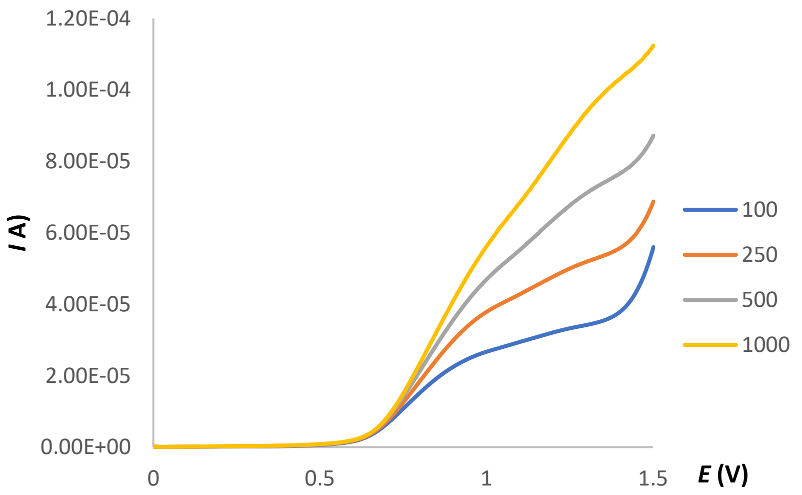

Figure S70: Linear sweep voltammetry of macrocycle **9** (1.06 mM) in DMSO (0.1 M $TBAHPF_6$) (W = RDE = GC; Ref = SCE; Aux = Pt) was measured with scan rate of 10 mV/s, using several rotating rates (100, 250, 500 and 1000 s^{-1}) in potential window from 0 to 1.5 V.

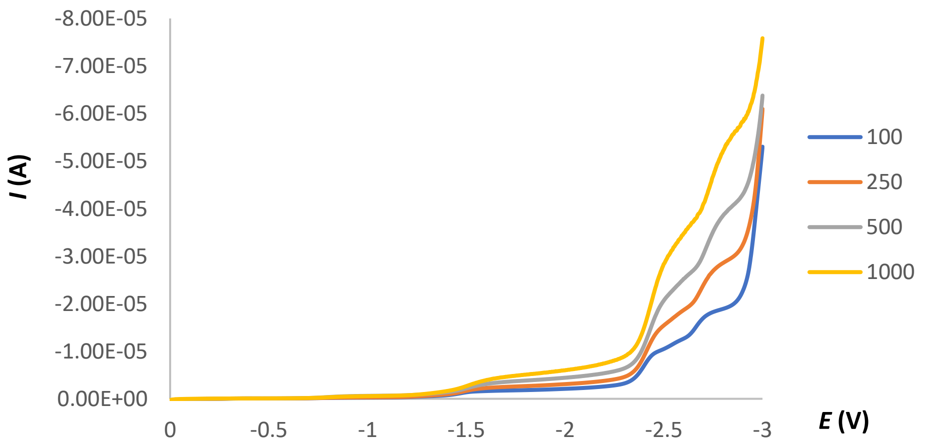

Figure S71: Linear sweep voltammetry of macrocycle **9** (1.06 mM) in DMSO (0.1 M $TBAHPF_6$) (W = RDE = GC; Ref = SCE; Aux = Pt) was measured with scan rate of 10 mV/s, using several rotating rates (100, 250, 500 and 1000 s^{-1}) in potential window from 0 to -3.0 V.

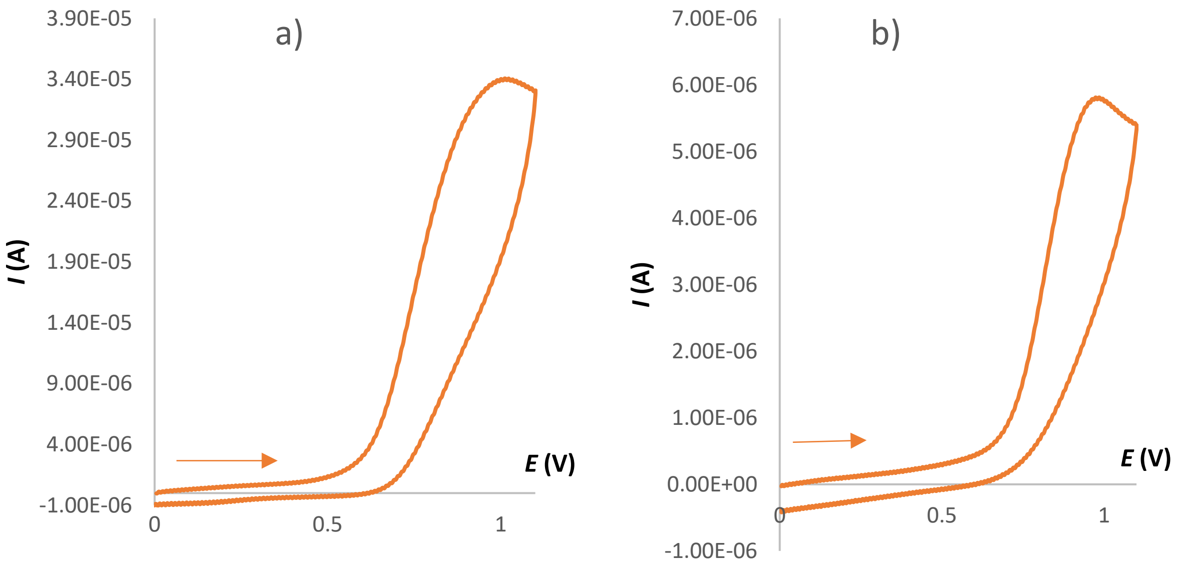

Figure S72: Oxidative cyclic voltammogram of macrocycle **9** (1.06 mM) in DMSO (0.1 M TBAHPF$_6$) measured with scan rate of 100 mV/s (IUPAC plotting convention). For Graf a) (W = GC, Ref = SCE, Aux = Pt); Graf b) (W = Pt, Ref = SCE, Aux = Pt).

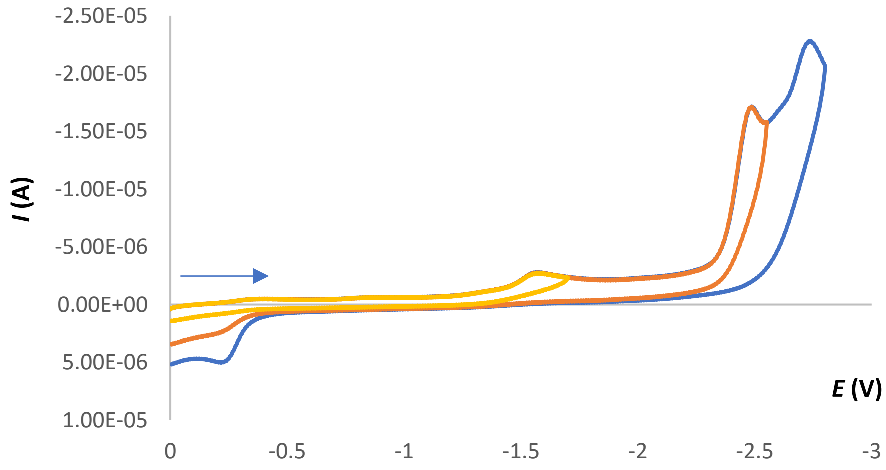

Figure S73: Reductive cyclic voltammogram (W = GC, Ref = SCE, Aux = Pt) of macrocycle **9** (1.09 mM) in DMSO (0.1 M TBAHPF$_6$) measured with scan rate of 100 mV/s (polarographic plotting convention).

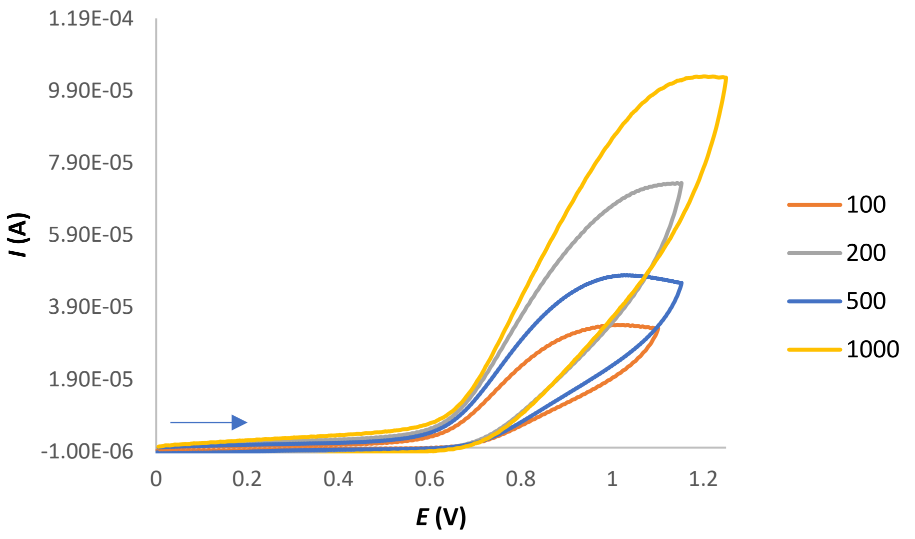

Figure S74: Oxidative cyclic voltammogram (W = GC, Ref = SCE, Aux = Pt) of macrocycle **9** (1.06 mM) in DMSO (0.1 M $TBAHPF_6$) measured with several different scan rates (IUPAC plotting convention).

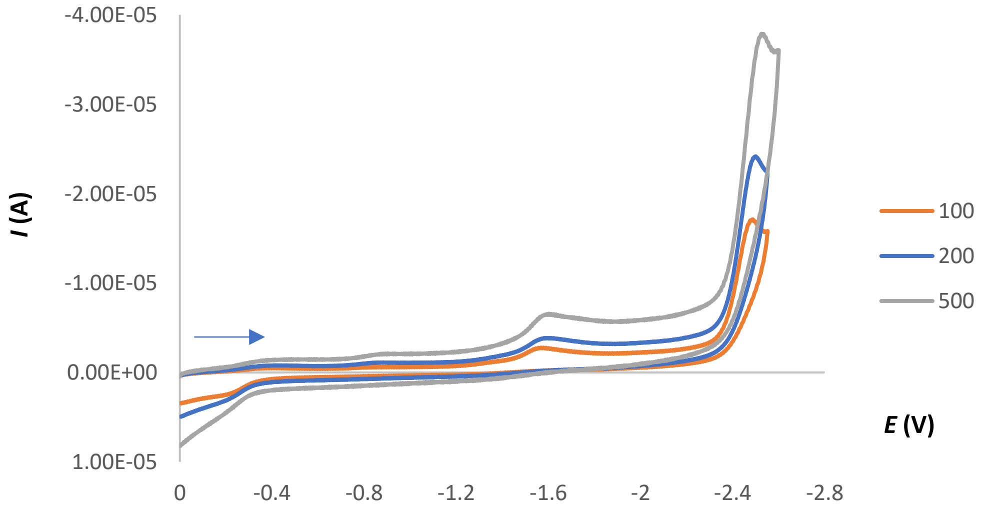

Figure S75: Reductive cyclic voltammogram (W = GC, Ref = SCE, Aux = Pt) of macrocycle **9** (1.06 mM) in DMSO (0.1 M $TBAHPF_6$) measured with several different scan rates (polarographic plotting convention).

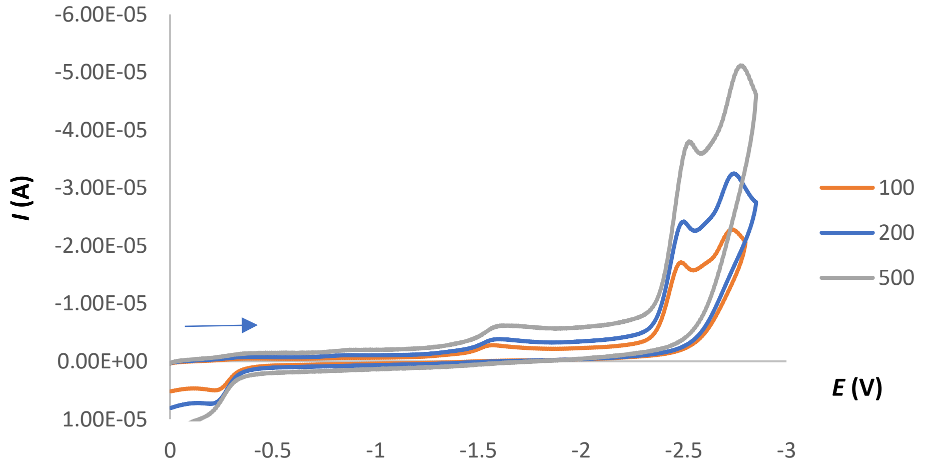

Figure S76: Reductive cyclic voltammogram (W = GC, Ref = SCE, Aux = Pt) of macrocycle **9** (1.06 mM) in DMSO (0.1 M TBAHPF$_6$) measured with several different scan rates (polarographic plotting convention).

5. X-ray data

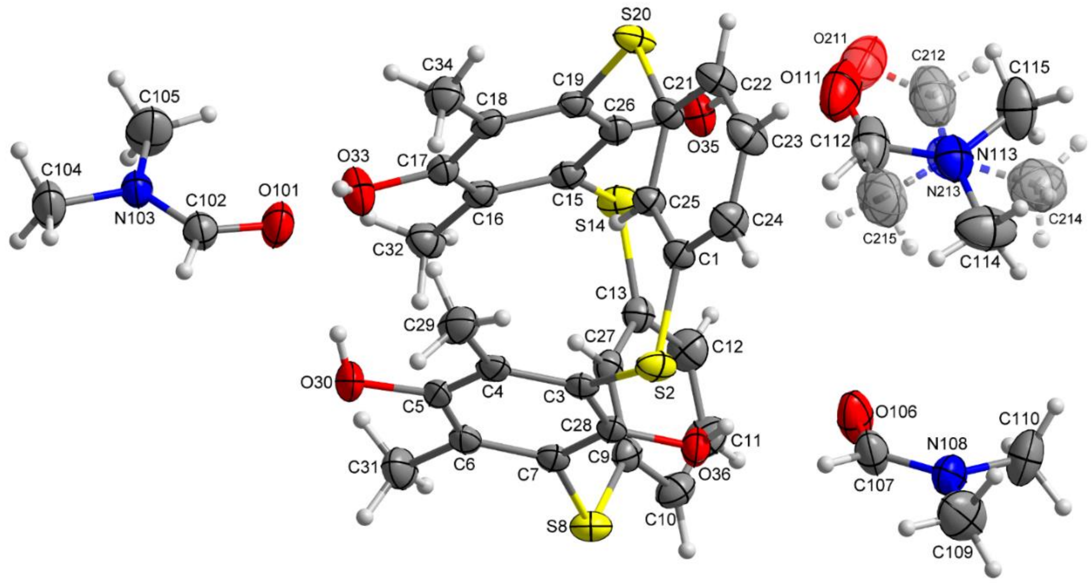

Figure S77. The numbering scheme for the crystal structure of compound **9**, the ADPs drawn at 50% probability level. The weakly occupied atoms depicted as transparent.

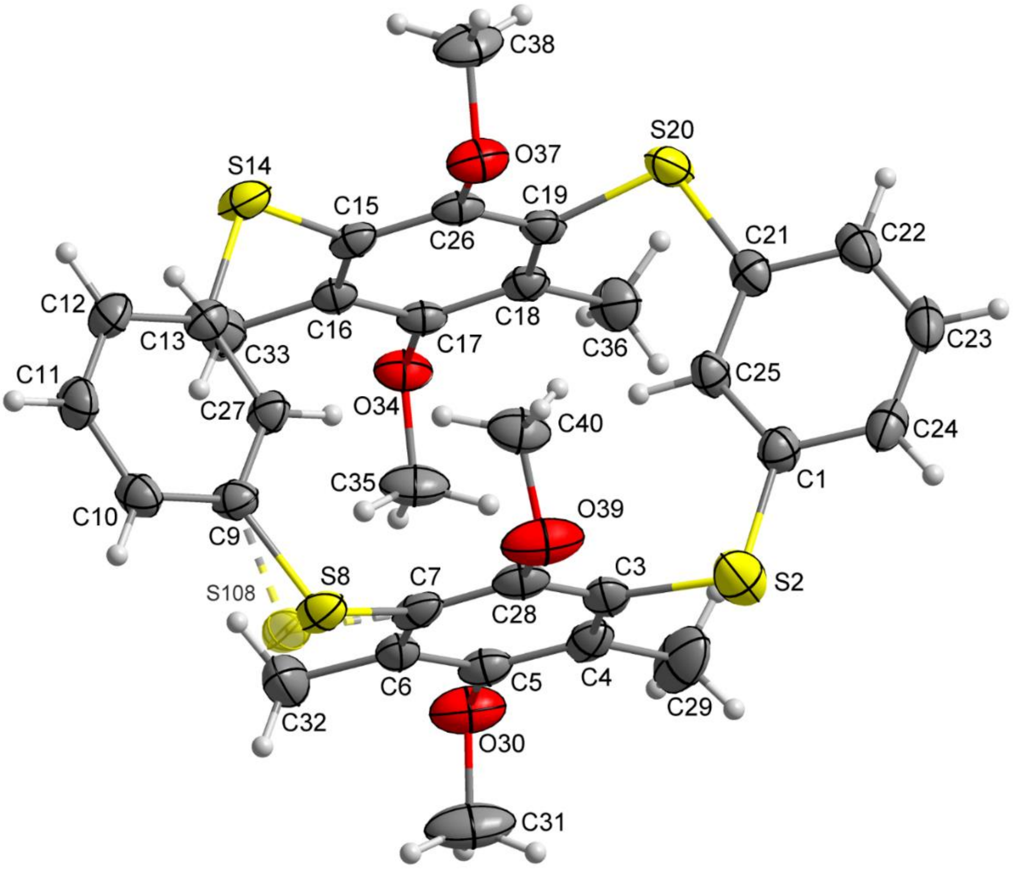

Figure S78. The numbering scheme for the crystal structure of compound **10a**, the ADPs drawn at 50% probability level. The weakly occupied atoms depicted as transparent.

Crystallographic data for compound **10a2** (polymorphic form obtained from $CHCl_3$)

M_r = 648.66, monoclinic system, space group $R\bar{3}c$, a = 22.2163(3) Å, b = 22.2163(3) Å, c = 33.5650(8) Å, γ = 120°, Z = 18, V = 14347.0(5) Å^3, D_c = 1.35 g cm^{-3}, μ(Cu-$K\alpha$) = 3.78 mm^{-1}, crystal dimensions of 0.09 × 0.14 × 0.21 mm. Data were collected at 180 (2) K on a D8 Venture Photon II 7 diffractometer with Incoatec microfocus sealed tube Cu-$K\alpha$ radiation. Due to the small crystal size, the total exposure time was 65 hours. Data reduction, scaling and absorption correction were performed using Apex4.[3] The structure was solved by charge-flipping methods[4] and refined anisotropically by full-matrix least squares on F^2 in the CRYSTALS programs[5] yielding final values of R = 0.058 and wR = 0.180, using 3147 independent reflections (ϑ_{max} = 72.3°), 214 parameters and 98 restraints. Hydrogen atoms bonded to carbon atoms were placed in calculated positions and refined with riding constraints. The chloroform solvent was found to be disordered along a three-fold axis near an inversion center in the solvent channel. The disorder model included four chlorine atoms positions. The disordered solvent atoms were located in difference electron density maps and refined with restrained geometry. The disordered hydrogen atom was not included in the model. The occupancies of the chlorine atoms were initially refined, then fixed at 0.15 and the carbon atom occupancy was fixed on 0.5. The resulting ratio of chloroform to **10a** was 1:3. MCE[6] was used for visualization of the electron density maps. The structure was deposited into Cambridge Structural Database under number CCDC 2422747.

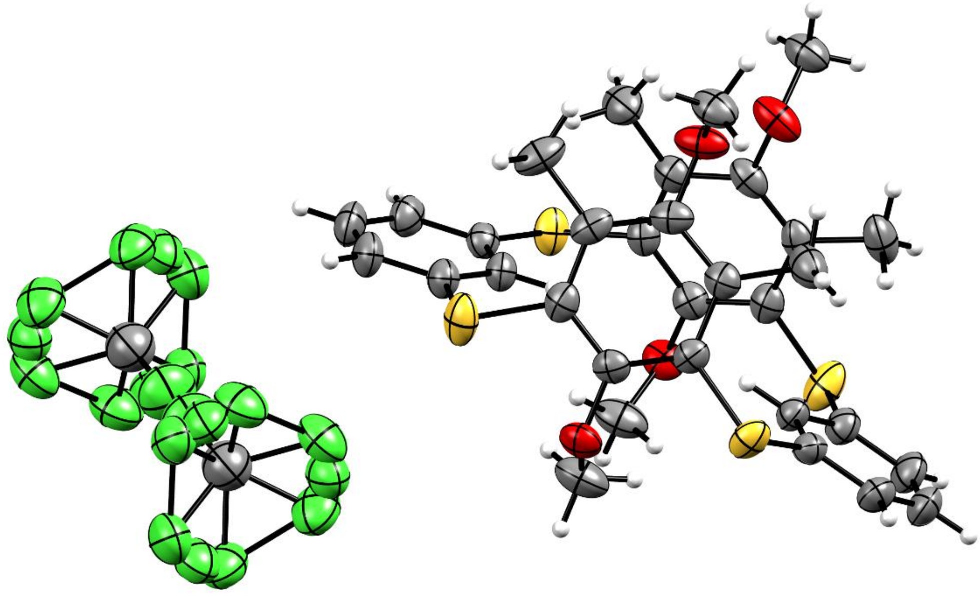

Figure S79. The crystal structure of compound **10a2** with solvent (disordered $CHCl_3$), the ADPs drawn at 50% probability level.

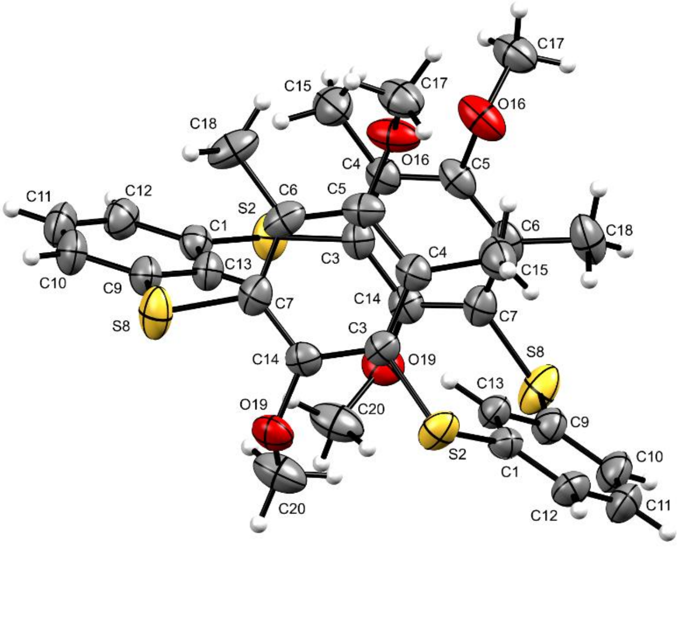

Figure S80. The numbering scheme for the crystal structure of compound **10a2**, the ADPs drawn at 50% probability level.

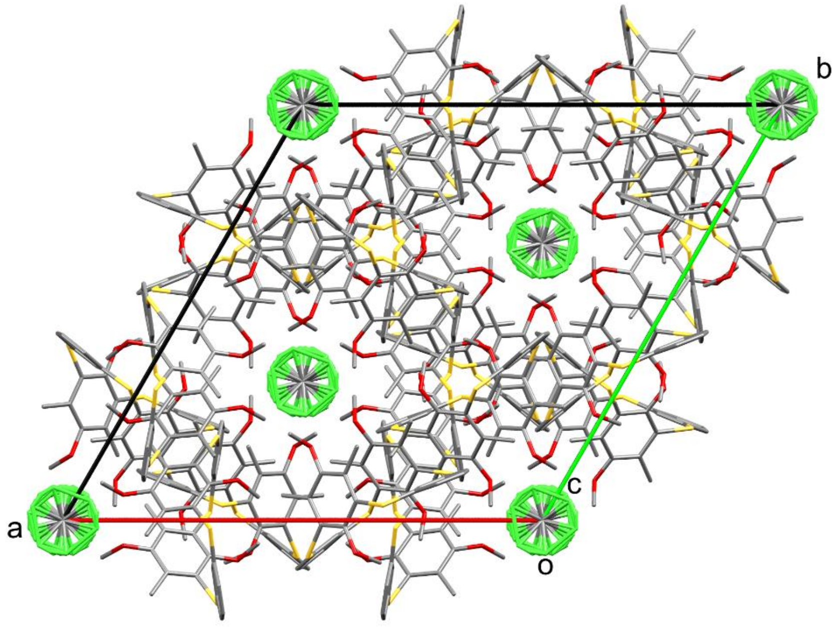

Figure S81. The unit cell packing of compound **10a2** along the c axis.

Crystallographic data for compound **10b**

M_r = 861.00, triclinic system, space group $P\bar{1}$ a = 12.4199(5) Å, b = 16.8454(7) Å, c = 17.2192(7) Å, α = 83.519(2)°, β = 77.804(2)°, γ = 86.919(2)°, Z = 4, V = 3497.1(2) Å^3, D_c = 1.29 g cm^{-3}, μ(Cu-$K\alpha$) = 2.81 mm^{-1}, crystal dimensions of 0.06 × 0.09 × 0.44 mm. Data were collected at 180 (2) K on a D8 Venture Photon II 7 diffractometer with Incoatec microfocus sealed tube Cu-$K\alpha$ radiation. Due to the small crystal size and low diffraction power, the total exposure time was 95 hours. Data reduction, scaling and absorption correction were performed using Apex4.[3] The structure was solved by Intrinsic Phasing methods[7] and refined anisotropically by full-matrix least squares on F^2 in the CRYSTALS programs[5] yielding final values of R = 0.073 and wR = 0.212, using 12588 independent reflections (ϑ_{max} = 68.2°), 811 parameters and no restraints. Hydrogen atoms bonded to carbon atoms were placed in calculated positions and refined with riding constraints. The OH hydrogen atom in the methanol solvent was not found and was placed geometrically to follow hydrogen bond direction. MCE[6] was used for visualization of the electron density maps. The structure was deposited into Cambridge Structural Database under number CCDC 2425166.

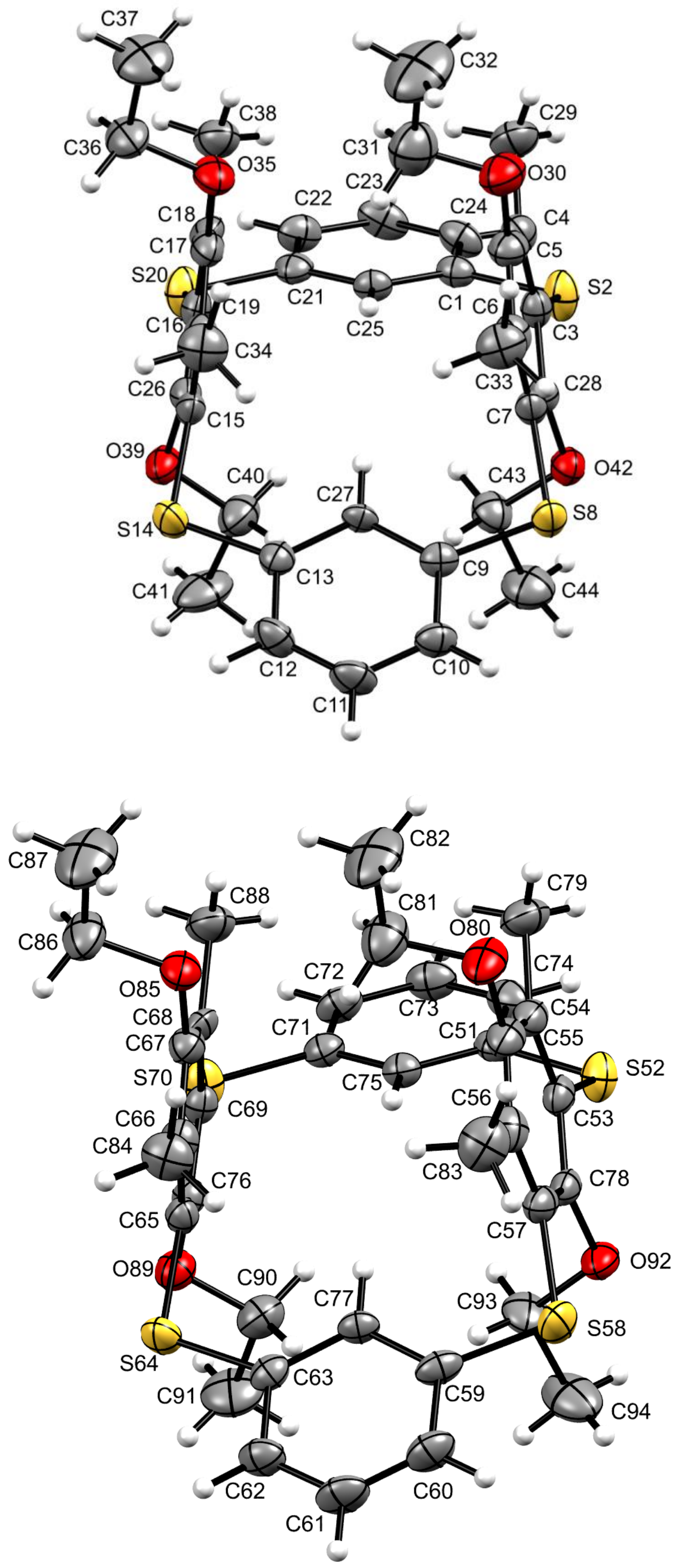

Figure S82. The numbering scheme for the crystal structure of compound **10b** (both independent molecules), the ADPs drawn at 50% probability level.

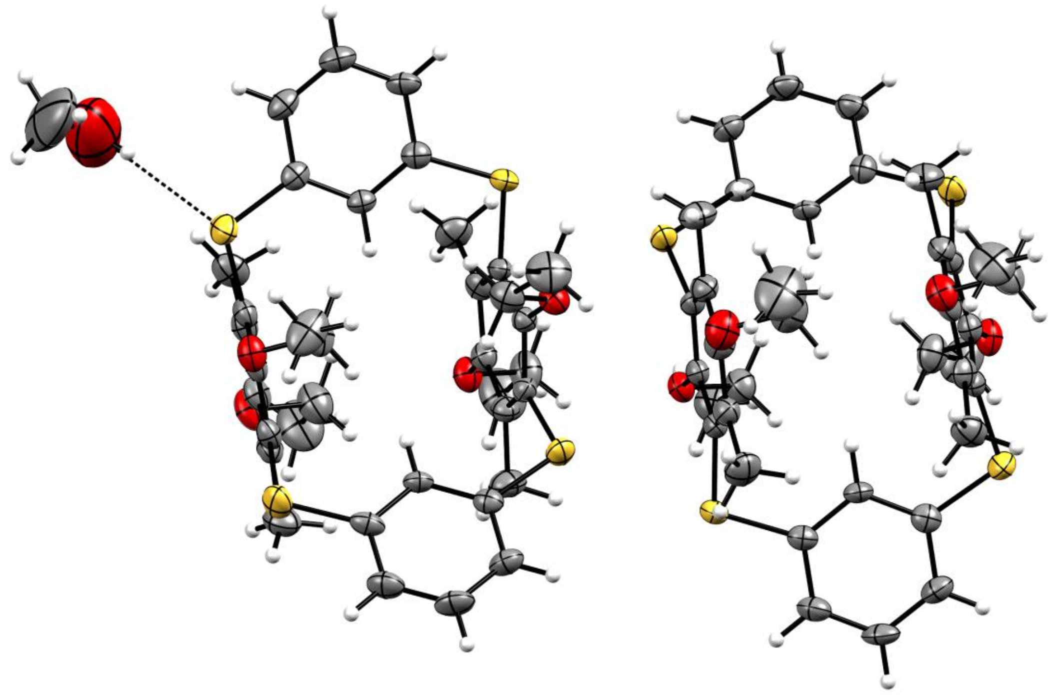

Figure S83. The crystal structure of compound **10b** (both independent molecules) with solvent, the ADPs drawn at 50% probability level.

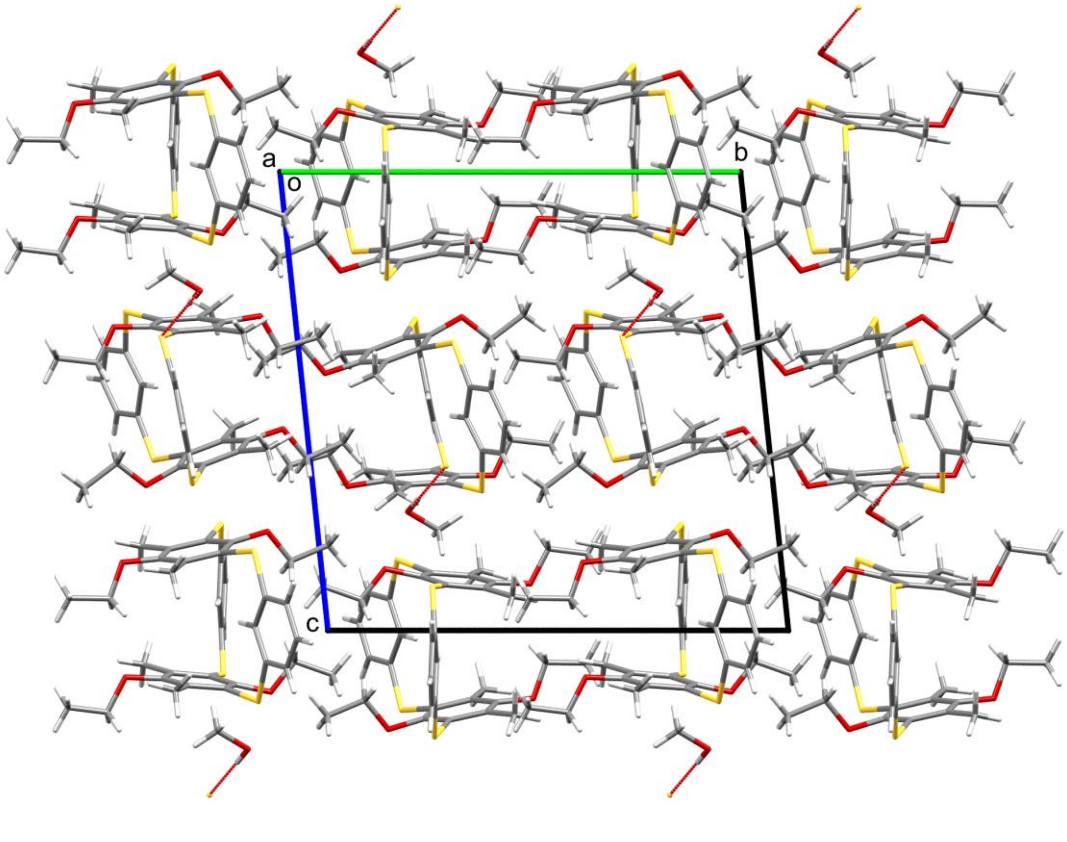

Figure S84. The unit cell packing of compound **10b** along the c axis.

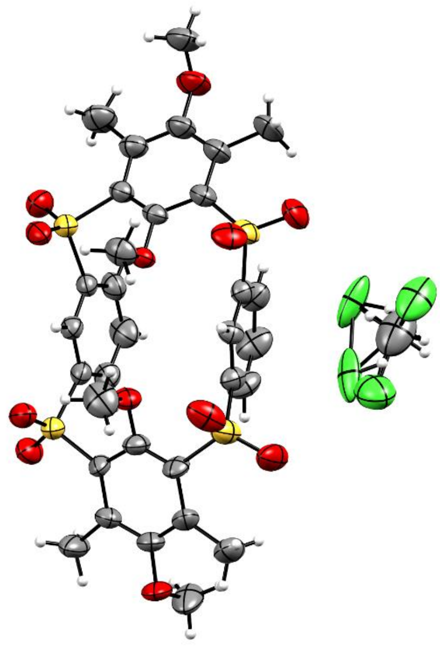

Figure S85. The crystal structure of compound **11** with solvent (disordered $CHCl_3$), the ADPs drawn at 50% probability level.

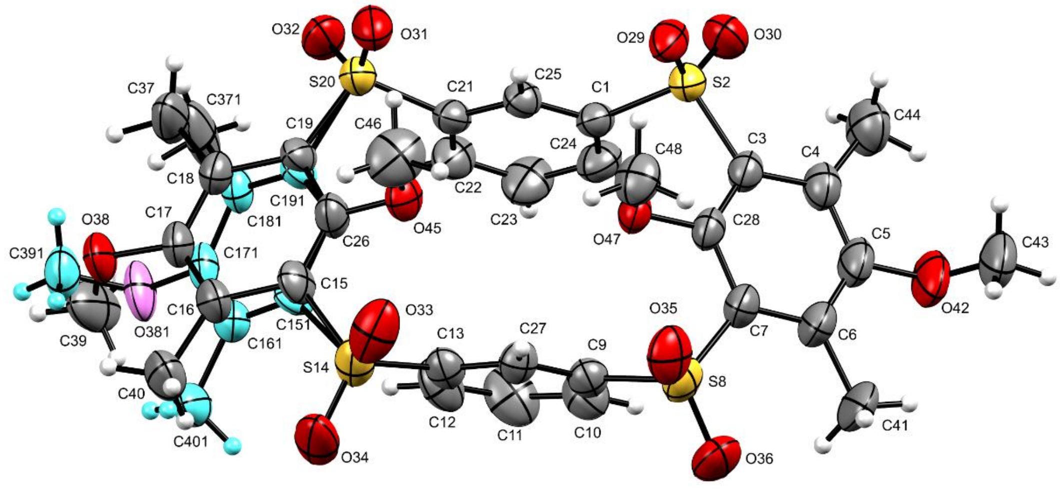

Figure S86. The numbering scheme for the crystal structure of compound **11**, the ADPs drawn at 50% probability level.

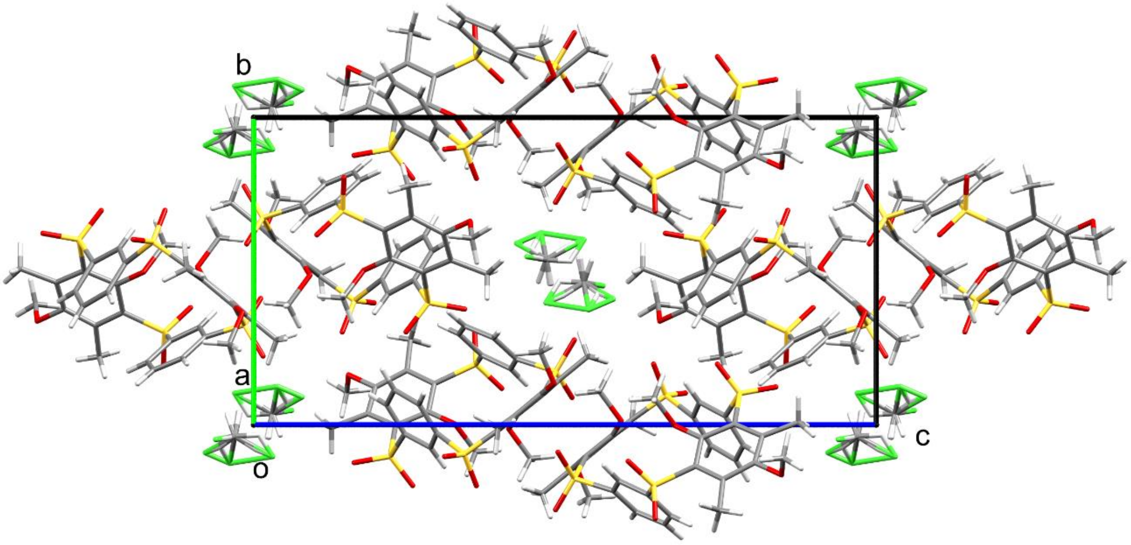

Figure S87. The unit cell packing of compound **11** along the a axis.

6. Literature

(1) Kurth, H.-J.; Kraatz, U.; Korte, F. Zur Synthese von 1,3-Dimercaptobenzolen aus Resorcinen über die Newman-Kwart-Umlagerung. *Chem. Ber.* **1973**, *106* (8), 2419-2426. DOI:

(2) Maiolo, F.; Testaferri, L.; Tiecco, M.; Tingoli, M. Fragmentation of aryl alkyl sulfides. A simple, one-pot synthesis of polymercaptobenzenes from polychlorobenzenes. *J. Org. Chem.* **1981**, *46* (15), 3070-3073.

(3) *Bruker (2021). APEX4, SAINT and SADABS. Bruker AXS Inc., Madison, Wisconsin, USA.*; (accessed.

(4) Palatinus, L.; Chapuis, G. SUPERFLIP–a computer program for the solution of crystal structures by charge flipping in arbitrary dimensions. *J. Appl. Crystallogr.* **2007**, *40* (4), 786-790.

(5) Betteridge, P. W.; Carruthers, J. R.; Cooper, R. I.; Prout, K.; Watkin, D. J. CRYSTALS version 12: software for guided crystal structure analysis. *J. Appl. Crystallogr.* **2003**, *36* (6), 1487.

(6) Rohlicek, J.; Husak, M. MCE2005 - a new version of a program for fast interactive visualization of electron and similar density maps optimized for small molecules. *J. Appl. Crystallogr.* **2007**, *40* (3), 600-601.

(7) Sheldrick, G. M. SHELXT–Integrated space-group and crystal-structure determination. *Acta Crystallographica Section A: Foundations and Advances* **2015**, *71* (1), 3-8.
